# Supplementary material for: TYK2 mediates neuroinflammation in Alzheimer’s disease brains with TDP-43 pathology
Source: Nat Commun. 2026 Mar 14;17:3967. doi: 10.1038/s41467-026-70243-3 (PMC13133158; doi:10.1038/s41467-026-70243-3)
Supplement: Supplementary file 1 — Supplementary Information [file 41467_2026_70243_MOESM1_ESM.pdf]

TYK2 mediates neuroinflammation in Alzheimer's disease brains with TDP-43 pathology

König *et al.*

SUPPLEMENTARY INFORMATION

SUPPLEMENTARY TABLE 1

Age at death	Sex	PMI [hours]	NPDX	CERAD (neuritic plaques)	Braak staging	2012 NIA-AA ABC scoring	Figure reference
63	male	16	healthy control	none	0	not met	Figure 1a (Healthy Control Case I), Supplementary Figure 1a (Control, Normal TDP-43)
≥90	male	24	healthy control	sparse	I	not met	Supplementary Figure 1a (Control, Normal TDP-43)
73	female	20	healthy control	none	0	not met	Supplementary Figure 1a (Control, Mild TDP-43)
≥90	female	8	healthy control	sparse	II	low	Supplementary Figure 1a (Control, Mild TDP-43)
65	male	unknown	healthy control	not assessed	NA	not met	Figure 1a (Healthy Control Case II), Figure 1b (IHC of Healthy Control), Supplementary Figure 1a (Control, Normal TDP-43), Supplementary Figure 1b (Normal)
76	female	7	Alzheimer's Disease	frequent	VI	high	Supplementary Figure 1a (AD, Severe TDP-43)
≥90	female	16	Alzheimer's Disease	moderate	VI	high	Figure 1a (AD Case I), Figure 1b (IHC of AD), Supplementary Figure 1a (AD, Severe TDP-43), Supplementary Figure 1b (Severe)
68	male	27	healthy control	none	I	not met	Supplementary Figure 1a (Control, Severe TDP-43)
82	male	16	Alzheimer's Disease	moderate	VI	high	Figure 1a (AD Case II), Figure 1b (IF of AD), Supplementary Figure 1a (AD, Severe TDP-43), Supplementary Figure 1b (Mild), Supplementary Figure 2a,b (AD)
86	female	unknown	Alzheimer's Disease	moderate	IV	moderate	Supplementary Figure 1a (AD, Severe TDP-43)
80	male	4	Alzheimer's Disease	moderate	V	high	Supplementary Figure 1a (AD, Severe TDP-43)
76	female	39	healthy control	sparse	III	low	Supplementary Figure 1a (Control, Normal TDP-43)
84	female	15	Alzheimer's Disease	frequent	VI	high	Figure 1a (AD Case III), Supplementary Figure 1a (AD, Severe TDP-43)
70	male	36	Alzheimer's Disease	moderate	V	high	Supplementary Figure 1a (AD, Mild TDP-43)
89	male	16	Alzheimer's Disease	moderate	V	high	Supplementary Figure 1a (AD, Severe TDP-43)
77	female	72	healthy control	sparse	I	low	Supplementary Figure 1a (Control, Normal TDP-43)
89	female	31	Alzheimer's Disease	moderate	VI	high	Supplementary Figure 1a (AD, Severe TDP-43)
≥90	male	23	healthy control	sparse	II	low	Figure 1a (Healthy Control Case III), Figure 1b (IF of Healthy Control), Supplementary Figure 1a (Control, Normal TDP-43), Supplementary Figure 2a (Control)
73	female	22	Alzheimer's Disease	moderate	VI	high	Supplementary Figure 1a (AD, Mild TDP-43)

NPDX = Neuropathology diagnosis
PMI = Postmortem Interval
CERAD = The Consortium to Establish a Registry for Alzheimer's Disease

SUPPLEMENTARY FIGURES

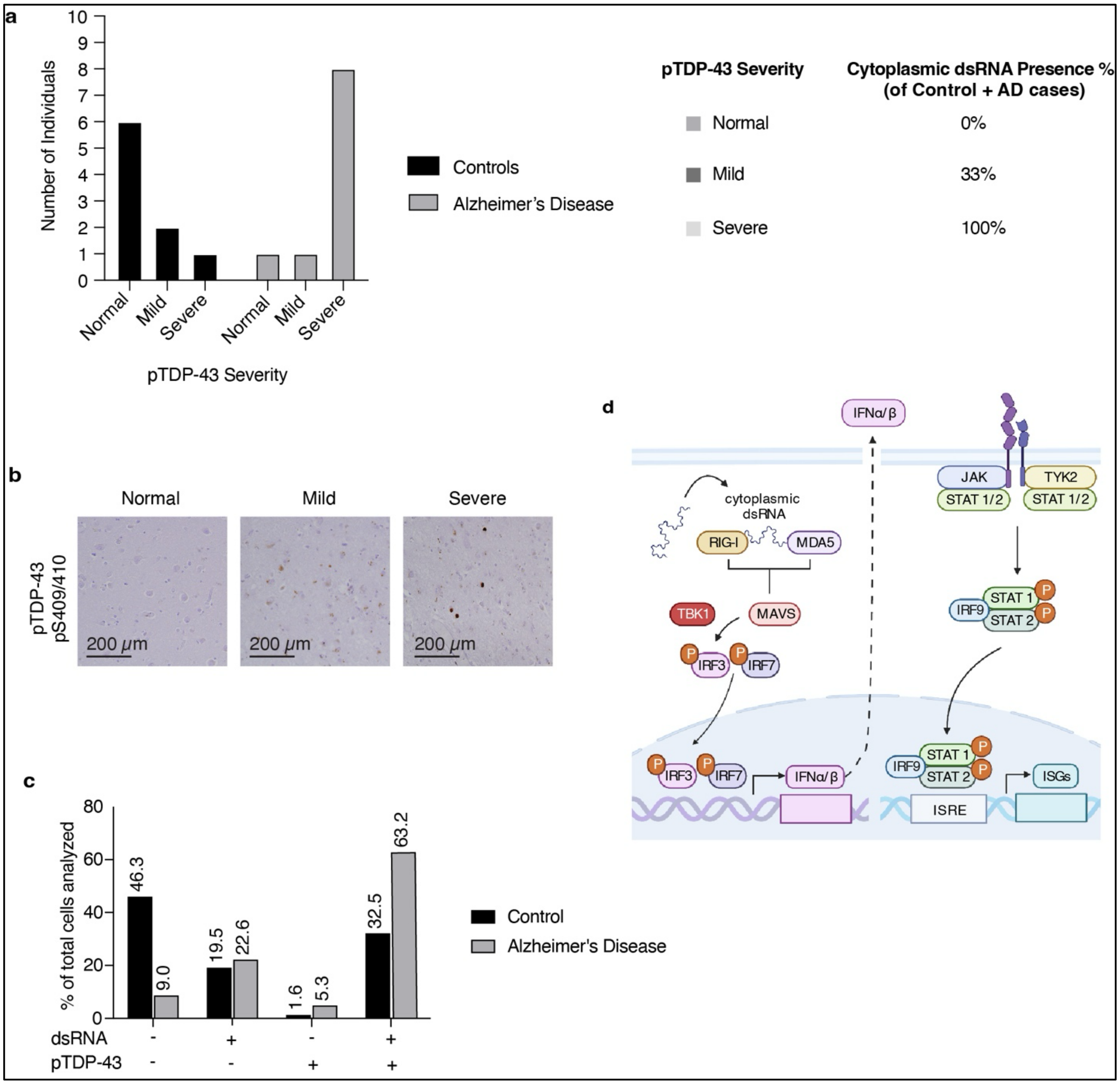

Supplementary Fig. 1: pTDP-43-severity and dsRNA presence in human postmortem brain sections. a, Histogram of assessed formalin-fixed paraffin-embedded (FFPE) amygdala sections (n = 10 with Alzheimer's Disease (AD), n = 9 healthy controls) that show normal, mild, and severe pTDP-43 severity compared to healthy controls as well as the coincidence with dsRNA in percent of cases (control + AD cases). **b,** Histological examples for the classification of pTDP-43 severity into normal, mild, and severe (amygdala sections). **c,** Proportion of cells that stained for cdsRNA, pTDP-43, or both (total number of assessed cells for control = 123 and for AD = 133). Source Data are provided as a Source Data file. **d,** Pathway schematic of innate immune response to cdsRNA in AD leading to the expression of interferon-stimulated genes. Created in BioRender. Daneshvari, S. (2026) https://BioRender.com/7fm5vk9

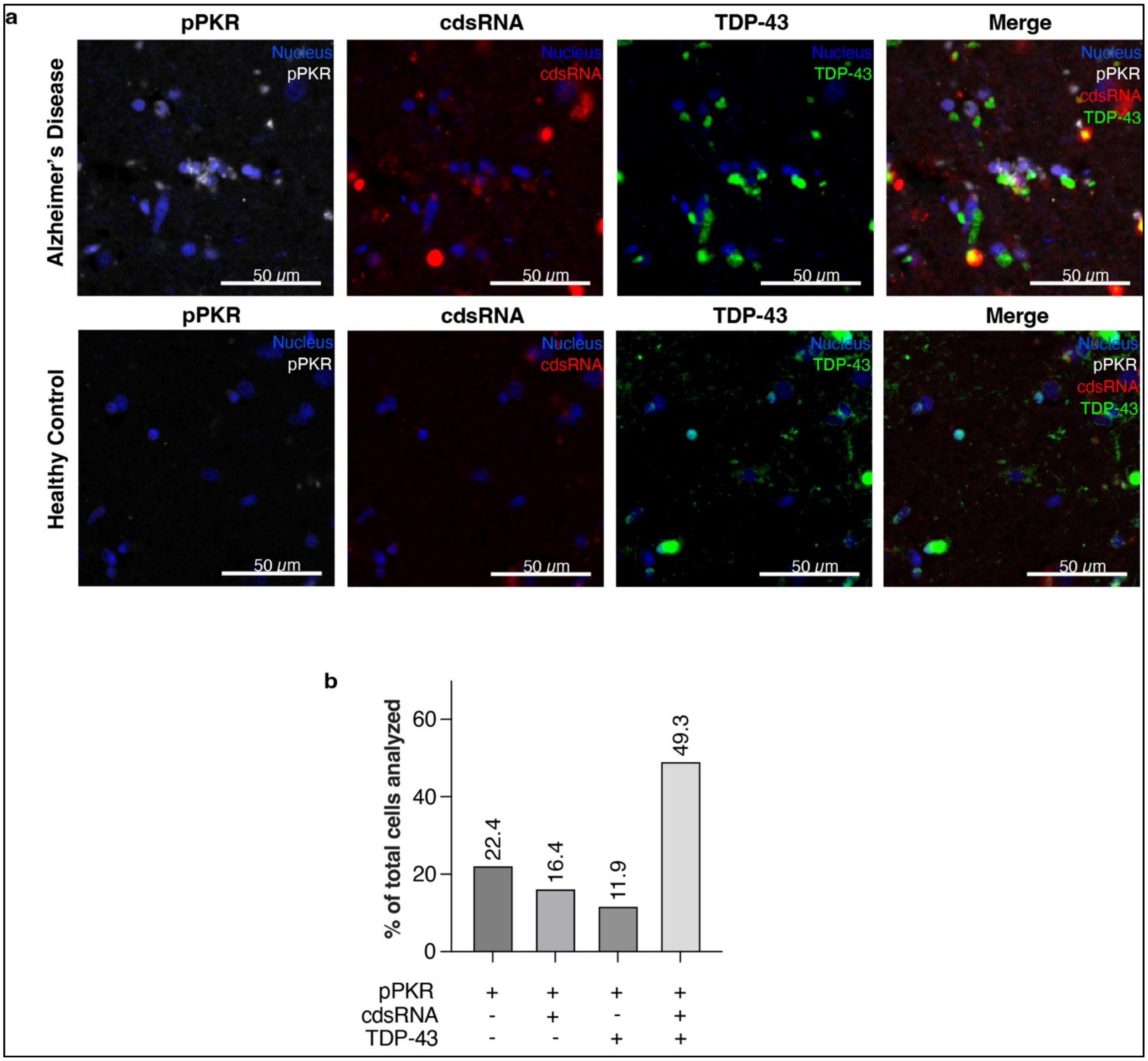

Supplementary Fig. 2: Type-I interferon response to cdsRNA assessed by PKR activation. a, Cyclic immunofluorescence (CyCIF) staining of human postmortem brain sections of the amygdala comparing the phosphorylation of PKR (white) alongside cdsRNA (red) and TDP-43 (green) in an Alzheimer's Disease brain (top) compared to a healthy control (bottom) as proof of the immunogenicity of cdsRNA. Sytox Blue used to stain nuclei (blue). Neuropathology diagnosis based on IHC staining by neuropathologist. This experiment included one controls brain and two AD patients. **b,** Proportion of pPKR-positive cells that co-stained for cdsRNA, pTDP-43, or both in a randomly selected site (total number of assessed cells = 67). Source Data are provided as a Source Data file.

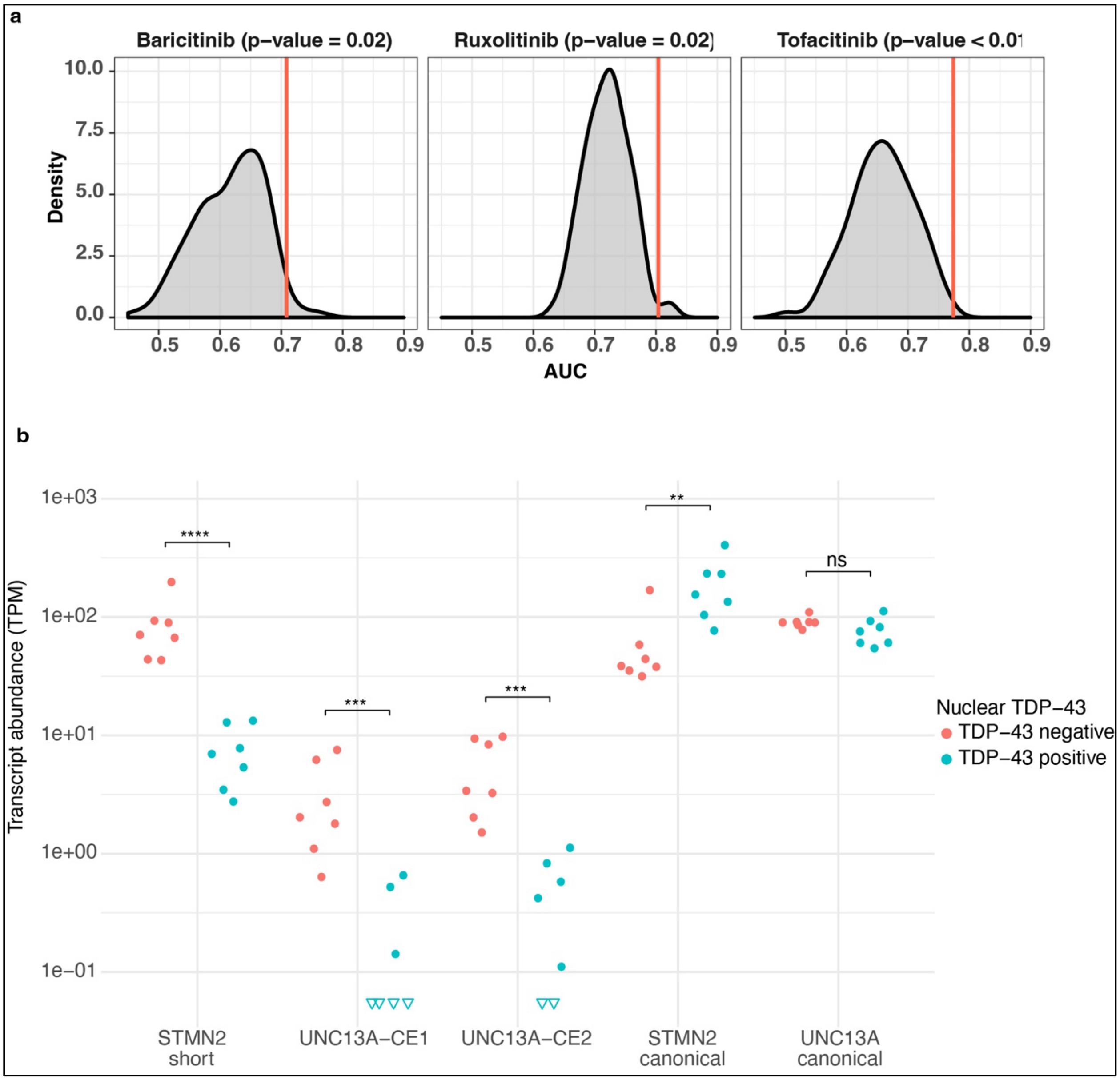

Supplementary Fig. 3: DRIAD-SP: Previous results and proof of concept for using cryptic exon (CE) expression as a proxy for TDP-43 pathology. a, Prediction of drug efficacy of three selected compounds in Alzheimer's disease using DRIAD-SP. Drug efficacy was assessed according to the previously published protocol[23]. Red lines indicate the DRIAD-SP model performance of the drug gene sets, whereas the gray shaded regions correspond to the distribution of model performances based on size-matched random gene sets. AUC = area under curve. Empirical one-sided *P* values were computed as the fraction of random background gene sets with higher AUC values than the drug-derived gene set. *P* values were corrected for multiple testing using the Benjamini-Hochberg procedure. **b,** Expression of TDP-43-associated transcripts in single neuronal nuclei as proof of concept for using CE expression as a proxy for TDP-43 pathology. Neuronal nuclei from amyotrophic lateral sclerosis patients' neocortical tissues were FACS-sorted by the presence of nuclear TDP-43 and NeuN[40]. Here, TDP-43-positive nuclei (blue), indicating normal levels of nuclear TDP-43, indicate cases without TDP-43 pathology, while TDP-43-negative nuclei (red) corresponded to cases with TDP-43 pathology. Each point corresponds to the abundance

(transcripts per million; TPM) of TDP-43-associated CE transcripts or their canonical counterparts in one of the samples. (STMN2 short $P < 0.0001$; UNC13A-CE1 $P = 0.0002$; UNC13A-CE2 $P = 0.0006$; STMN2 $P = 0.0017$; UNC13A $P = 0.11$). Triangles indicate zero counts. Statistical tests: unpaired t tests with two tails.

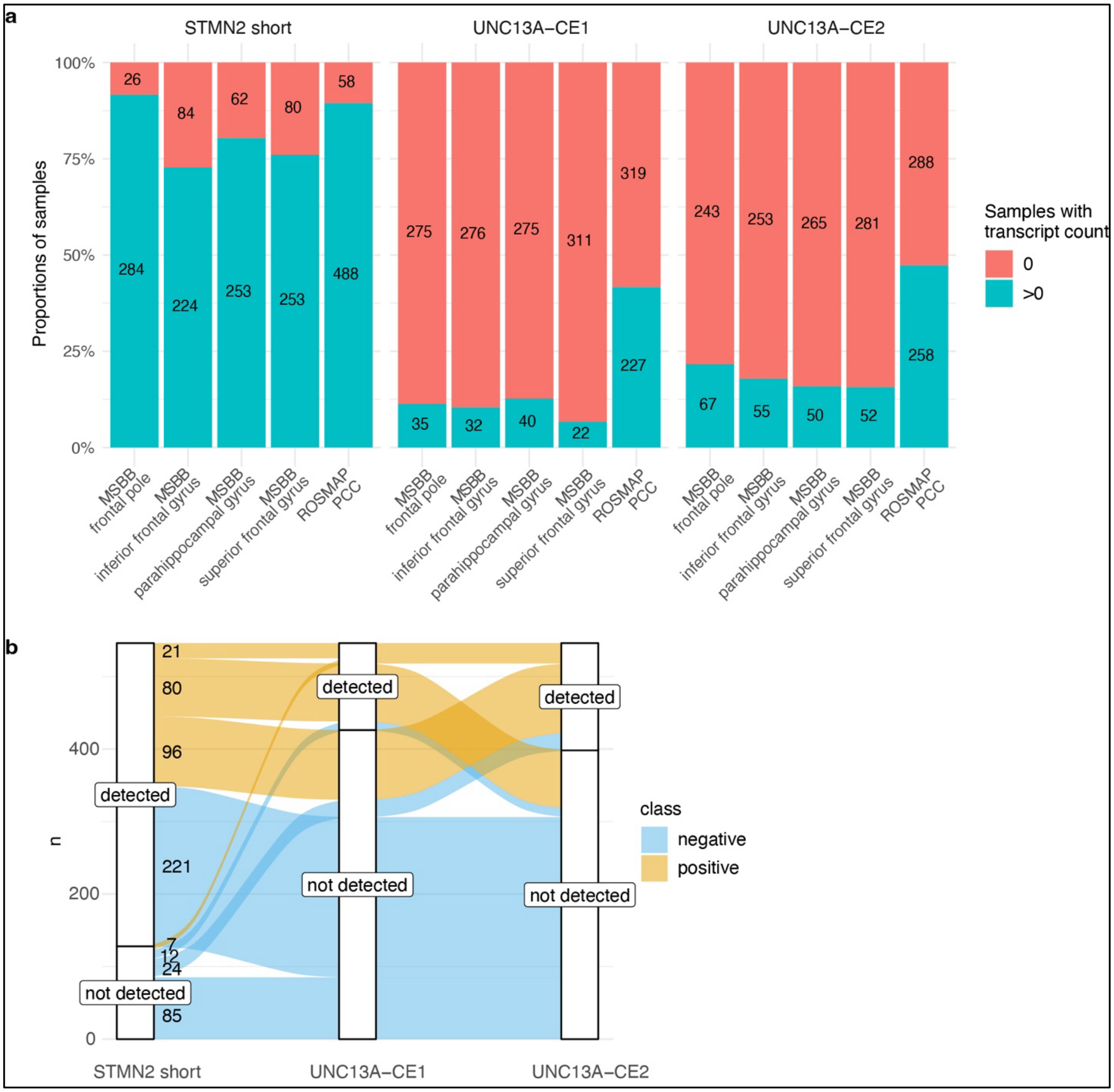

Supplementary Fig. 4: Evaluation of cryptic exon (CE) expression as an adequate proxy of TDP-43 pathology. a, The proportion of samples with zero (red) and above zero (blue) abundance (transcripts per million; TPM) of the given transcripts is shown. MSBB samples had an appreciable lower abundance of UNC13A-CE transcripts compared to ROSMAP samples. This difference can most likely be attributed to MSBB using single-end sequencing compared to the paired-end sequencing employed by ROSMAP, making detection of CEs and therefore determination of TDP-43 pathology in MSBB difficult. **b,** Alluvial diagram illustrating co-expression patterns of CEs across samples. Each alluvium (horizontal spline) represents a cohort of samples sharing the same

CE expression pattern, with thickness proportional to the number of samples in the cohort. Alluvia are colored according to the TDP-43 classification prediction of the corresponding sample cohort. The strata (horizontal bars) indicate the overall proportion of samples in which each CE was detected.

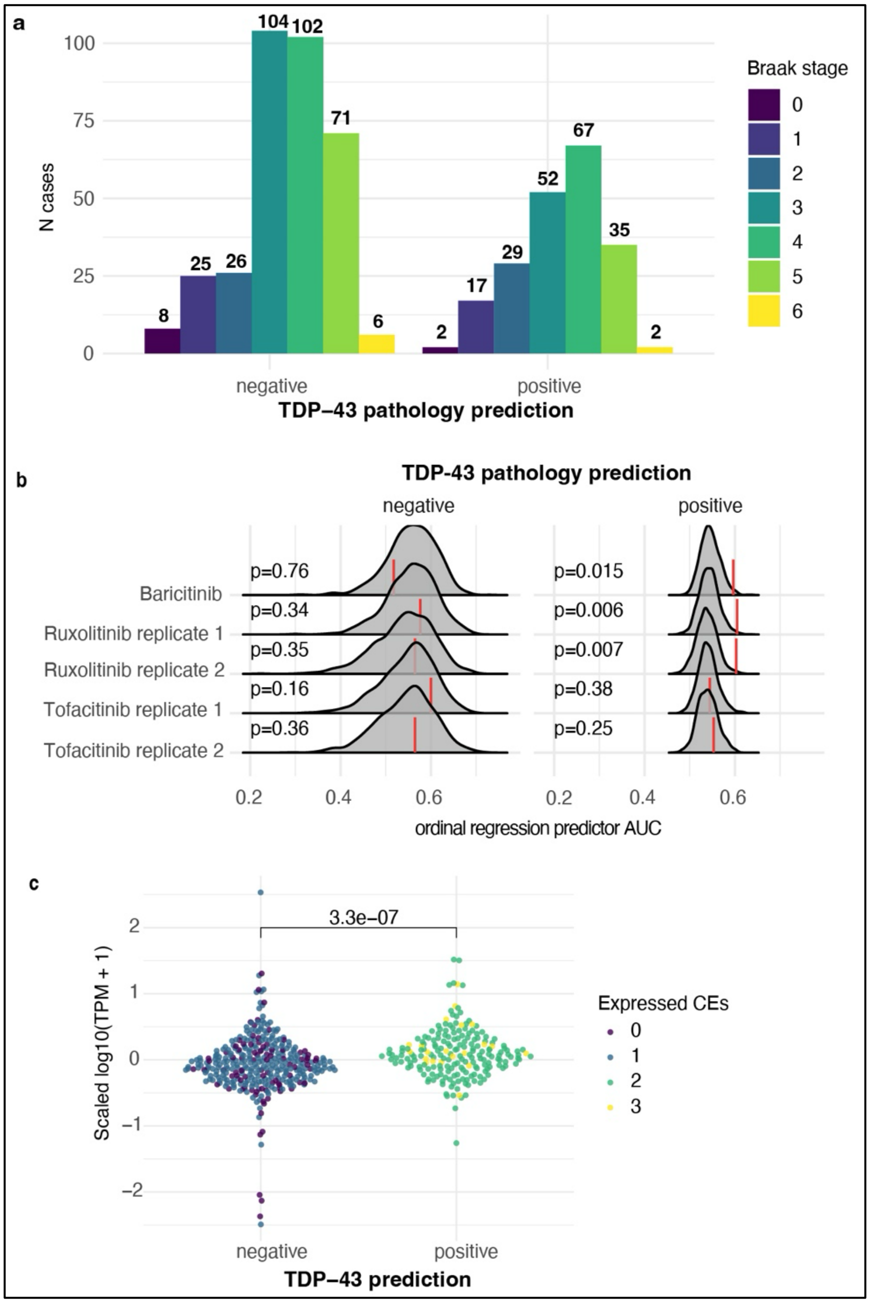

Supplementary Fig. 5: Braak staging of TDP-43 cases and additional data showing TDP-43-associated type-I interferon signaling. a, Comparison of Braak stage distribution of ROSMAP patient data (posterior cingulate cortex) relative to their predicted TDP-43 pathology. **b,** Performance of baricitinib, ruxolitinib and tofacitinib in DRIAD-SP as shown in Fig. 3e, here including additional replicates of ruxolitinib and tofacitinib. Red lines indicate the DRIAD-SP model performance trained on the drug gene sets, whereas the gray shaded regions correspond to the

distribution of model performances based on random gene sets. AUC = area under curve. Empirical one-sided *P* values were computed as the fraction of random background gene sets with higher AUC values than the drug-derived gene set. *P* values were corrected for multiple testing using the Benjamini-Hochberg procedure. **c**, Expression of interferon-stimulated genes (ISGs) in patient cohorts stratified by predicted TDP-43 status. For each sample, gene abundances were transformed using log10(TPM + 1) and standardized to zero mean and unit variance. The average ISG expression per sample was then calculated as the arithmetic mean of the standardized ISG values. Statistical tests: unpaired *t* tests with two tails. $P = 3.3 \times 10^{-7}$.

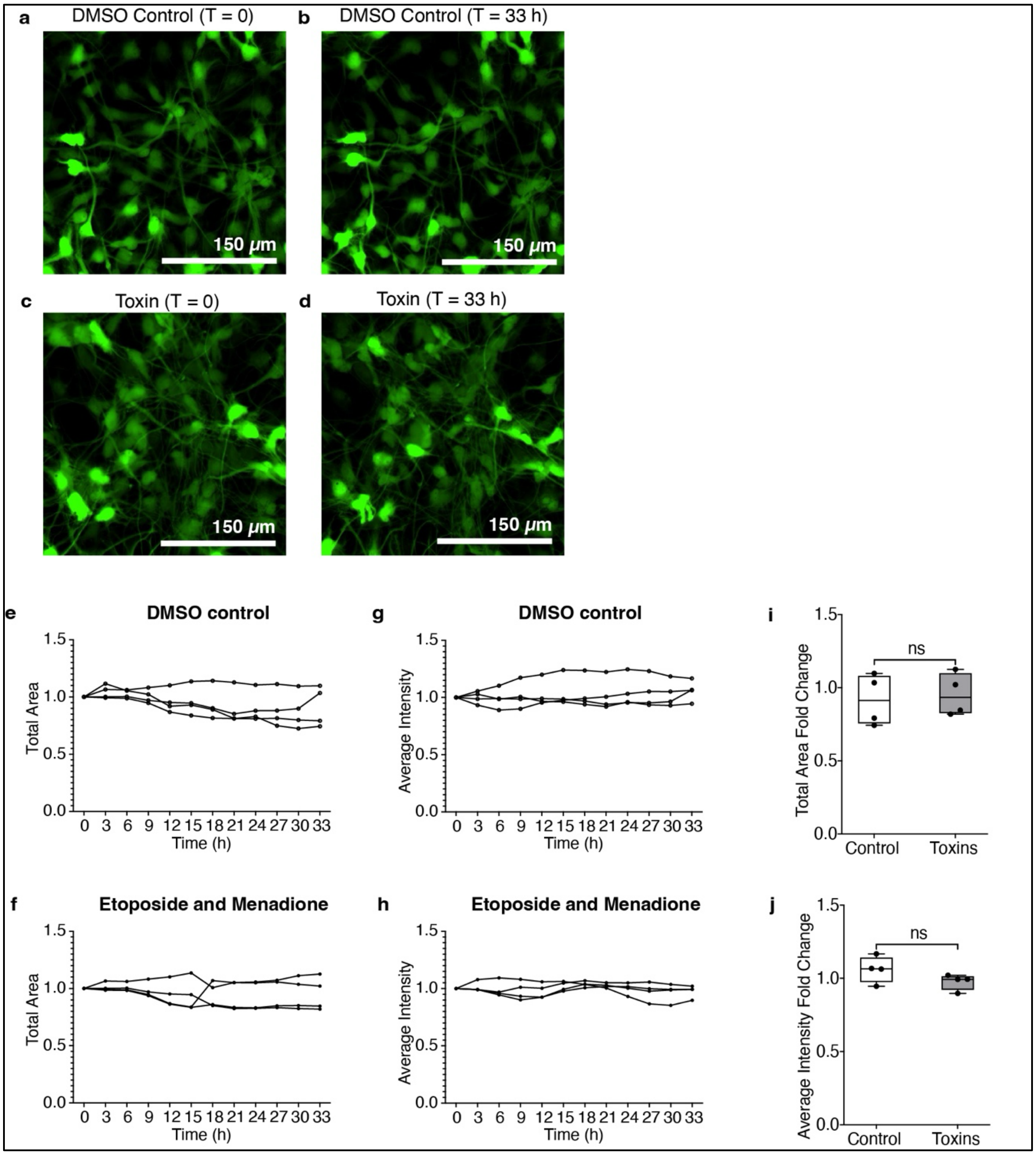

Supplementary Fig. 6: Testing common stressors on differentiated ReN VM cells. To test whether general toxins may also damage differentiated ReN VM cells in a similar way as poly(I:C),

10 μM etoposide, which causes DNA damage, and 10 μM menadione, which induces reactive oxygen species, were added to the differentiated ReN VM cells that express GFP. **a-d,** Control ReN VM cells treated with DMSO vehicle as well as ReN VM cells treated with toxins for 33 hours showed no significant morphological difference. **e-f,** Total cell surface areas were not altered by DMSO or the two toxins over 33 hours. All areas at various time points were normalized to time 0 (n = 4 biological replicates). **g-h,** Average fluorescence intensity stayed constant over 33 hours for both DMSO-treated and toxin-treated ReN VM cell groups. Fluorescence intensity at each time point was normalized to time 0 (n = 4 biological replicates). **i-j,** Total area fold change and average intensity fold change (T = 33 hours / T = 0 hours) were not significantly different ($P > 0.05$) between the DMSO control and toxin-treated groups. Center line, median; box limits, upper and lower quartiles; whiskers, 1.5x interquartile range. Statistical tests: unpaired *t* tests with two tails.

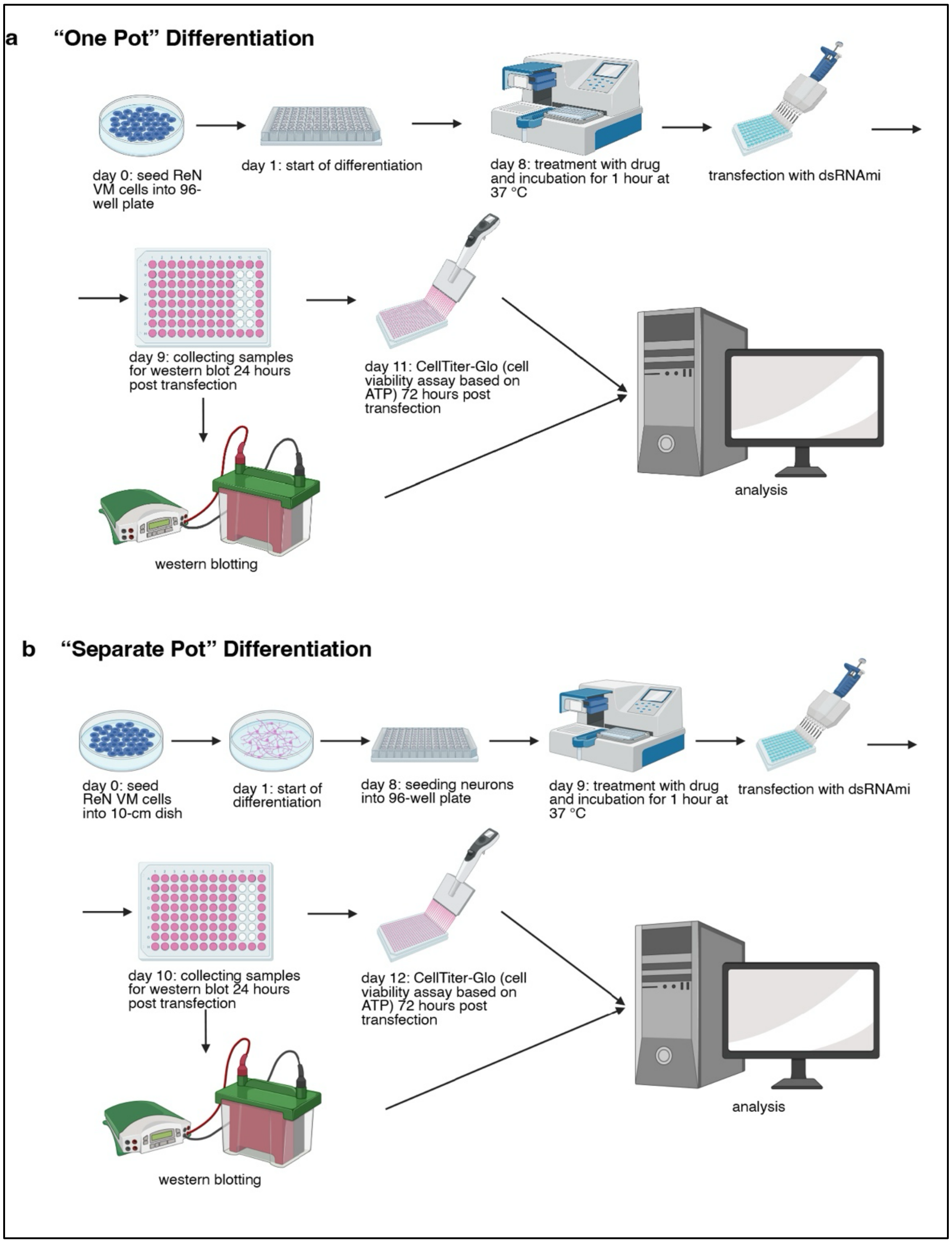

Supplementary Fig. 7: Schematic overview comparing our previously employed drug screening assay workflow[22] and a refined workflow. a, Graphical summary of main steps in drug screening assay according to our previous publication[22] (“One Pot” Differentiation). **b,** Refined workflow including an additional step that comprises the differentiation of cells in a separate dish before being seeded into the final assay plate (“Separate Pot” Differentiation). This ensures less variability in the cell count per well and thus higher reproducibility with lower variance in the results.
Created in BioRender. Konig, L. (2026) https://BioRender.com/vj70z5a

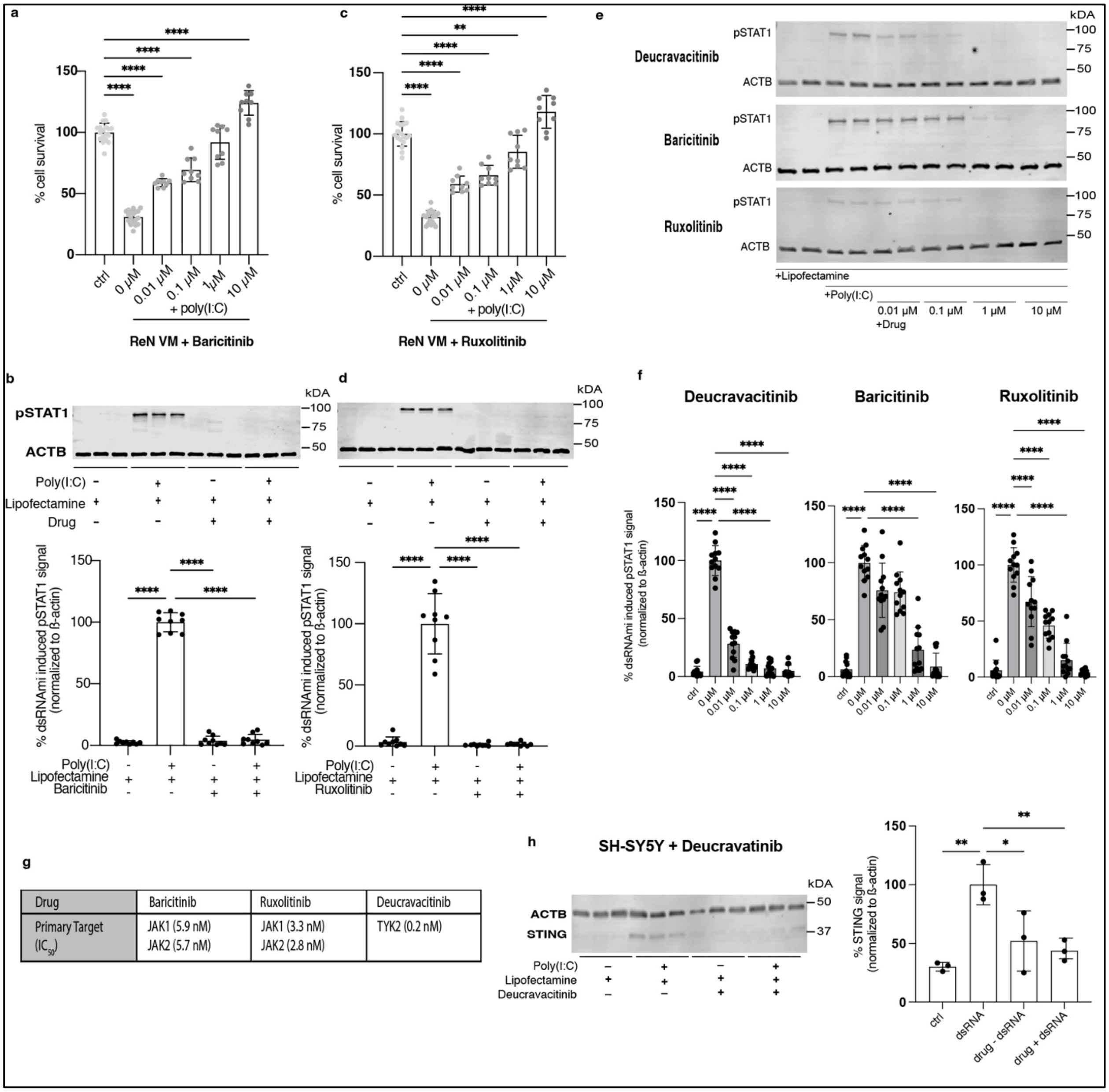

Supplementary Fig. 8: Further validation of baricitinib, ruxolitinib, and deucravacitinib. a-d, Quantification of cell survival of ReN VM-derived neural cells pre-treated with baricitinib (a) or ruxolitinib (c) at different concentrations (baricitinib: 0 µM $P < 0.0001$, n = 18; 0.01 µM $P < 0.0001$, n = 9; 0.1 µM $P < 0.0001$, n = 9; 1 µM $P = 0.0872$, n = 9; 10 µM $P < 0.0001$, n = 9; ruxolitinib: 0 µM $P < 0.0001$, n = 18; 0.01 µM $P < 0.0001$, n = 9; 0.1 µM $P < 0.0001$, n = 9; 1 µM $P = 0.0016$, n = 9; 10 µM $P < 0.0001$, n = 9) and afterwards transfected with poly(I:C) or lipofectamine as a vehicle control (ctrl, n = 18). Baricitinib: EC_{50} = 135.9 nM; ruxolitinib: EC_{50} = 152 nM. Image (b and d, top) and quantification (bottom) of Western blot of $pSTAT1^{Y701}$ in ReN VM-derived neural cells 24 hours after treatment with 10 µM baricitinib (b) or ruxolitinib (d) and transfection with poly(I:C) normalized to the housekeeping protein beta actin (ACTB). Top: n = 3 per condition. Bottom: n = 9 per condition (all $P < 0.0001$). **e-f,** Image (top) and quantification (bottom) of Western blot of $pSTAT1^{Y701}$ in ReN VM-derived neural cells 24 hours after treatment with different doses of deucravacitinib, baricitinib or ruxolitinib, and transfection with poly(I:C)

normalized to the housekeeping protein beta actin (ACTB). Top: n = 2 per condition. Bottom: n = 12 per condition (all *P* < 0.0001). **g,** Primary drug targets of baricitinib, ruxolitinib and deucravacitinib including their IC_{50} values. **h,** Image (left) and quantification (right) of Western blot of STING in SH-SY5Y cells 24 hours after treatment with 1 µM deucravacitinib and transfection with poly(I:C) normalized to the housekeeping protein beta actin (ACTB). N = 3 (ctrl vs. dsRNA *P* = 0.0019, dsRNA vs. drug - dsRNA *P* = 0.0166, dsRNA vs. drug + dsRNA *P* = 0.007).
All replicates in this figure are biological replicates. All replicates are biological replicates. All graph bars represent mean values while the error bars indicate the standard deviation. Statistical tests: ordinary one-way ANOVA with correction for multiple comparison by Dunnett's testing. For western blots, samples derive from the same experiment and gels/blots were processed in parallel. Source Data are provided as a Source Data file.

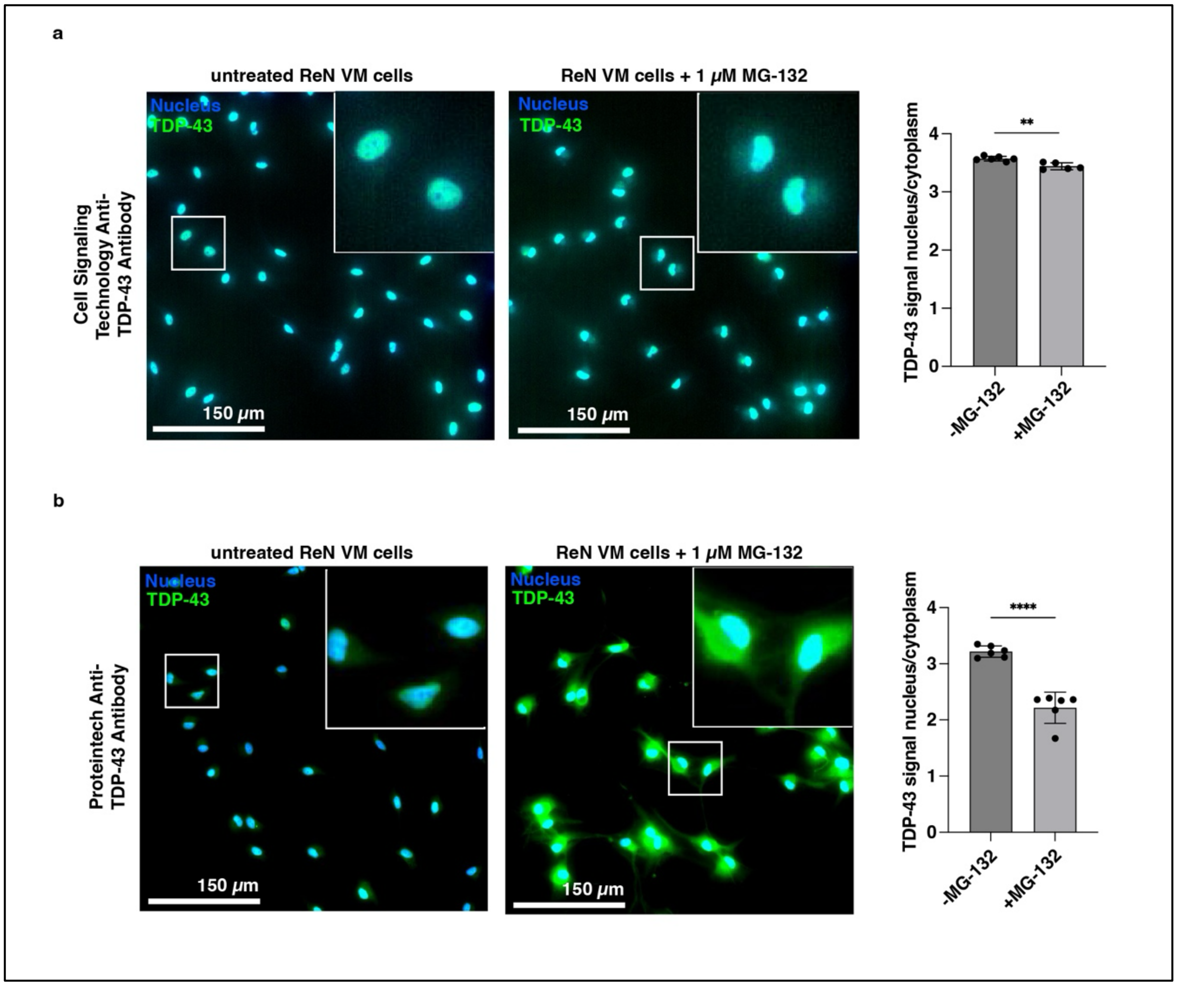

Supplementary Fig. 9: Translocation of TDP-43 after treatment with MG-132 assessed with two different antibodies. Immunofluorescence staining and quantification of fixed ReN VM-derived neural cells comparing the localization of TDP-43 (green) by testing two different antibodies (**a,** Cell Signaling Technology (CST) cat. # 3448; **b,** Proteintech cat. #10782-2-AP) in cells that were left untreated (left) or treated with 1 µM MG-132 for 48 hours (right). Nuclei (blue) were stained with Hoechst. Zoom-ins with 3x magnification. CST: ROUT (Q = 0.1%) and Grubbs (α = 0.001) outlier tests were performed, and one outlier was excluded (n = 6 ctrl, n = 5 +MG-132). *P*

= 0.0018; Proteintech: ROUT (Q = 0.1%) and Grubbs (α = 0.001) outlier tests were performed, and no outlier were excluded (n = 6 both conditions). $P < 0.0001$.
All replicates in this figure are biological replicates. All replicates are biological replicates. All graph bars represent mean values while the error bars indicate the standard deviation. Statistical tests: ordinary one-way ANOVA with correction for multiple comparison by Dunnett's testing. Source Data are provided as a Source Data file.
