# Supplementary material for: TYK2 mediates neuroinflammation in Alzheimer’s disease brains with TDP-43 pathology
Source: Nat Commun. 2026 Mar 14;17:3967. doi: 10.1038/s41467-026-70243-3 (PMC13133158; doi:10.1038/s41467-026-70243-3)
Supplement: Supplementary file 2 — Description of Additional Supplementary Files [file 41467_2026_70243_MOESM2_ESM.pdf]

Description of Additional Supplementary Files

File Name: Supplementary Data 1
Description: RNA-sequencing data analysis looking for upregulated ISGs in Alzheimer's Disease brains

Excel table showing the results of a differential gene expression analysis in Alzheimer's Diseases patients compared to healthy controls (Fig. 2). RNA-sequencing data derived from ROSMAP and MSBB were used to evaluate which interferon-stimulated genes were differentially expressed in selected brain regions relevant in Alzheimer's Disease. The statistical test used to compute fold change and significance was limma (https://pmc.ncbi.nlm.nih.gov/articles/PMC4402510/). Two-sided tests and Benjamini-Hochberg multiple corrections were applied.

File Name: Supplementary Data 2
Description: CRISPR screen data

Excel table showing the results of a CRISPR screen in ReN VM-derived neural cells using the Brunello library (Fig. 4a,b).
