# Supplementary material for: TYK2 mediates neuroinflammation in Alzheimer’s disease brains with TDP-43 pathology
Source: Nat Commun. 2026 Mar 14;17:3967. doi: 10.1038/s41467-026-70243-3 (PMC13133158; doi:10.1038/s41467-026-70243-3)
Supplement: Supplementary file 4 — Reporting Summary [file 41467_2026_70243_MOESM4_ESM.pdf]

nature portfolio

Corresponding author(s): Mark W. Albers

Last updated by author(s): Feb 20, 2026

# Reporting Summary

Nature Portfolio wishes to improve the reproducibility of the work that we publish. This form provides structure for consistency and transparency in reporting. For further information on Nature Portfolio policies, see our Editorial Policies and the Editorial Policy Checklist.

## Statistics

For all statistical analyses, confirm that the following items are present in the figure legend, table legend, main text, or Methods section.

| n/a | Confirmed | |
|---|---|---|
| ☐ | ☒ | The exact sample size (*n*) for each experimental group/condition, given as a discrete number and unit of measurement |
| ☐ | ☒ | A statement on whether measurements were taken from distinct samples or whether the same sample was measured repeatedly |
| ☐ | ☒ | The statistical test(s) used AND whether they are one- or two-sided<br>*Only common tests should be described solely by name; describe more complex techniques in the Methods section.* |
| ☐ | ☒ | A description of all covariates tested |
| ☐ | ☒ | A description of any assumptions or corrections, such as tests of normality and adjustment for multiple comparisons |
| ☐ | ☒ | A full description of the statistical parameters including central tendency (e.g. means) or other basic estimates (e.g. regression coefficient) AND variation (e.g. standard deviation) or associated estimates of uncertainty (e.g. confidence intervals) |
| ☐ | ☒ | For null hypothesis testing, the test statistic (e.g. *F*, *t*, *r*) with confidence intervals, effect sizes, degrees of freedom and *P* value noted<br>*Give P values as exact values whenever suitable.* |
| ☒ | ☐ | For Bayesian analysis, information on the choice of priors and Markov chain Monte Carlo settings |
| ☒ | ☐ | For hierarchical and complex designs, identification of the appropriate level for tests and full reporting of outcomes |
| ☐ | ☒ | Estimates of effect sizes (e.g. Cohen's *d*, Pearson's *r*), indicating how they were calculated |

*Our web collection on statistics for biologists contains articles on many of the points above.*

## Software and code

Policy information about availability of computer code

| | |
|---|---|
| Data collection | For proteomics: Orbitrap Fusion Lumos (Thermo Fisher Scientific) acquisition software was used: Tune (v.3.4), Xcalibur (v.4.5.445.18).<br>Cell imaging: MetaXPress (v.6.5.3.427), GE In-Cell 6000 Analyzer software (v.7.2)<br>Tissue imaging: Imager5 software (RareCyte Inc.)<br>Western blotting: Image Studio Lite (v.5.2.5)<br>Cell viability assessment: Gen5 (v.3.2)<br>Electrochemiluminescence biomarker assessment: Meso Scale Discovery WorkBench (v.4.0) |
| Data analysis | For differential gene expression analysis of different brain regions: R version 4.2.1, limma 3.52.4, ggplot2 3.4.0 functions in R as well as the fgsea R package (v.1.32.4)<br>For gene enrichment analysis: R (v.4.4.2), enrichR (v.3.4), fgsea R packages (v.1.32.4), ComplexHeatmap (v.2.22.0)<br>DRIAD-SP: R (v.4.2.1); https://github.com/labsyspharm/ad-personalized; https://github.com/labsyspharm/driad (v.0.2.5); https://github.com/labsyspharm/ordinalRidge (v.0.1); dplyr (v.1.1.2); ggplot2 (v.3.4.2); ggbeeswarm (v.0.7.2), data.table (v.1.14.8), Salmon (v.1.9.0), tximport (v.1.34.0), msigdbr (v.24.1)<br>For proteomics: JMP Pro (v.16), Graphpad Prism (v.9.3.1), monocle (v.1.1), Sequest (v.28 rev.12).<br>For quantification of immunofluorescent images: custom Fiji/ImageJ-based plugin (National Institutes of Health, version 1.53c), R-Studio software (Version 4.3.3)<br>For genome-wide association test: REGENIE v.2.2.1, BOLT-LMM (v.2.4.1)<br>Western blot quantification: Image Studio Lite (v.5.2.5), Graphpad Prism (v.9.3.1)<br>Cell viability assessment: Graphpad Prism (v.9.3.1)<br>Electrochemiluminescence biomarker assessment: Graphpad Prism (v.9.3.1) |

Genome-wide association study: REGENIE (v.2.2.1 )

For manuscripts utilizing custom algorithms or software that are central to the research but not yet described in published literature, software must be made available to editors and reviewers. We strongly encourage code deposition in a community repository (e.g. GitHub). See the Nature Portfolio guidelines for submitting code & software for further information.

## Data

Policy information about availability of data

All manuscripts must include a data availability statement. This statement should provide the following information, where applicable:

- Accession codes, unique identifiers, or web links for publicly available datasets
- A description of any restrictions on data availability
- For clinical datasets or third party data, please ensure that the statement adheres to our policy

For all compounds with an HMS LINCS ID, the compound information, including the vendors they were purchased from, can be found on the HMS LINCS website (https://lincs.hms.harvard.edu/). The proteomics raw data and search results generated in this study have been deposited in the ProteomeXchange Consortium via the PRIDE partner repository under accession code PXD043641 [https://proteomecentral.proteomexchange.org/cgi/GetDataset?ID=PXD043641].
A reporting summary for this article is available as Supplementary Information file. Source Data are provided with this paper. The source data underlying Fig. 4f,g, Fig. 5b,d,f, 6b, and Supplementary Fig. 8b,d,e,h are provided in the Source Data file.
The bulk RNA-sequencing data used in this study are available in the ROSMAP34 and MSBB35 databases obtained from the AMP-AD Knowledge Portal under the accession code syn2580853 on Synapse [doi:10.7303/syn2580853].

## Research involving human participants, their data, or biological material

Policy information about studies with human participants or human data. See also policy information about sex, gender (identity/presentation), and sexual orientation and race, ethnicity and racism.

| | |
|---|---|
| Reporting on sex and gender | See Supplementary Table 1 - Sex was self-reported by participants. While an effort to achieve balanced representation was made, sex was not directly considered in the study design and data analysis to ensure that findings apply to any sex. Our cell lines are established models. Sex-matched lines are not available commercially.<br>Informed consent, including consent to publish three or more indirect identifiers, was given by all participants. Participants were not compensated for their contribution to this study.<br>Overall, samples from ten female and nine male participants were included in this study. |
| Reporting on race, ethnicity, or other socially relevant groupings | Race was self-reported by participants. Neither race nor ethnicity was considered in the study design or data analysis.<br>Informed consent, including consent to publish three or more indirect identifiers, was given by all participants. As requested on the author checklist, certain identifiers, including race, was removed from Supplementary Table 1. Participants were not compensated for their contribution to this study. |
| Population characteristics | See Supplementary Table 1 |
| Recruitment | Participants were recruited from the MADRC brain bank. |
| Ethics oversight | MGB IRB protocol 2006P002104 |

Note that full information on the approval of the study protocol must also be provided in the manuscript.

# Field-specific reporting

Please select the one below that is the best fit for your research. If you are not sure, read the appropriate sections before making your selection.

☒ Life sciences ☐ Behavioural & social sciences ☐ Ecological, evolutionary & environmental sciences

For a reference copy of the document with all sections, see nature.com/documents/nr-reporting-summary-flat.pdf

# Life sciences study design

All studies must disclose on these points even when the disclosure is negative.

| | |
|---|---|
| Sample size | Sample sizes were chosen in a way that ensures at least three (exception: Figure 4g, explanation see below) and up to 24 biological replicates per condition which was sufficient as the replicates were enough to achieve robust and significant results. No statistical method was used to predetermine sample size. |
| Data exclusions | No data was excluded from the study. Only outliers were excluded in a few cases that are transparently indicated in the respective figure legend. |
| Replication | We used at least three biological replicates at all times except for Figure 4g which was a proof-of-concept and did not require statistics. All attempts at replication were successful. |
| Randomization | Randomization was not relevant to our study as all grouping was done based on phenotype, differences in treatments, and/or diagnosis. |
| Blinding | Blinding was implemented in our study during the examination of human brain histological sections. This was the only experiment that |

required blinding in any way.

# Reporting for specific materials, systems and methods

We require information from authors about some types of materials, experimental systems and methods used in many studies. Here, indicate whether each material, system or method listed is relevant to your study. If you are not sure if a list item applies to your research, read the appropriate section before selecting a response.

## Materials & experimental systems

| n/a | Involved in the study |
|---|---|
| ☐ | ☒ Antibodies |
| ☐ | ☒ Eukaryotic cell lines |
| ☒ | ☐ Palaeontology and archaeology |
| ☒ | ☐ Animals and other organisms |
| ☒ | ☐ Clinical data |
| ☒ | ☐ Dual use research of concern |
| ☒ | ☐ Plants |

## Methods

| n/a | Involved in the study |
|---|---|
| ☒ | ☐ ChIP-seq |
| ☒ | ☐ Flow cytometry |
| ☒ | ☐ MRI-based neuroimaging |

## Antibodies

Antibodies used

Primaries:
J2 for dsRNA (1:200, cat. # 76651, Cell Signaling Technology, MA, USA);
K1 anti-dsRNA (1:250, cat. # 10020200, Scicons, Susteren, The Netherlands);
anti-TDP-43 (1:200, at. #ab193842, abcam, MA, USA) for human samples, (1:100, cat. # 3448, Cell Signaling Technology) and (1:500, cat. #10782-2-AP, Proteintech, IL, USA) for cell stainings;
pTDP-43 S409/410 (1:200, cat. # CAC-TIP-PTD-P02, Cosmo Bio, CA, USA);
tau (1:100, cat. # NBP2-25162, Novus Biologicals, CO, USA);
Aß (1:100, cat. # CST-D54D2, Cell Signaling Technology);
pPKR (1:100, cat. # bs-3336R-Cy3, Bioss Antibodies, MA, USA);
pSTAT1 Y701 (1:1000, cat. # 9167S, Cell Signaling Technology);
beta actin ACTB (1:1000, cat. # 3700, Cell Signaling Technology);
anti-TUJ1 (1:250, cat. # TUJ, Aves Lab, CA, USA);
TYK2 (1:1000, cat. #ab303500, abcam);
STING (1:1000, cat. #13647, Cell Signaling Technology).

Secondaries:
anti-rabbit Alexa Flour 555 (1:1000, cat. # A-31572, Thermo Fisher, MA, USA);
anti-mouse Alexa Flour 647 (1:1000, cat. # A-21237, Thermo Fisher);
anti-chicken Alexa Fluor 488 (1:500, cat. # 703-545-155, Jackson ImmunoResearch, PA, USA);
anti-rabbit Alexa Fluor 568 (1:500 for iPSCs, 1:1000 for ReN VM, cat. # A10042, Thermo Fisher);
anti-mouse Alexa Fluor 647 (1:500 for iPSCs, 1:1000 for ReN VM, cat. # A31571, Thermo Fisher);
anti-rabbit Alexa Fluor 488 (1:1000, cat. # A21206, Thermo Fisher);
IRDye 800CW (1:2500, cat. # 926-32211, LI-COR, NE, USA);
IRDye 680RD (1:2500, cat. #926-68070, LI-COR).

Validation

All antibodies are commercially available and the validation data is available on the website of the manufacturer.

## Eukaryotic cell lines

Policy information about cell lines and Sex and Gender in Research

Cell line source(s)

ReNcell VM Cell Line (cat. # SCC008, Millipore) and ReNcell CX Cell Line (cat. # SCC007, Millipore); SH-SY5Y Neuroblastoma cells (cat. # CRL-2266, ATCC); a recently established iPSC line that harbors a pathogenic TDP-43 mutation in TDP-43(+/G298S) in the FA0000011 genetic background (PMID: 31346219, 38478117)

Authentication

The cell line was not authenticated.

Mycoplasma contamination

Cells tested negative for mycoplasma contamination during quality assurance by manufacturer.

Commonly misidentified lines
(See ICLAC register)

None.
