# Supplementary material for: TYK2 mediates neuroinflammation in Alzheimer’s disease brains with TDP-43 pathology
Source: Nat Commun. 2026 Mar 14;17:3967. doi: 10.1038/s41467-026-70243-3 (PMC13133158; doi:10.1038/s41467-026-70243-3)

Figure 1c: Quantification of Immunofluorescent staining of wildtype (isoTDP-43+/+) diffe
transfected with poly(I:C)

Explanation of each variable below, plotted values in red

	treatment	IntDenNucl_well	TUJ_well	IntDenPerCell
1	lipo	3840.49	10	384.049
2	lipo	13293.11	7	1899.015714
3	lipo	2180.62	4	545.155
4	lipo	5409.3	9	601.0333333
5	lipo	6182.35	9	686.9277778
6	lipo	13546.96	5	2709.392
7	lipo	3367.98	9	374.22
8	lipo	11322.59	12	943.5491667
9	lipo	53.7	3	17.9
10	lipo	693.63	11	63.05727273
11	lipo	0	4	0
12	lipo	5952.73	2	2976.365
13	lipo	7238.35	3	2412.783333
14	lipo	0	11	0
15	lipo	2430.87	3	810.29
16	lipo	970.49	9	107.8322222
17	lipo	1950.75	9	216.75
18	lipo	839.46	10	83.946
19	lipo	2473.82	6	412.3033333
20	lipo	5577.99	2	2788.995
21	lipo	0	7	0
22	lipo	17499.5	7	2499.928571
23	lipo	2202.47	13	169.4207692
24	lipo	10216.79	2	5108.395
25	lipo	739.18	2	369.59
26	lipo	12614.33	12	1051.194167
27	lipo	243.77	14	17.41214286
28	polyIC	50583.12	9	5620.346667
29	polyIC	142221.12	3	47407.04
30	polyIC	50214.41	2	25107.205
31	polyIC	442188.15	6	73698.025
32	polyIC	304164.23	9	33796.02556
33	polyIC	61601.13	4	15400.2825
34	polyIC	186028.52	5	37205.704
35	polyIC	471676.67	5	94335.334
36	polyIC	579087.4	3	193029.1333
37	polyIC	329186.98	6	54864.49667
38	polyIC	0	2	0
39	polyIC	193982.55	5	38796.51
40	polyIC	621051.88	10	62105.188

41	polyIC	302390.61	4	75597.6525
42	polyIC	2714.58	2	1357.29
43	polyIC	72394.77	4	18098.6925
44	polyIC	332587.64	8	41573.455
45	polyIC	52.45	2	26.225
46	polyIC	7873.55	14	562.3964286
47	polyIC	255531.93	9	28392.43667
48	polyIC	505392.11	8	63174.01375
49	polyIC	356484.85	14	25463.20357
50	polyIC	122536.31	8	15317.03875
51	polyIC	29426.39	11	2675.126364

Legend

treatment	either untreated (lipo) or polyIC
IntDenNucl_well	The **average dsRNA integrated intensity per well**, calculated by averaging [illegible]
TUJ_well	The average TUJ signal per well, calculated by averaging tuj_site across site[illegible]
IntDenPerCell	The dsRNA signal normalized to cell content: IntDenNucl_well / TUJ_well
norm	A replicate-specific normalization constant, intended to represent the me[illegible]
norm_effect	The final normalized dsRNA metric used for plotting and statistics: IntDenP[illegible]

rentiated iPSC-derived NGN2 cortical-like neurons

Figure 1d: Quantification

norm	norm_effect (plotted values)
671	0.572353204
671	2.830127741
671	0.812451565
671	0.895727769
671	1.023737374
671	4.037842027
671	0.557704918
671	1.406183557
671	0.026676602
671	0.093975071
671	0
671	4.43571535
671	3.595802285
671	0
671	1.207585693
671	0.160703759
671	0.323025335
671	0.125105812
671	0.614461003
671	4.15647541
671	0
671	3.725675963
671	0.252489969
671	7.613107303
671	0.550804769
671	1.566608296
671	0.025949542
671	8.376075509
671	70.65132638
671	37.41759314
671	109.8331222
671	50.36665508
671	22.95124069
671	55.44814307
671	140.5891714
671	287.6738202
671	81.76527074
671	0
671	57.81894188
671	92.55616692

	iPSC line
1	TDPMut
2	TDPMut
3	TDPMut
4	TDPMut
5	TDPMut
6	TDPMut
7	TDPMut
8	TDPMut
9	TDPMut
10	TDPMut
11	TDPMut
12	TDPMut
13	TDPWT
14	TDPWT
15	TDPWT
16	TDPWT
17	TDPWT
18	TDPWT
19	TDPWT
20	TDPWT
21	TDPWT
22	TDPWT
23	TDPWT

671	112.6641617
671	2.022786885
671	26.9727161
671	61.95745902
671	0.039083458
671	0.838146689
671	42.31361649
671	94.14905179
671	37.94814243
671	22.82718145
671	3.986775505

the raw dsRNA signal across all sites/objects in that well
:s/objects in that well. Used as a proxy for cell/neuronal content.

an control (lipo) IntDenPerCell value for that replicate (manually entered in this script).
'erCell / norm

of Immunofluorescent staining of wildtype (isoTDP-43+/+) differentiated iPSC-derived NGN2 cortical-like neurons transfected with poly(I:C)

replicate number	IntDenNucl_well	TUJ_well	IntDenPerCe	norm	norm_effect (plotted value)
2	857111.9053	4.4	194798.16	276829	0.703676856
2	1766519.383	3.88888889	454247.841	276829	1.640896877
2	880942.1582	3.90909091	225357.296	276829	0.814066793
2	1357503.994	4	339375.999	276829	1.225940918
2	967971.1813	4.26666667	226868.246	276829	0.819524853
2	2927543.43	4.33333333	675586.945	276829	2.4404486
3	100688.178	3.36	29966.7196	12811	2.339139774
3	95027.56364	3.40909091	27874.752	12811	2.175845133
1	127935.2563	7.5	17058.0342	16557	1.030261168
1	451783.4371	6.76190476	66813.0435	16557	4.035335116
1	260735.7075	4.16666667	62576.5698	16557	3.779463055
1	64072.51722	4	16018.1293	16557	0.967453603
2	803965.744	3.8	211569.933	276829	0.764262171
2	411429.8177	3.84615385	106971.753	276829	0.386418159
2	1349057.002	3.66666667	367924.637	276829	1.329068258
2	815579.851	4.2	194185.679	276829	0.701464365
3	1910.042353	2.88235294	662.667755	12811	0.051726466
2	1921345.482	3.33333333	576403.645	276829	2.082164963
2	837138.8695	4.10526316	203918.443	276829	0.736622401
3	66070.24588	2.64705882	24959.8707	12811	1.948315562
1	104550.9623	7.12903226	14665.5196	16557	0.885759473
1	92434.356	7.13333333	12958.0873	16557	0.782634976
1	144445.1934	6.55172414	22046.8979	16557	1.331575645

Figure 4d: Detected CellTiter-Glo luminescence signal
antibody after transfe

plotted values in red

biological replicate	ctrl	no αIFNAR2 antibody
1	37954	14306
2	36383	14819
3	36915	17569
4	35124	16185
average	36594	15719.75

normalizing signal to average ctrl *100:

Percentage cell survival

biological replicate	ctrl	no αIFNAR2 antibody
1	103.716456	39.09384052
2	99.4234027	40.49570968
3	100.877193	48.01060283
4	95.982948	44.22856206
average	100	42.95717877

- Cell survival of ReN VM-derived neural cells treated with anti-IFNAR2
ction with poly(I:C) or lipofectamine

1:50 αIFNAR2 antibody	1:25 αIFNAR2 antibody	1:10 αIFNAR2 antibody
20955	23001	25207
28121	28824	27313
25160	24636	30133
20955	23001	25207

1:50 αIFNAR2 antibody	1:25 αIFNAR2 antibody	1:10 αIFNAR2 antibody
57.26348582	62.85456632	68.88287697
76.84593103	78.76701099	74.63791878
68.75444062	67.32251189	82.34410013
67.62128582	69.64802973	75.28829863

Figure 4e: Detected CellTiter-Glo luminescence signal - C
derived neural cells treated with different doses c

biological replicate	0 U/ml	10 U/ml	100 U/ml
1	32987	32592	32999
2	39618	30703	33875
3	37839	29834	42805
average	36814.6667	31043	36559.6667

normalizing signal to average ctrl *100:

Percentage cell survival

biological replicate	0 U/ml	10 U/ml	100 U/ml
1	89.6028757	88.5299337	89.6354714
2	107.614719	83.3988266	92.0149578
3	102.782406	81.0383543	116.271595
average	100	84.3223715	99.3073413

Cell survival of ReN VM-
of interferon-a

1000 U/ml	10000 U/ml
31753	36199
29719	29902
33173	31852
31548.3333	32651

1000 U/ml	10000 U/ml
86.2509507	98.3276593
80.7259788	81.2230633
90.1081091	86.5198653
85.6950129	88.6901959

Figure 4f: Image and quantification of Western blot of TYK2 in undifferentiated control and TYK2 (KD) ReN VM-derived neural cells normalized to the housekeeping protein beta actin (A

anti-TYK2

plotted values in red, black dotted rectangules correspond to shown images in manuscript, red d

shown in figure:

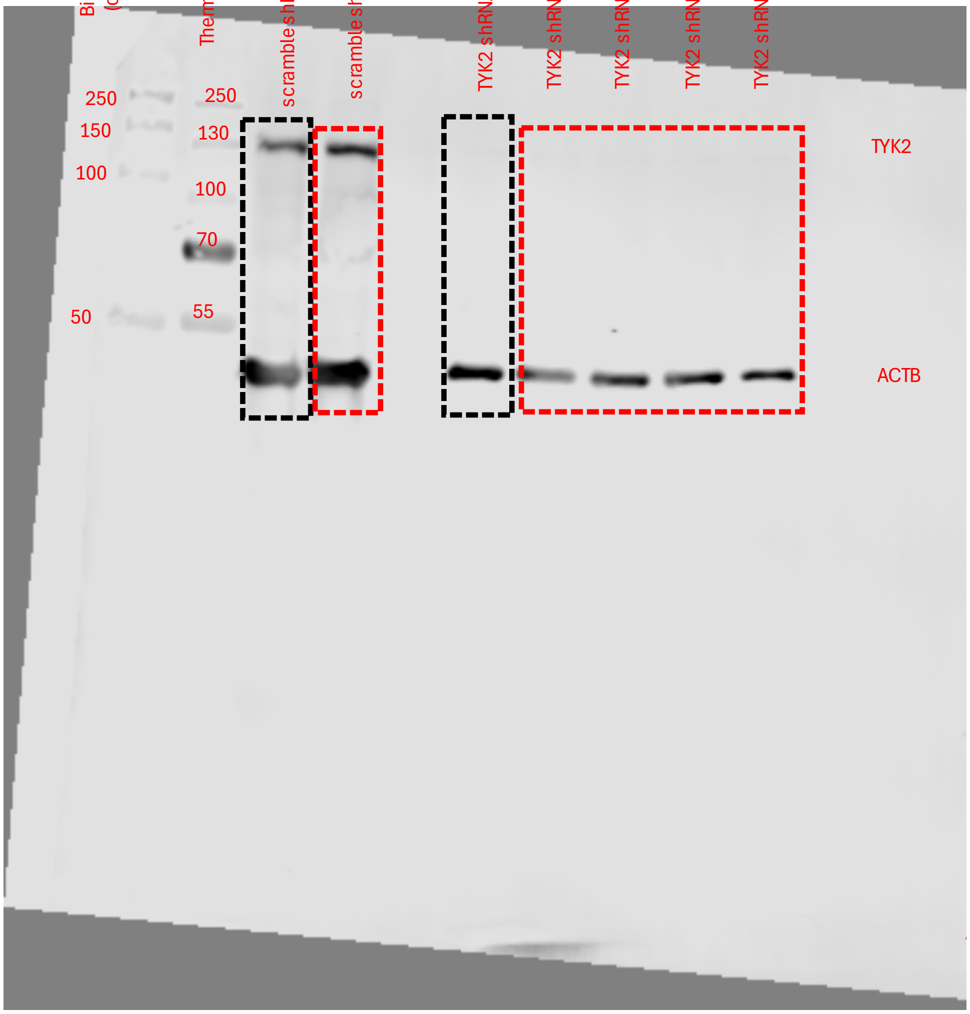

additional blots for quantification (not shown but included in quantification):

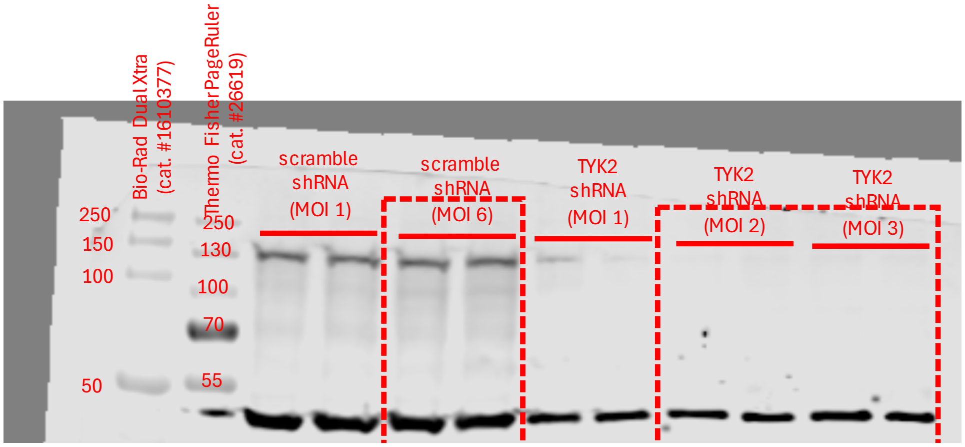

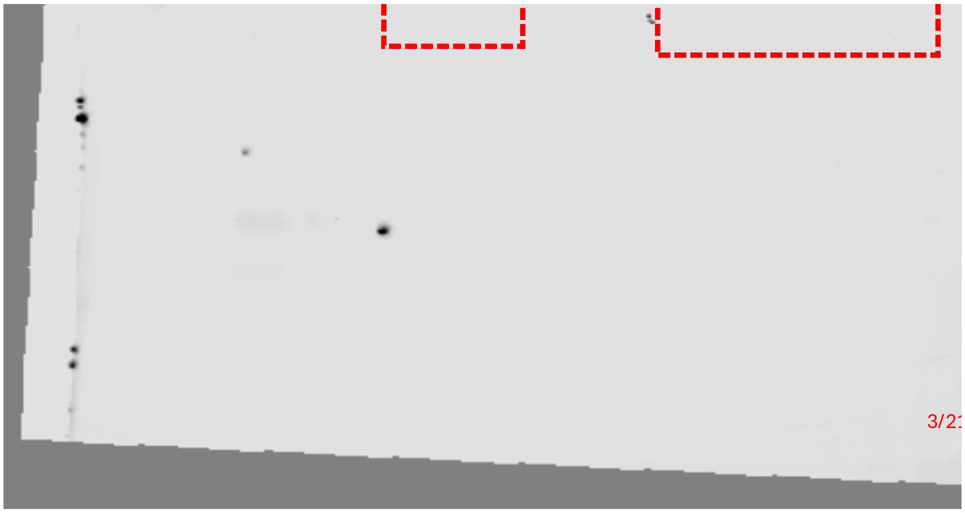

knockdown
CTB)

otted rectangues correspond to excluded samples

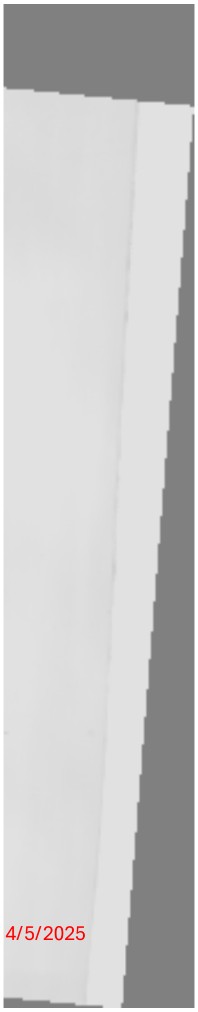

Quantification of bands with ImageStudioLite

	ctrl MOI 1 replicate 1
TYK2	2206.282552
actin	51961.59505
TYK2 signal normalized to actin	0.04245987
normalized to ctrl MOI 1 *100	100

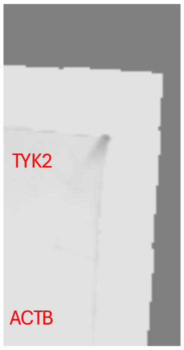

	ctrl MOI 1 replicate 2
TYK2	1873.822591
actin	58732.68913
TYK2 signal normalized to actin	0.031904253
normalized to ctrl MOI 1 *100	95.35003716

1/2025

TYK2 KD MOI 1 replicate 1
71.99984809
44369.20964
0.001622744
3.821828821

ctrl MOI 1 replicate 3	TYK2 KD MOI 1 replicate 2	TYK2 KD MOI 1 replicate 3
1888.545573	268.9980469	45.0406901
53933.75684	32914.36882	34301.87858
0.035016021	0.008172663	0.001313068
104.6499628	24.42507339	3.92427481

Figure 4g: Image and quantification of Western blot of TYK2 in differentiated control and TYK2 [illegible]
(KD) ReN VM-derived neural cells normalized to the housekeeping protein beta actin (A[illegible]

anti-TYK2

plotted values in red, black dotted rectangules correspond to shown images in manuscript, red [illegible]

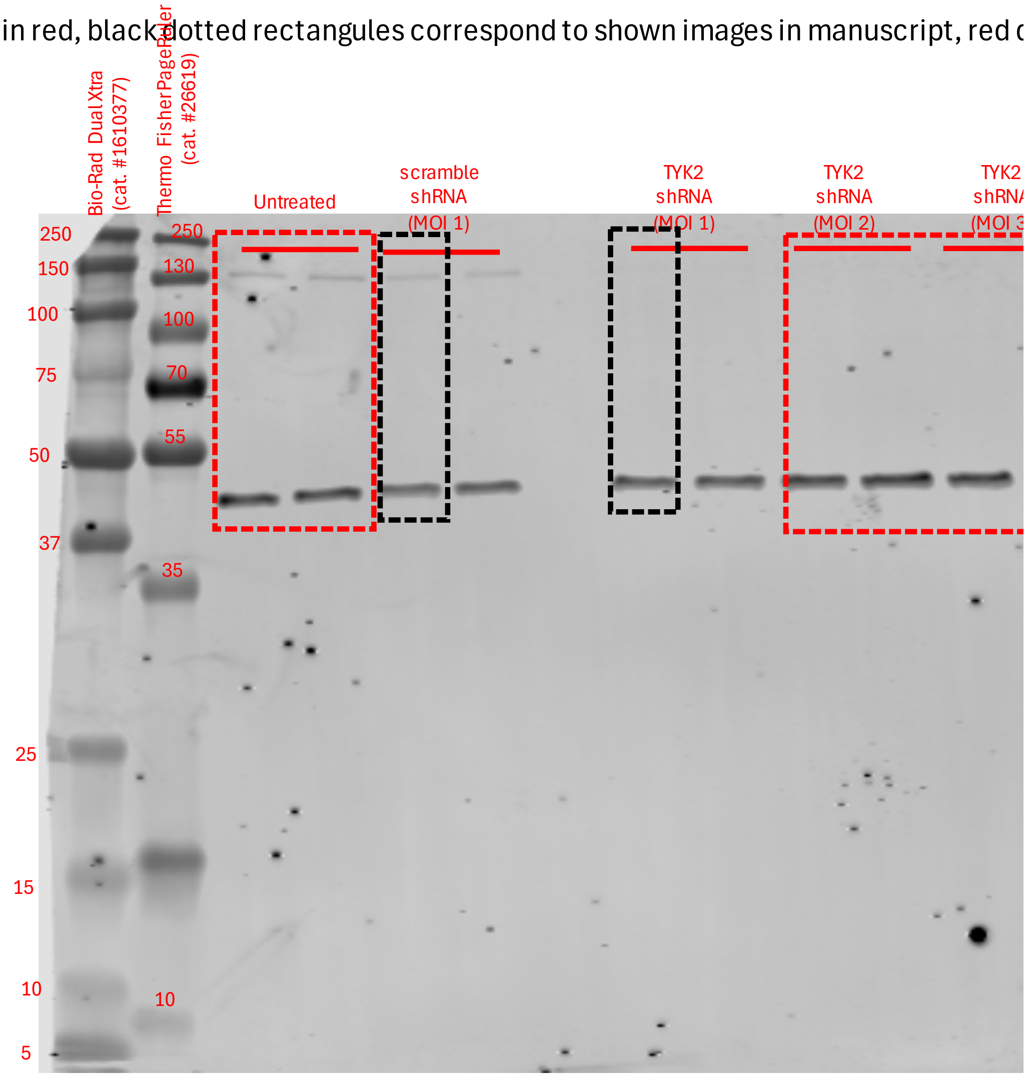

knockdown
CTB)

dotted rectangues correspond to excluded samples

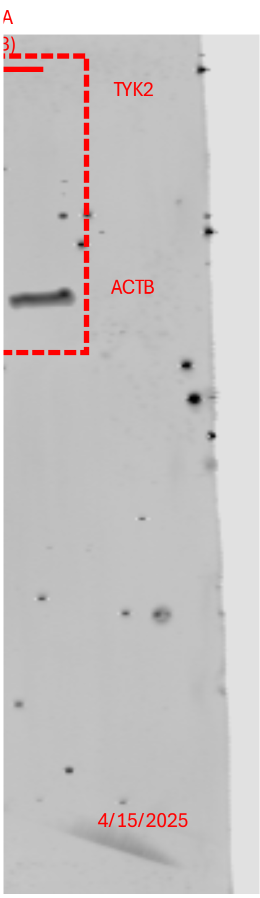

Quantification of bands with ImageStudioLite

	ctrl MOI 1 replicate 1
TYK2	41.04199219
actin	2395.790365
TYK2 signal normalized to actin	0.017130878
normalized to ctrl MOI 1 *100	106.0682669

ctrl MOI 1 replicate 2	TYK2 KD MOI 1 replicate 1	TYK2 KD MOI 1 replicate 2
43.03710938	8.807617188	6.417317708
2836.851563	3013.758464	3291.821615
0.01517073	0.002922469	0.001949473
93.93173307	18.09488556	12.07044

Figure 4h: Detected CellTiter-Glo luminescence signal - Cell survival of differe

Experiment 1

		ReN VM TYK2 KD	
	Biological replicate	polyIC	lipofectamine
	1	5008	6550
	2	5114	6535
	3	4829	5771
	4	5151	6217
	5	5170	6339
	6	5144	5842
	average	5069.33333	6209
Percentage cell survival	normalizing signal to average lipo ctrl *100:	80.6571106	105.492028
		82.3643099	105.250443
		77.7741987	92.9457239
		82.960219	100.128845
		83.2662264	102.093735
		82.8474795	94.0892253
	average	81.644924	100

Experiment 2

		ReN VM TYK2 KD	
	Biological replicate	polyIC	lipofectamine
	7	5257	6013
	8	5463	6133
	9	5428	6274
	10	4920	6062
	11	5217	5855
	12	4943	6264
	average	5204.66667	6100.16667
Percentage cell survival	normalizing signal to average lipo ctrl *100:	86.1779733	98.5710773
		89.5549302	100.538237
		88.9811754	102.849649
		80.6535341	99.374334
		85.5222535	95.9809841
		81.0305729	102.685719
	average	85.3200732	100

Experiment 3

	ReN VM TYK2 KD	
Biological replicate	polyIC	lipofectamine

	13	3935	4336
	14	3807	4433
	15	3742	4409
	16	3671	4445
	17	3926	4484
	18	4018	4308
average		3849.83333	4402.5
Percentage cell survival normalizing signal to average lipo ctrl *100:		89.3810335	98.4894946
		86.4735945	100.692788
		84.9971607	100.147643
		83.3844407	100.965361
		89.1766042	101.851221
		91.266326	97.8534923
average		87.4465266	100

Experiment 4

		ReN VM TYK2 KD	
Biological replicate		polyIC	lipofectamine
	19	3731	3843
	20	3552	3971
	21	3490	3990
	22	3512	4084
	23	3869	3969
	24	3727	4197
average		3646.83333	4009
Percentage cell survival normalizing signal to average lipo ctrl *100:		93.0656024	95.8593165
		88.6006485	99.0521327
		87.0541282	99.5260664
		87.6028935	101.870791
		96.5078573	99.0022449
		92.9658269	104.689449
average		90.9661595	100

entiated control and TYK2 KD ReN VM-derived neural cells transfected with poly(I:C) or lipofectami

e only

		ReN VM with
	Biological replicate	lipo
	1	5466
	2	5385
	3	5514
	4	5389
	5	5614
	6	5656
	7	5506
	8	5456
	9	5379
	10	5416
	11	5746
	12	5648
	average	5514.583
Percentage cell survival	normalizing signal to average lipo ctrl *100:	99.119
		97.65017
		99.98942
		97.7227
e only		101.8028
		102.5644
		99.84435
		98.93767
		97.54137
		98.21232
		104.1964
		102.4193
	average	99.9999917

e only

e only

ne

scramble RNA
poly IC
2081
1885
1764
1986
2243
2086
1522
1724
1229
1276
1384
1430
1717.5
37.73631
34.18209
31.98791
36.0136
40.67397
37.82697
27.59955
31.26256
22.28636
23.13865
25.09709
25.93124
31.1446917

Figure 5a: Detected CellTiter-Glo luminescence signal - Cell survival of ReN VM-derived neural cel
lipofectamine (ctrl)

plotted values in red

experiment 1

biological replicate	ctrl	0 µM	1 pM	10 pM
1	3561	1991	2465	2411
2	2807	2110	2069	2406
3	3490	2170	2123	2172
4	2925	2255	2074	2243
5	3117	1798	1905	2347
6	3309	1887	2305	2490
average	3201.5	2035.16667	2156.83333	2344.83333

normalizing signal to average ctrl *100:

Percentage cell survival

biological replicate	ctrl	0 µM	1 pM	10 pM
1	111.229111	62.1895986	76.9951585	75.3084492
2	87.6776511	65.9066063	64.6259566	75.1522724
3	109.011401	67.7807278	66.3126659	67.8431985
4	91.3634234	70.4357333	64.7821334	70.0609089
5	97.3606122	56.1611744	59.5033578	73.3093862
6	103.357801	58.9411213	71.9975012	77.7760425
average	100	63.5691603	67.3694622	73.2417096

experiment 2

biological replicate	ctrl	0 µM	1 pM	10 pM
7	3810	2343	2435	2157
8	3359	2349	2227	1962
9	3283	1914	1822	1800
10	3271	2091	2095	1891
11	3226	2004	1997	2181
12	2075	1421	1656	1771
average	3170.66667	2020.33333	2038.66667	1960.33333

normalizing signal to average ctrl *100:

Percentage cell survival

biological replicate	ctrl	0 µM	1 pM	10 pM
7	120.164003	73.8961312	76.7977292	68.029857
8	105.939865	74.0853659	70.2375946	61.8797309

	9	103.542893	60.3658537	57.4642557	56.7703953
	10	103.164424	65.9482759	66.0744323	59.6404542
	11	101.745164	63.2043734	62.9835997	68.7867956
	12	65.4436501	44.8170732	52.2287637	55.8557611
average		100	63.7195122	64.2977292	61.8271657

experiment 3

biological replicate	ctrl	0 µM	1 pM	10 pM
13	3339	2044	2386	2196
14	3523	2190	2511	2447
15	3435	2083	2443	2436
16	3112	2195	2237	2402
17	3027	1825	2239	1844
18	2465	1133	1958	1976
average	3150.16667	1911.66667	2295.66667	2216.83333

normalizing signal to average ctrl *100:

Percentage cell survival

biological replicate	ctrl	0 µM	1 pM	10 pM
13	105.994392	64.8854558	75.7420242	69.7105973
14	111.835353	69.5201312	79.7100683	77.6784297
15	109.04185	66.1234855	77.5514523	77.3292418
16	98.7884239	69.678853	71.0121158	76.2499339
17	96.090154	57.9334427	71.0756045	58.5365854
18	78.2498281	35.966351	62.1554415	62.7268398
average	100	60.6846199	72.8744511	70.371938

experiment 4

biological replicate	ctrl	0 µM	1 pM	10 pM
19	3850	2106	1630	1865
20	3151	1717	2129	1842
21	3699	1866	1768	1609
22	2976	1795	1698	1491
23	2224	1921	1555	1402
24	2269	1608	1628	1417
average	3028.16667	1835.5	1734.66667	1604.33333

normalizing signal to average ctrl *100:

Percentage cell survival

biological replicate	ctrl	0 µM	1 pM	10 pM
19	127.139633	69.5470307	53.8279487	61.5884198
20	104.05636	56.7009742	70.3065661	60.8288844
21	122.153118	61.6214431	58.3851615	53.1344598
22	98.2772855	59.2767901	56.0735318	49.2377126
23	73.4437779	63.4377236	51.3512026	46.2986405
24	74.9298255	53.1014365	53.7619021	46.7939898
average	100	60.614233	57.2843855	52.9803511

ls pre-treated with deucravacitinib and afterwards transfected with poly(I:C) or

polyIC						
100 pM	1 nM	10 nM	100 nM	1 µM	10 µM	Deucravacitinib
2358	3023	3443	3605	3390	3624	
2105	2282	3378	3494	3236	3583	
2292	2585	3322	3196	3030	3274	
2348	2671	3176	3389	3395	3411	
1938	2524	2949	3060	2635	2598	
2242	2671	3121	2722	3447	2477	
2213.83333	2626	3231.5	3244.33333	3188.83333	3161.16667	

polyIC						
100 pM	1 nM	10 nM	100 nM	1 µM	10 µM	Deucravacitinib
73.6529752	94.4244885	107.543339	112.603467	105.887865	113.196939	
65.7504295	71.2790879	105.513041	109.136342	101.07762	111.916289	
71.5914415	80.7434015	103.763861	99.8282055	94.643136	102.264563	
73.3406216	83.4296424	99.2034984	105.85663	106.044042	106.543808	
60.5341246	78.8380447	92.113072	95.5801968	82.3051695	81.1494612	
70.0296736	83.4296424	97.4855536	85.0226456	107.66828	77.3699828	
69.1498777	82.0240512	100.937061	101.337915	99.6043521	98.7401739	

polyIC						
100 pM	1 nM	10 nM	100 nM	1 µM	10 µM	Deucravacitinib
2479	2352	3353	3443	3063	3466	
2030	2230	3201	3327	3230	3408	
1772	1824	3158	3272	3372	3418	
1865	2287	2808	3156	2953	3061	
1904	2200	1180	2985	2950	3078	
1865	2223	2994	3471	2936	3260	
1985.83333	2186	2782.33333	3275.66667	3084	3281.83333	

polyIC						
100 pM	1 nM	10 nM	100 nM	1 µM	10 µM	Deucravacitinib
78.18545	74.1799832	105.750631	108.589151	96.6042893	109.31455	
64.0243902	70.3322119	100.956686	104.930614	101.87132	107.485282	

55.8873003	57.5273339	99.6005046	103.195963	106.349874	107.800673
58.8204373	72.1299411	88.5618167	99.5374264	93.1349874	96.5412111
60.0504626	69.3860387	37.216148	94.1442389	93.0403701	97.0773759
58.8204373	70.1114382	94.4280908	109.472246	92.5988225	102.817494
62.631413	68.9444912	87.7523129	103.311606	97.2666106	103.506098

polyIC						
100 pM	1 nM	10 nM	100 nM	1 µM	10 µM	Deucravacitinib
2303	2675	3512	3090	3324	3225	
2587	2378	3291	3599	2859	3106	
2543	2577	3270	3653	3421	2778	
2304	2472	3711	3577	2733	2487	
2211	2539	3699	3366	3324	4101	
1927	2132	2838	2988	2731	3431	
2312.5	2462.16667	3386.83333	3378.83333	3065.33333	3188	

polyIC						
100 pM	1 nM	10 nM	100 nM	1 µM	10 µM	Deucravacitinib
73.107243	84.916142	111.486165	98.0900481	105.518227	102.375536	
82.122639	75.4880694	104.470663	114.247923	90.7571028	98.5979578	
80.7258875	81.8051955	103.804032	115.962118	108.597429	88.1858103	
73.1389874	78.4720385	117.803291	113.549548	86.7573144	78.9482038	
70.1867626	80.5989101	117.422359	106.851489	105.518227	130.183588	
61.1713666	67.6789588	90.0904714	94.8521242	86.6938257	108.914872	
73.4088143	78.1598857	107.51283	107.258875	97.3070208	101.200995	

polyIC						
100 pM	1 nM	10 nM	100 nM	1 µM	10 µM	Deucravacitinib
1561	2301	3305	3178	3188	2770	
2195	2113	2980	3195	3028	3386	
2027	2030	2895	2946	2825	3393	
1529	1941	3128	2683	2833	2903	
2002	2029	2775	2757	2689	3246	
1815	2018	2610	2718	2882	3049	
1854.83333	2072	2948.83333	2912.83333	2907.5	3124.5	

polyIC						
100 pM	1 nM	10 nM	100 nM	1 µM	10 µM	Deucravacitinib
51.5493423	75.9865705	109.141945	104.947988	105.278221	91.4744895	
72.4861027	69.7781936	98.4093786	105.509384	99.9944961	111.816831	
66.9381914	67.0372613	95.6023997	97.286587	93.29077	112.047994	
50.4925973	64.0981892	103.296824	88.601464	93.5549562	95.8665859	
66.1126094	67.004238	91.6396059	91.0451869	88.7996037	107.193571	
59.9372558	66.6409819	86.1907645	89.7572789	95.173097	100.687985	
61.2526831	68.4242391	97.380153	96.1913149	96.0151907	103.181243	

Figure 5b: Image and quantification of We

anti-pSTAT1

plotted values in red

shown in figure:

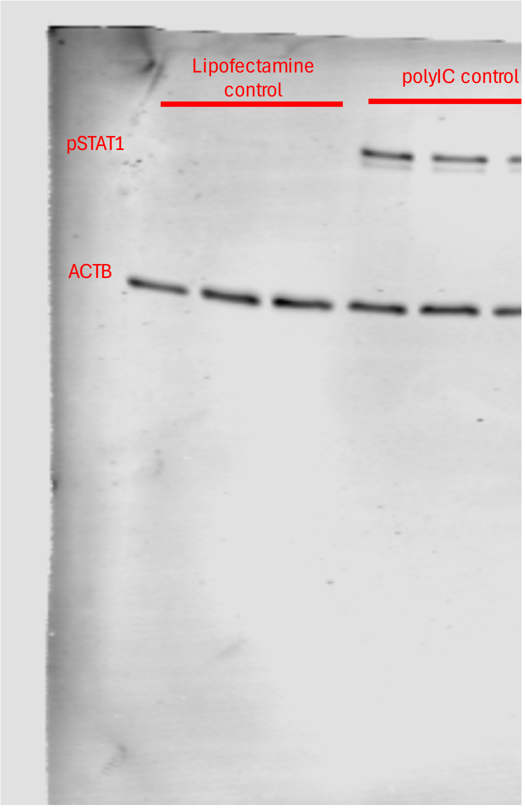

additional blots for quantification (all blots have the sa

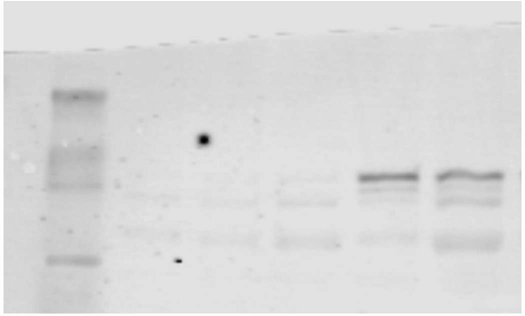

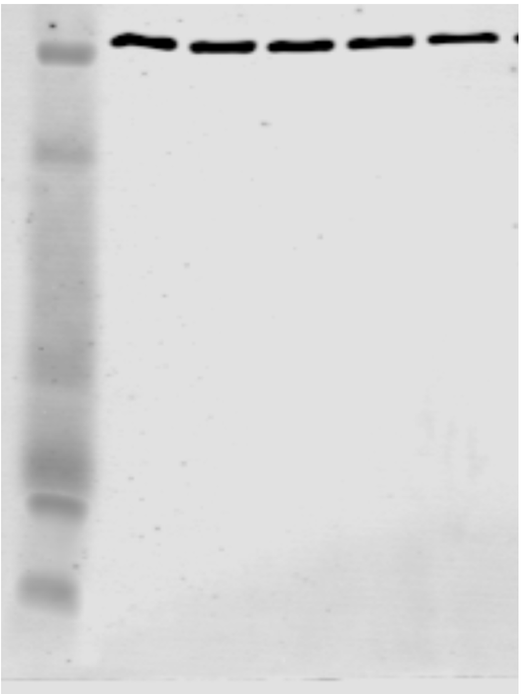

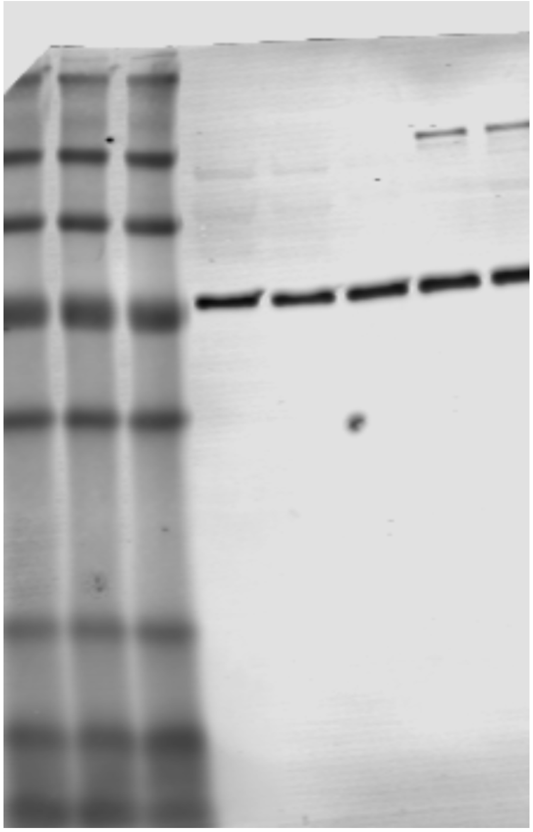

stern blot of pSTAT1Y701 in ReN VM-derived neural cells 24 hoursh after treatment with 10 μM de

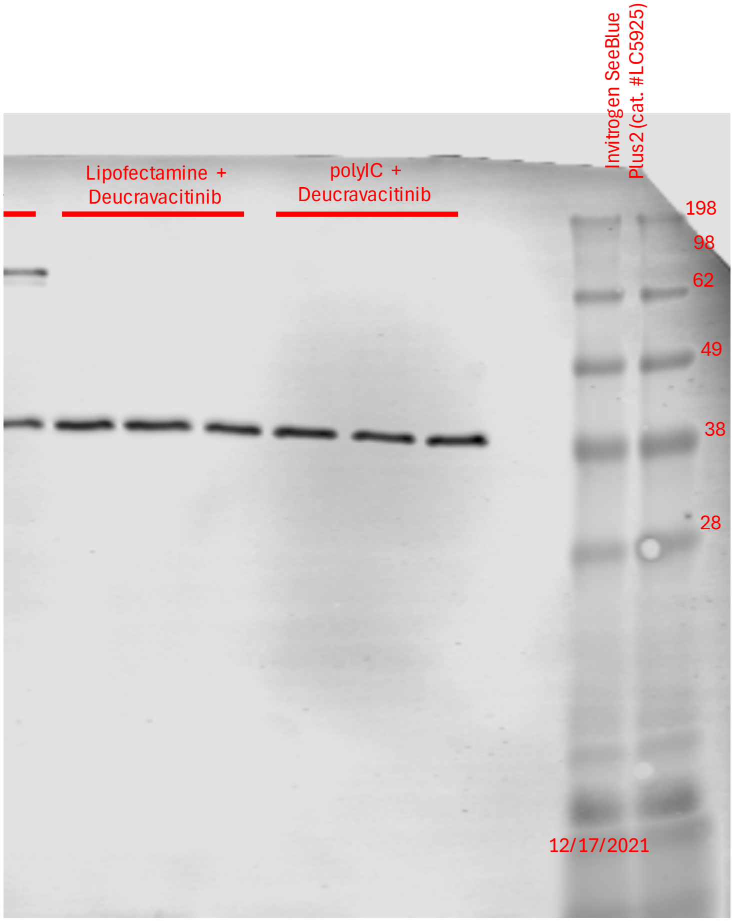

me layout and protein ladders, hence the annotations stay the same):

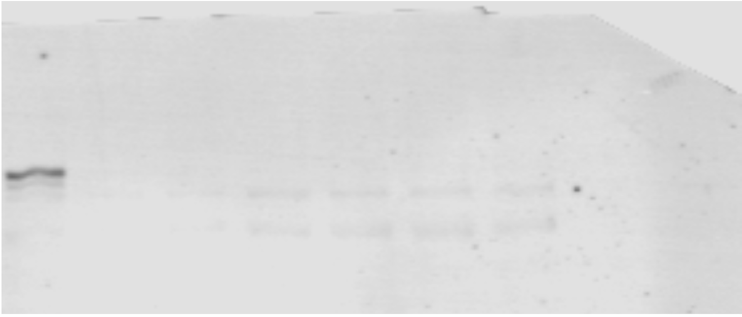

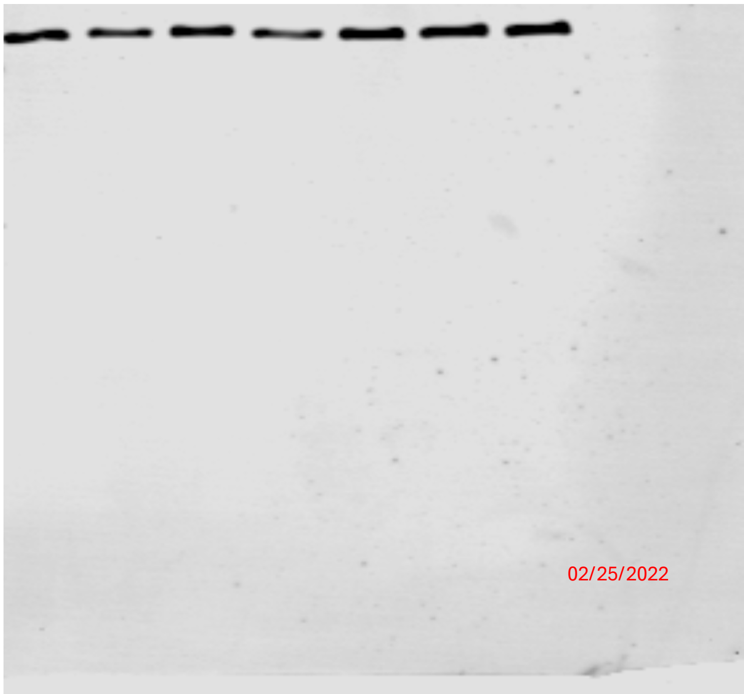
02/25/2022

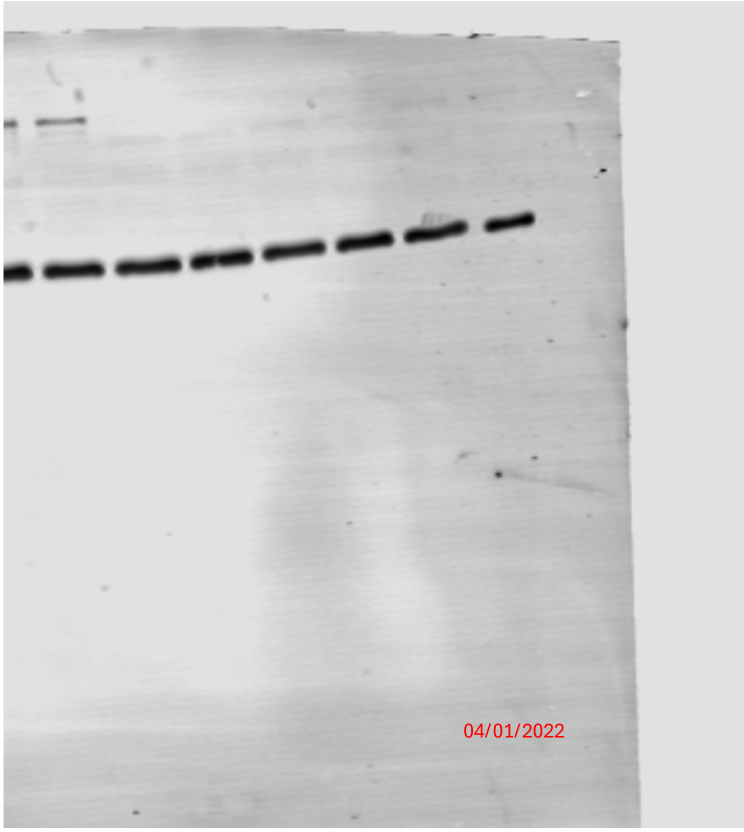
04/01/2022

eucravacitinib and transfection with poly(I:C) normalized to the housekeeping protein beta actin (ACTB)

Quantification of bands with ImageStudioLite

	Lipofectamine ctrl	poly(I:C) control	drug + lipo
pSTAT1	1.125	201.4287109	1.37792969
	0.98828125	162.6259766	3.24023438
	8.517578125	150.2382813	7.63769531
Actin	7544.191406	10296.50293	12893.2803
	11098.33203	10999.93066	12799.5488
	11866.16113	8165.238281	11020.832
Normalized to Actin	0.000149121	0.019562828	0.00010687
	8.90477E-05	0.014784273	0.00025315
	0.000717804	0.018399742	0.00069302
Average	0.000318658	0.017582281	0.00035102
Normalized to dsRNA	0.848134264	111.2644462	0.6078388
*100	0.506462888	84.08620545	1.43981452
	4.082542072	104.6493483	3.94160165
Avergae	1.812379741	100	1.99641832

Quantification of bands with ImageStudioLite

	Lipofectamine ctrl	poly(I:C) control	drug + lipo
pSTAT1	1.793457031	80.69921875	1.05761719
	0.905273438	67.32397461	2.05126953
	3.514648438	101.5654297	6.60595703
Actin	18704.93359	14222.94043	7327.21094

	16456.6709	11612.76758	10856.9385
	14894.23633	12319.79297	7522.11523
Normalized to Actin	9.58815E-05	0.005673877	0.00014434
	5.50095E-05	0.00579741	0.00018894
	0.000235974	0.008244086	0.0008782
Average	0.000128955	0.006571791	0.00040383
Normalized to dsRNA	1.458985788	86.33684923	2.19637283
*100	0.837055105	88.21659072	2.87495869
	3.590706482	125.4465601	13.3632477
Avergae	1.962249125	100	6.14485974

Quantification of bands with ImageStudioLite

	Lipofectamine ctrl	poly(I:C) control	drug + lipo
pSTAT1	1.88671875	88.74121094	0.89746094
	1.958007813	80.36523438	0.60253906
	1.408203125	66.54003906	2.44140625
Actin	8756.472656	7918.285156	8931.5752
	6387.505859	9967.235352	10004.8906
	7697.80957	8689.334961	7594.57715
Normalized to Actin	0.000215466	0.011207125	0.00010048
	0.000306537	0.008062941	6.0224E-05
	0.000182936	0.007657668	0.00032147
Average	0.000234979	0.008975911	0.00016072
Normalized to dsRNA	2.400487323	124.8577948	1.11946094
*100	3.415108893	89.82866587	0.67095641
	2.038072306	85.31353936	3.58144182
Avergae	2.617889507	100	1.79061972

drug + poly(I:C)
0.390625
3.86816406
5.42578125
14564.3457
12673.876
14283.8594
2.6821E-05
0.00030521
0.00037985
0.00023729
0.15254355
1.73588207
2.16043647
1.34962069

drug + poly(I:C)
3.37451172
2.06103516
3.22851563
13359.6572

12764.9521
14678.9004
0.00025259
0.00016146
0.00021994
0.00021133
3.84354422
2.45687163
3.34676814
3.21572799

drug + poly(I:C)
3.2578125
1.95800781
3.16210938
10147.0068
9760.00195
8033.48828
0.00032106
0.00020062
0.00039362
0.0003051
3.5769229
2.23504341
4.38524811
3.39907148

Figure 5c: Detected CellTiter-Glo luminescence signal - Cell survival of ReN CX cell-derived neuro
afterwards transfected with poly(I:C) or lipofectamine

plotted values in red

experiment 1

	poly(I:C)			
biological replicate	10 µM	1 µM	0.1 µM	0.01 µM
1	1802	3534	3575	3480
2	1883	3238	3975	3464
3	2228	3578	5002	4109
4				
5				
6				
average	1971	3450	4184	3684.33333

normalizing signal to average ctrl *100:

Percentage cell survival

	poly(I:C)			
biological replicate	10 µM	1 µM	0.1 µM	0.01 µM
1	62.2846938	122.149893	123.567026	120.283426
2	65.0843943	111.918889	137.392707	119.730399
3	77.0090443	123.670718	172.890143	142.02431
4				
5				
6				
average	68.1260441	119.2465	144.616625	127.346045

experiment 2

	poly(I:C)			
biological replicate	10 µM	1 µM	0.1 µM	0.01 µM
7	6084	10710	14954	10780
8	5041	9402	13352	11363
9	5008	10355	15739	11511
10				
11				
12				
average	5377.66667	10155.6667	14681.6667	11218

normalizing signal to average ctrl *100:

Percentage cell survival

	poly(I:C)			
biological replicate	10 µM	1 µM	0.1 µM	0.01 µM
7	56.0565111	98.6793612	137.782555	99.3243243
8	46.4465602	86.6277641	123.022113	104.695946

9	46.1425061	95.4084767	145.015356	106.059582
10				
11				
12				
average	49.5485258	93.5718673	135.273342	103.359951

experiment 3

	poly(I:C)			
biological replicate	10 µM	1 µM	0.1 µM	0.01 µM
13	6084	10710	14954	10780
14	5041	9402	13352	11363
15	5008	10355	15739	11511
16				
17				
18				
average	5377.66667	10155.6667	14681.6667	11218

normalizing signal to average ctrl *100:

Percentage cell survival

	poly(I:C)			
biological replicate	10 µM	1 µM	0.1 µM	0.01 µM
13	56.0565111	98.6793612	137.782555	99.3243243
14	46.4465602	86.6277641	123.022113	104.695946
15	46.1425061	95.4084767	145.015356	106.059582
16				
17				
18				
average	49.5485258	93.5718673	135.273342	103.359951

ns pre-treated with deucravacitinib and

0 μM	lipofectamine
1547	2577
1785	3094
1492	2973
1857	3094
1700	2782
2040	2839
1736.83333	2893.16667

0 μM	lipofectamine
53.4708221	89.0719511
61.6971024	106.941644
51.5697909	102.759376
64.185725	106.941644
58.7591451	96.1576128
70.5109741	98.1277723
60.0322599	100

0 μM	lipofectamine
6804	11672
6622	10632
7335	11106
6649	10885
7843	9972
6340	10853
6932.16667	10853.3333

0 μM	lipofectamine
62.6904177	107.542998
61.0135135	97.960688

67.5829238	102.32801
61.262285	100.291769
72.2635135	91.8796069
58.4152334	99.9969287
63.8713145	100

0 µM	lipofectamine
6804	11672
6622	10632
7335	11106
6649	10885
7843	9972
6340	10853
6932.16667	10853.3333

0 µM	lipofectamine
62.6904177	107.542998
61.0135135	97.960688
67.5829238	102.32801
61.262285	100.291769
72.2635135	91.8796069
58.4152334	99.9969287
63.8713145	100

Figure 5d: Image and quantification of Western blot of pSTAT1Y701 in ReN CX-derived neural

anti-pSTAT1
plotted values in red

shown in figure:

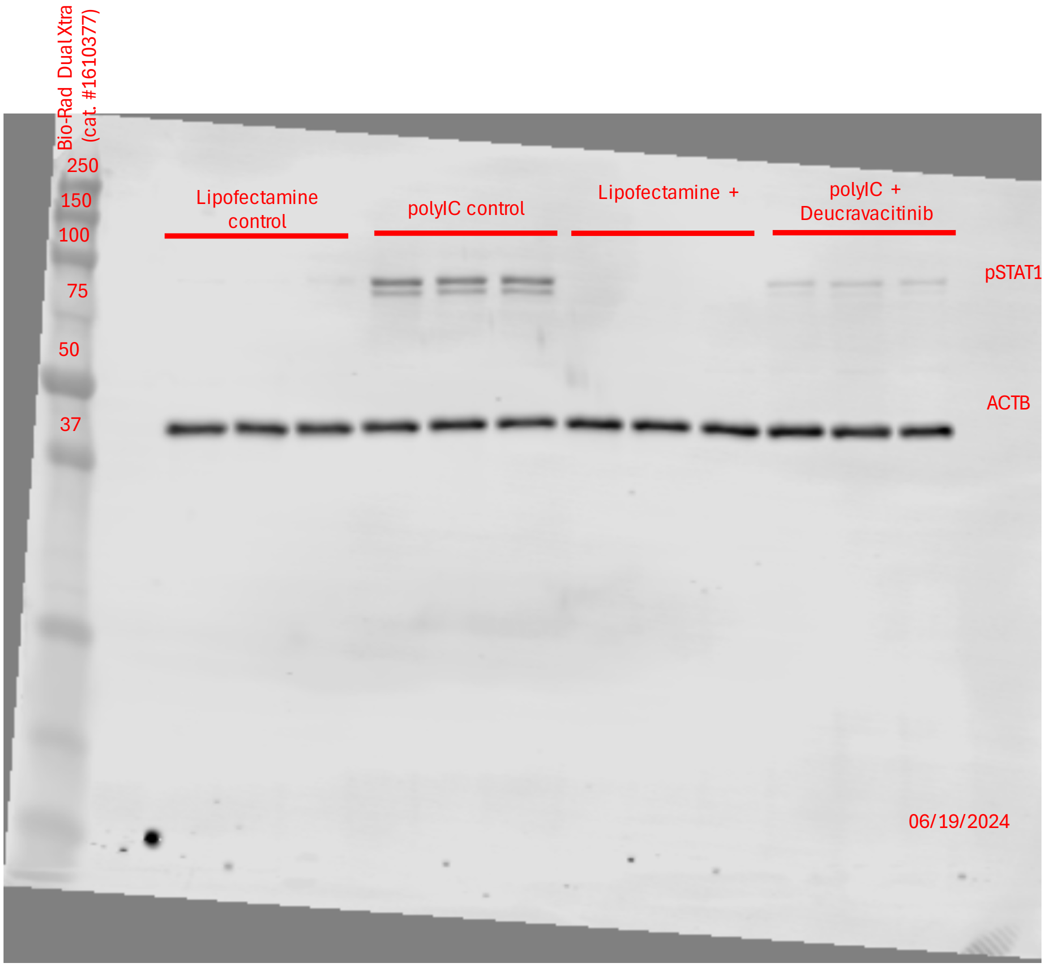

additional blots for quantification (all blots have the same layout and protein ladders, hence the

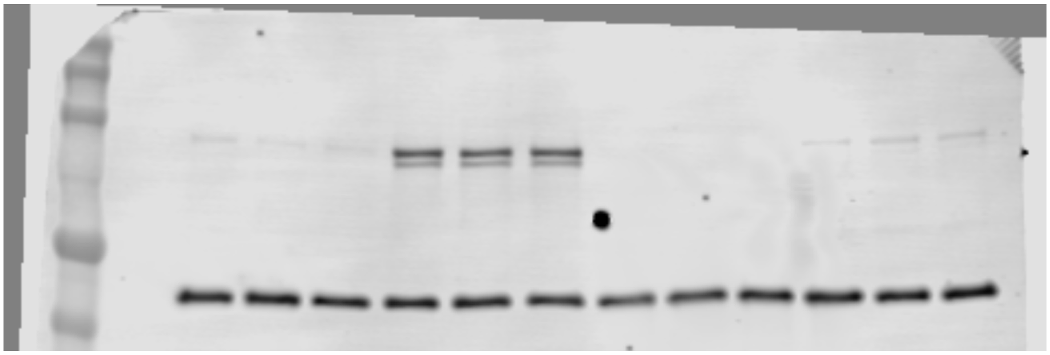

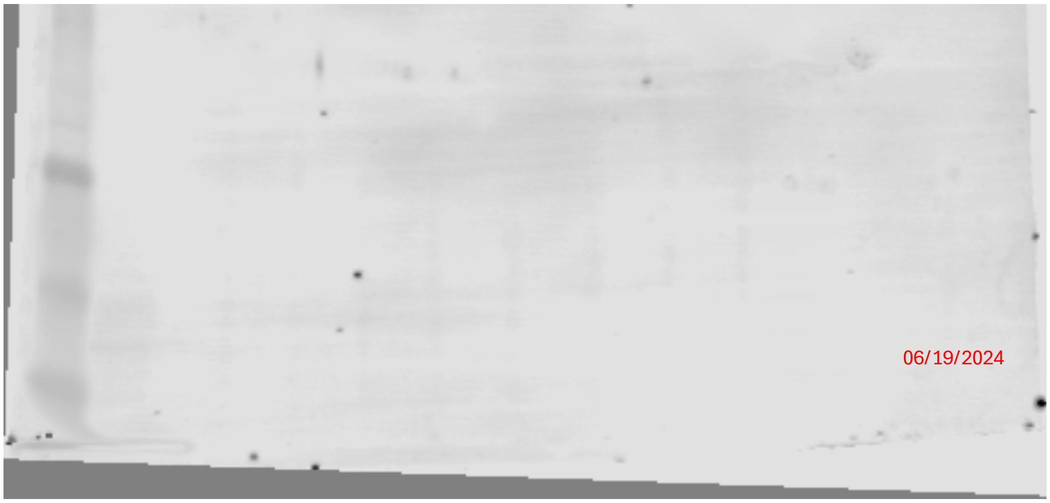
06/19/2024

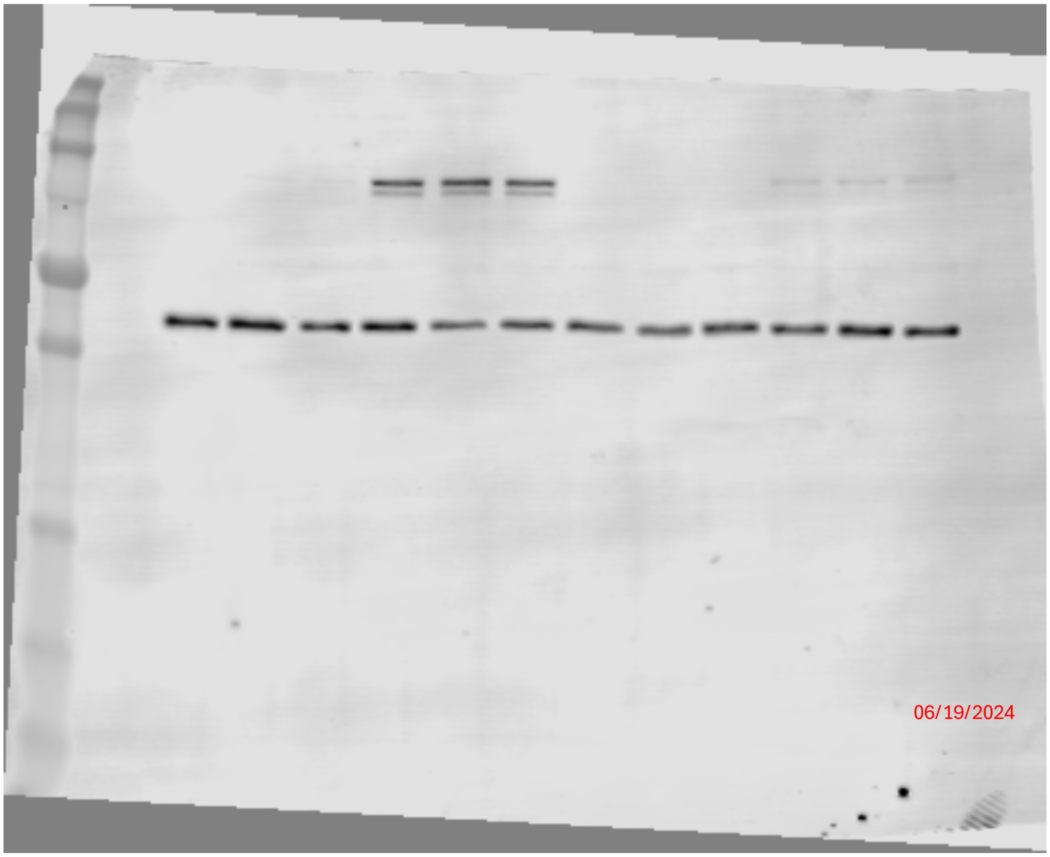
06/19/2024

cells 24 hoursh after treatment with 10 µM deucravacitinib and transfection with poly(I:C) norm
(ACTB)

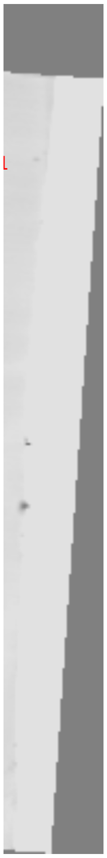

Quantification of bands with ImageStudioLite

	Lipofectamine ctrl	poly(I:C) control
pSTAT1	11.72363281	495.6894531
	12.23242188	455.1767578
	21.54003906	477.6835938
Actin	36629.06445	35756.46484
	32638.98242	40318.99414
	33402.59766	34476.23047
Normalized t	0.000320064	0.013862932
	0.00037478	0.011289388
	0.000644861	0.013855447
Average	0.000446568	0.013002589
Normalized t *100	2.461537986	106.616707
	2.882345259	86.82415192
	4.95948299	106.5591411
Avergae	3.434455412	100

[illegible] annotations stay the same):

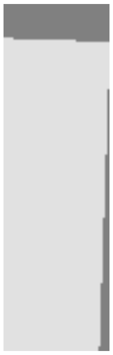

Quantification of bands with ImageStudioLite

	Lipofectamine ctrl	poly(I:C) control
pSTAT1	25.3046875	438.0234375
	26.18261719	405.2451172
	28.08691406	436.0498047
Actin	30937.37891	30546.13086

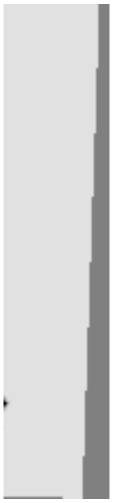

	34309.22461	31102.8418
	30812.82617	25588.44141
Normalized t	0.000817932	0.014339736
	0.000763136	0.013029199
	0.000911533	0.01704089
Average	0.000830867	0.014803275
Normalized t *100	5.525348343	96.86867118
	5.155186175	88.01565402
	6.157645642	115.1156748
Avergae	5.61272672	100

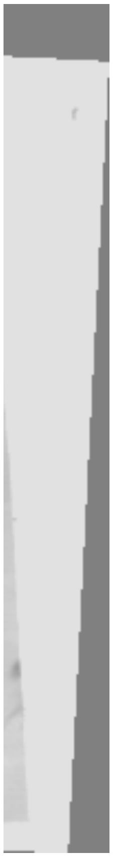

Quantification of bands with ImageStudioLite

	Lipofectamine ctrl	poly(I:C) control
pSTAT1	2.419921875	401.4160156
	11.68554688	423.2246094
	23.02539063	358.5527344
Actin	20956.11914	22055.23828
	25967.38672	8624.615234
	14237.29102	12001.38867
Normalized t	0.000115476	0.018200484
	0.000450009	0.049071709
	0.001617259	0.029875937
Average	0.000727581	0.03238271
Normalized t *100	0.356596679	56.20432665
	1.389656949	151.5367578
	4.994205998	92.25891556
Avergae	2.246819875	100

nalized to the housekeeping protein beta actin

drug + lipo	drug + poly(I:C)
6.7578125	52.5
0.736328125	64.28710938
1.453125	35.72851563
43812.82227	48624.56055
42134.42578	43132.5
47128.37305	40872.52441
0.000154243	0.001079701
1.74757E-05	0.001490456
3.08333E-05	0.000874145
6.75173E-05	0.001148101
1.186246841	8.303740621
0.13440161	11.46276604
0.237132286	6.722853885
0.519260246	8.829786848

drug + lipo	drug + poly(I:C)
12.08300781	20.14355469
5.12109375	19.64746094
4.319335938	30.04296875
18459.90234	34890.95898

23526.06445	31557.98633
29332.61328	38860.14648
0.000654554	0.000577329
0.000217677	0.000622583
0.000147254	0.000773105
0.000339828	0.000657672
4.421685589	3.900007182
1.470468227	4.205710274
0.994737355	5.222526036
2.295630391	4.442747831

drug + lipo	drug + poly(I:C)
6.314453125	47.1328125
6.873046875	45.1484375
13.48046875	47.296875
11540.23828	13510.69922
9289.894531	22956.56836
15125.9375	20085.08594
0.000547168	0.003488555
0.000739841	0.001966689
0.000891215	0.002354826
0.000726075	0.002603357
1.689692883	10.77289278
2.284679737	6.073269647
2.75213352	7.271860854
2.242168713	8.039341094

Figure 5e: Detected CellTiter-Glo luminescence signal - Cell survival of SH-SY5Y cells pre-treated wi transfected with poly(I:C) or lipofectamine

plotted values in red

experiment 1

	poly(I:C)				
biological rep	10 µM	1 µM	0.1 µM	0.01 µM	0 µM
1	4520	4681	4406	3032	2110
2	5646	4572	4519	3401	2015
3	3425	5047	5013	3665	1748
4					1690
5					2092
6					2165
average	4530.33333	4766.66667	4646	3366	1970

normalizing signal to average ctrl *100:

Percentage cell survival

	poly(I:C)				
biological rep	10 µM	1 µM	0.1 µM	0.01 µM	0 µM
1	75.0539658	77.7273482	73.1610118	50.3459346	35.0362539
2	93.7510378	75.9174185	75.0373609	56.4731278	33.4587923
3	56.8716444	83.8047269	83.2401616	60.8568108	29.0252947
4					28.0622129
5					34.7373665
6					35.9495212
average	75.2255493	79.1498312	77.1461781	55.8919577	32.7115736

experiment 2

	poly(I:C)				
biological rep	10 µM	1 µM	0.1 µM	0.01 µM	0 µM
7	5661	6427	6152	3569	2538
8	5334	5677	5472	4308	2761
9	5355	6793	6571	5100	2353
10					3048
11					1948
12					2747
average	5450	6299	6065	4325.66667	2565.83333

normalizing signal to average ctrl *100:

Percentage cell survival

	poly(I:C)				
biological rep	10 µM	1 µM	0.1 µM	0.01 µM	0 µM
7	82.8722003	94.0857854	90.0600205	52.2471088	37.1541502

8	78.0851998	83.1064266	80.1054018	63.065437	40.4186795
9	78.3926219	99.4437125	96.1938223	74.6596399	34.4459084
10					44.6201142
11					28.5170546
12					40.2137315
average	79.7833407	92.2119748	88.7864149	63.3240619	37.5616064

experiment 3

	poly(I:C)				
biological rep	10 μM	1 μM	0.1 μM	0.01 μM	0 μM
13	2735	3666	2733	1989	1216
14	2729	3454	3038	1918	1142
15	3608	4050	2931	2280	1161
16					1157
17					1190
18					1230
average	3024	3723.33333	2900.66667	2062.33333	1182.66667

normalizing signal to average ctrl *100:

Percentage cell survival

	poly(I:C)				
biological rep	10 μM	1 μM	0.1 μM	0.01 μM	0 μM
13	81.4149633	109.128795	81.3554277	59.2081762	36.1976583
14	81.2363564	102.818019	90.43461	57.0946616	33.9948402
15	107.402262	120.559635	87.2494543	67.8706092	34.5604287
16					34.4413574
17					35.4236952
18					36.6144076
average	90.0178607	110.835483	86.3464973	61.391149	35.2053979

experiment 4

	poly(I:C)				
biological rep	10 μM	1 μM	0.1 μM	0.01 μM	0 μM
13	2605	3375	2507	1990	1185
14	2127	2917	2275	1422	1065
15	2321	3374	2840	2057	730
16					984
17					955
18					1086
average	2351	3222	2540.66667	1823	1000.83333

normalizing signal to average ctrl *100:

		poly(I:C)				
Percentage cell survival	biological rep	10 µM	1 µM	0.1 µM	0.01 µM	0 µM
	13	77.1394729	99.9407758	74.2374889	58.9280426	35.0903168
	14	62.9848978	86.3784424	67.3674859	42.1083802	31.536867
	15	68.7296417	99.9111638	84.0983121	60.9120521	21.6168197
	16					29.1382884
	17					28.2795381
	18					32.1587208
	average	69.6180041	95.4101273	75.234429	53.982825	29.6367585

th deucravacitinib and afterwards

lipofectamine + 10 µM drug
5878
6227
5962
6022.333333

lipofectamine + 10 µM drug
97.60336525
103.3984613
98.99817347
100

lipofectamine + 10 µM drug
6984
6594
6915
6831

lipofectamine + 10 µM drug
102.2397892

96.53052262
101.2296882
100
lipofectamine + 10 μM drug
3165
2867
4046
3359.333333
lipofectamine + 10 μM drug
94.21512205
85.34431435
120.4405636
100
lipofectamine + 10 μM drug
3718
2965
3448
3377

lipofectamine + 10 μM drug
110.0977199
87.79982233
102.1024578
100

Figure 5f: Image and quantification of Western blot of pSTAT1Y701 in SH-SY5Y c

anti-pSTAT1
plotted values in red

shown in figure:

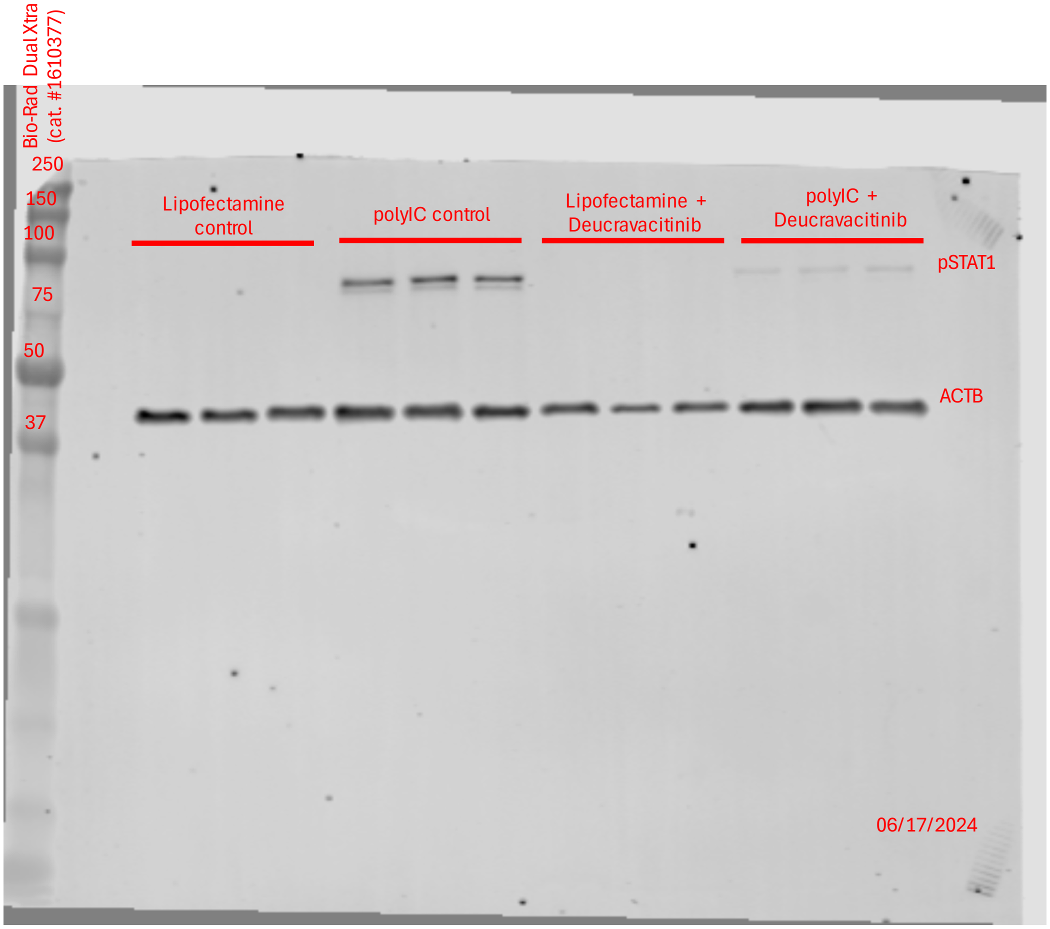

additional blots for quantification (all blots have the same layout and protein ladders, hence the

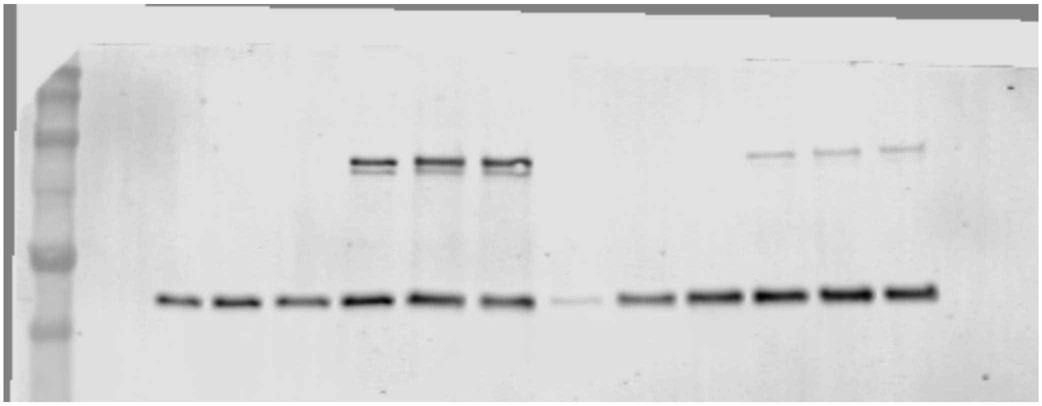

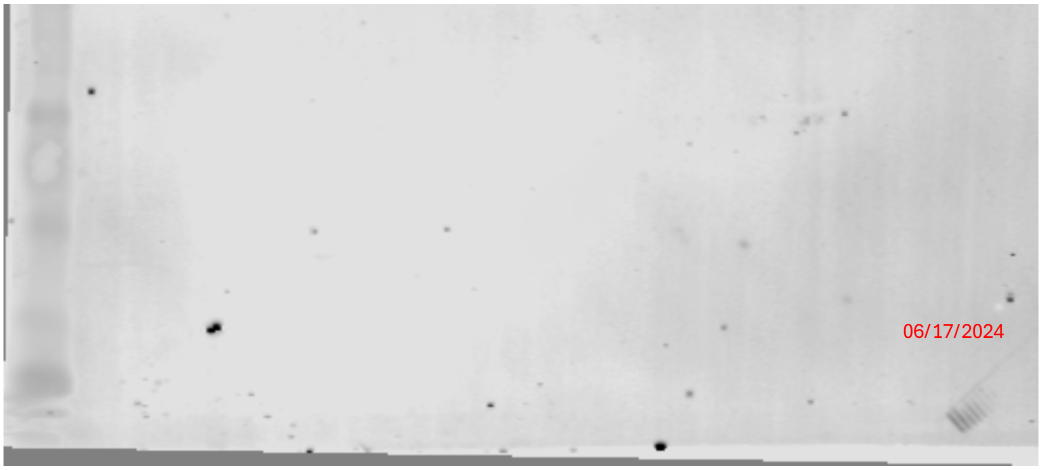
06/17/2024

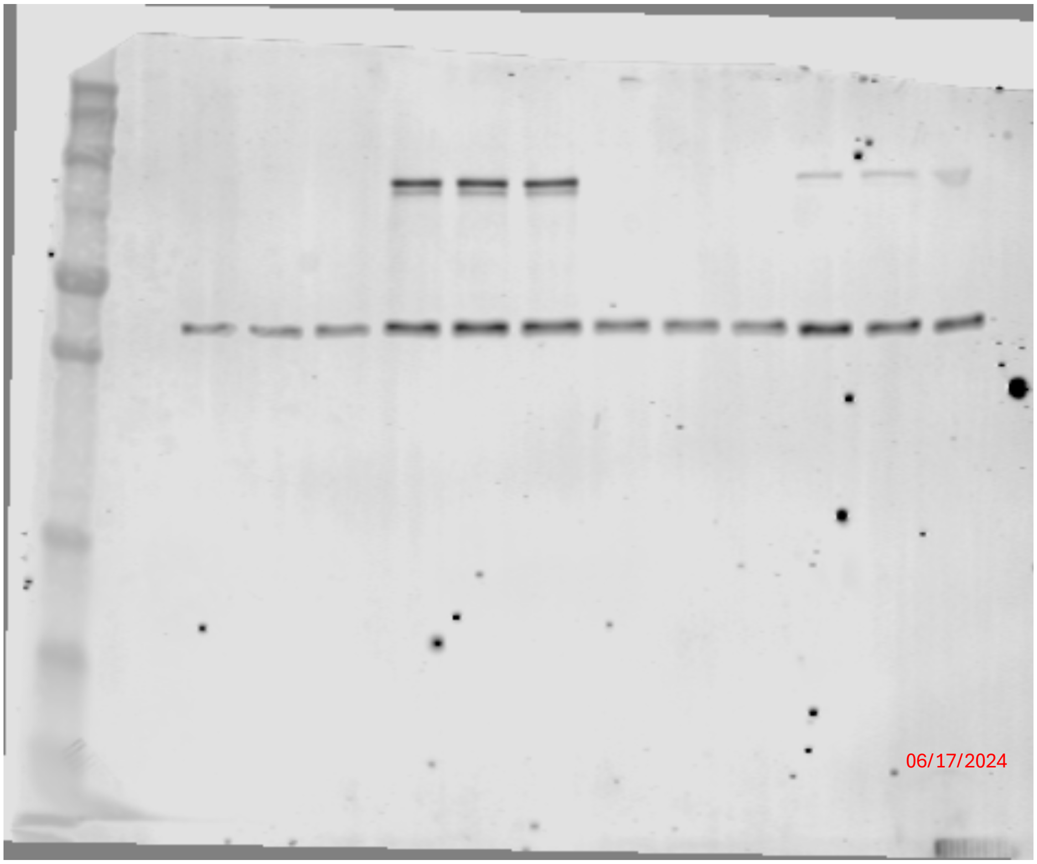
06/17/2024

ells 24 hoursh after treatment with 10 µM deucravacitinib and transfection with poly(I:C) no

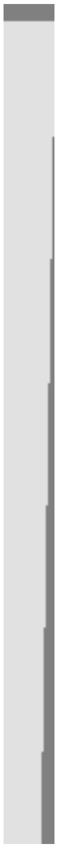

Quantification of bands with ImageStudioLite

	Lipofectamine ctrl
pSTAT1	1.8359375
	5.902832031
	0.237304688
Actin	18042.01953
	13495.64648
	13976.95313
Normalized to Actin	0.000101759
	0.000437388
	1.69783E-05
Average	0.000185375
Normalized to dsRNA *100	0.867533476
	3.728895646
	0.144746245
Avergae	1.580391789

: annotations stay the same):

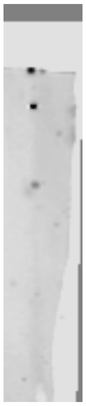

Quantification of bands with ImageStudioLite

	Lipofectamine ctrl
pSTAT1	1.312011719
	1.401367188
	0.034179688
Actin	10326.67285
	17194.58691
	11718.22852
Normalized to Actin	0.000127051

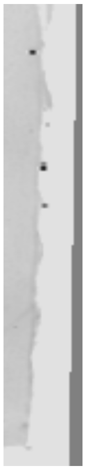

	8.15005E-05
	2.9168E-06
Average	7.04894E-05
Normalized to dsRNA *100	1.502525533
	0.963839595
	0.034494566
Avergae	0.833619898

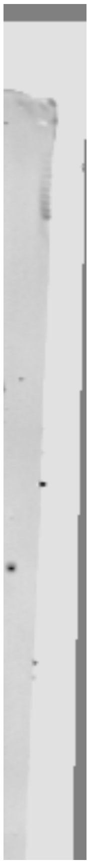

Quantification of bands with ImageStudioLite

	Lipofectamine ctrl
pSTAT1	1.045410156
	0.107910156
	0.055175781
Actin	4407.724609
	3830.300781
	4532.228516
Normalized to Actin	0.000237177
	2.81728E-05
	1.21741E-05
Average	9.25079E-05
Normalized to dsRNA *100	1.359496867
	0.161486191
	0.069781876
Avergae	0.530254978

rmalized to the housekeeping protein beta actin (ACTB)

poly(I:C) control	drug + lipo	drug + poly(I:C)
188.0942383	1.156738281	17.61181641
199.3325195	0.208984375	21.90869141
173.4575195	0.375976563	24.53710938
17865.21875	10864.78125	13850.70508
14808.98242	5877.835938	16201.625
15486.85352	8613.732422	11567.58594
0.010528516	0.000106467	0.001271547
0.013460244	3.55546E-05	0.001352253
0.011200307	4.36485E-05	0.002121195
0.011729689	6.189E-05	0.001581665
89.75954699	0.907669244	10.84041143
114.7536311	0.303116693	11.52846168
95.48682193	0.372119899	18.083986
100	0.527635279	13.48428637

poly(I:C) control	drug + lipo	drug + poly(I:C)
161.4404297	0.022460938	26.68774414
185.1113281	0.581054688	25.27954102
178.1430664	0.353271484	22.94677734
26778.77344	310.578125	31441.15625
23247.74805	10108.52148	34627.38672
15659.24609	24370.85156	27051.02734
0.006028672	7.23198E-05	0.000848816

0.007962549	5.74817E-05	0.000730045
0.011376222	1.44957E-05	0.000848277
0.008455814	4.8099E-05	0.000809046
71.29616822	0.855266736	10.03824803
94.16655278	0.679788692	8.633641931
134.537279	0.171428271	10.03188405
100	0.5688279	9.567924669

poly(I:C) control	drug + lipo	drug + poly(I:C)
258.4384766	0.610107422	23.33959961
264.8706055	1.162109375	24.81054688
251.4326172	0.048828125	18.94238281
14211.77734	6715.023438	18759.51367
16800.28418	5210.712891	11945.33887
13674.38086	5876.439453	12080.42969
0.01818481	9.08571E-05	0.001244147
0.015765841	0.000223023	0.002077007
0.01838713	8.30913E-06	0.001568022
0.017445927	0.000107396	0.001629725
104.2352772	0.520792503	7.131448896
90.3697508	1.278367758	11.90539505
105.394972	0.047627933	8.987898836
100	0.615596065	9.341580929

Figure 6a: Detected CellTiter-Glo luminescence signal - Cell survival of control and TYK2 knockdown
and afterwards transfected with poly(I:C) or lipofectamir

plotted values in red

		ReN VM TYK2 KD			
Experiment 1	Biological replicate	polyIC			
		0 µM	10 µM	1 µM	0.1 µM
	1	5008	6071	6119	6110
	2	5114	5640	6133	5999
	3	4829	5773	5766	5634
	4	5151	5566	5814	5726
	5	5170	6074	6230	5987
	6	5144	5274	5867	5752
	average	5069.33333	5733	5988.16667	5868
Percentage c normalizing signal to average lipo		80.6571106	97.7774199	98.5504912	98.4055403
		82.3643099	90.8358834	98.7759704	96.6178129
		77.7741987	92.9779353	92.8651957	90.7392495
		82.960219	89.6440651	93.638267	92.2209696
		83.2662264	97.8257368	100.338219	96.424545
		82.8474795	84.9412144	94.4918666	92.6397165
	average	81.644924	92.3337091	96.4433349	94.5079723

		ReN VM TYK2 KD			
Experiment 2	Biological replicate	polyIC			
		0 µM	10 µM	1 µM	0.1 µM
	7	5257	5285	5765	5738
	8	5463	5627	5607	5530
	9	5428	5871	5754	6244
	10	4920	5969	5495	5765
	11	5217	5554	5801	6116
	12	4943	5602	5887	5788
	average	5204.66667	5651.33333	5718.16667	5863.5
Percentage c normalizing signal to average lipo		86.1779733	86.6369771	94.5056146	94.0630037
		89.5549302	92.2433813	91.9155214	90.6532608
		88.9811754	96.243272	94.3252917	102.357859
		80.6535341	97.8497855	90.079506	94.5056146
		85.5222535	91.0466927	95.0957624	100.259556
		81.0305729	91.8335565	96.50556	94.8826535
	average	85.3200732	92.6422775	93.737876	96.1203246

		ReN VM TYK2 KD			

Experiment 3

	Biological replicate	polyIC			
		0 μM	10 μM	1 μM	0.1 μM
	13	3935	4283	3919	4194
	14	3807	4389	4092	4366
	15	3742	4228	4124	4400
	16	3671	4327	4339	4282
	17	3926	4547	4067	4438
	18	4018	4247	4070	4400
	average	3849.83333	4336.83333	4101.83333	4346.66667
Percentage c normalizing signal to average lipo		89.3810335	97.2856332	89.0176036	95.2640545
		86.4735945	99.693356	92.9471891	99.1709256
		84.9971607	96.036343	93.6740488	99.9432141
		83.3844407	98.2850653	98.5576377	97.2629188
		89.1766042	103.282226	92.3793299	100.80636
		91.266326	96.467916	92.447473	99.9432141
	average	87.4465266	98.5084232	93.170547	98.7317812

Experiment 4

		ReN VM TYK2 KD			
	Biological replicate	polyIC			
		0 μM	10 μM	1 μM	0.1 μM
	19	3731	4090	3855	4118
	20	3552	4134	4013	4192
	21	3490	4181	3784	4161
	22	3512	4057	4154	4144
	23	3869	4119	4399	4210
	24	3727	4036	4137	4190
	average	3646.83333	4102.83333	4057	4169.16667
Percentage c normalizing signal to average lipo		93.0656024	102.020454	96.1586431	102.718883
		88.6006485	103.117985	100.099776	104.564729
		87.0541282	104.290347	94.3876278	103.791469
		87.6028935	101.197306	103.616862	103.367423
		96.5078573	102.743826	109.728112	105.013719
		92.9658269	100.673485	103.192816	104.514842
	average	90.9661595	102.340567	101.197306	103.995178

Experiment 5

	ReN VM with scramble RNA	
Biological replicate	lipo	poly IC
1	5466	2081
2	5385	1885
3	5514	1764

	4	5389	1986
	5	5614	2243
	6	5656	2086
	7	5506	1522
	8	5456	1724
	9	5379	1229
	10	5416	1276
	11	5746	1384
	12	5648	1430
	average	5514.583	1717.5
Percentage c normalizing signal to average lipo		99.119	37.73631
		97.65017	34.18209
		99.98942	31.98791
		97.7227	36.0136
		101.8028	40.67397
		102.5644	37.82697
		99.84435	27.59955
		98.93767	31.26256
		97.54137	22.28636
		98.21232	23.13865
		104.1964	25.09709
		102.4193	25.93124
	average	99.9999917	31.1446917

(KD) ReN VM-derived neural cells pre-treated with deucravacitinib
ne as a vehicle control.

	lipofectamine only		
0.01 µM	0 µM	10 µM	Deucravacitinib
5904	6550	6534	
6052	6535	6118	
5799	5771	6364	
5625	6217	6378	
5574	6339	6049	
5820	5842	6155	
5795.66667	6209	6266.33333	
95.0877758	105.492028	105.234337	
97.4714125	105.250443	98.5343856	
93.3966822	92.9457239	102.496376	
90.5942986	100.128845	102.721855	
89.7729103	102.093735	97.4230955	
93.734901	94.0892253	99.1302947	
93.3429967	100	100.923391	

	lipofectamine only		
0.01 µM	0 µM	10 µM	Deucravacitinib
5389	6013	5873	
5845	6133	5872	
5611	6274	6095	
5245	6062	6309	
5848	5855	5981	
5766	6264	5989	
5617.33333	6100.16667	6019.83333	
88.3418486	98.5710773	96.276058	
95.8170542	100.538237	96.259665	
91.9810934	102.849649	99.9153029	
85.9812573	99.374334	103.423404	
95.8662332	95.9809841	98.0465015	
94.5220076	102.685719	98.1776454	
92.0849157	100	98.6830961	

	lipofectamine only		
0.01 µM	0 µM	10 µM	Deucravacitinib
4070	4336	3822	
4300	4433	3896	
4331	4409	3699	
4215	4445	4380	
4499	4484	4135	
4036	4308	4198	
4241.83333	4402.5	4021.66667	
92.447473	98.4894946	86.8143101	
97.6717774	100.692788	88.4951732	
98.3759228	100.147643	84.0204429	
95.7410562	100.965361	99.4889267	
102.191936	101.851221	93.9239069	
91.6751846	97.8534923	95.354912	
96.3505584	100	91.349612	

	lipofectamine only		
0.01 µM	0 µM	10 µM	Deucravacitinib
3933	3843	3615	
4222	3971	3714	
4059	3990	3766	
4121	4084	4270	
3964	3969	4532	
4128	4197	3953	
4071.16667	4009	3975	
98.1042654	95.8593165	90.1721127	
105.313046	99.0521327	92.6415565	
101.247194	99.5260664	93.9386381	
102.793714	101.870791	106.510352	
98.8775256	99.0022449	113.045647	
102.968321	104.689449	98.6031429	
101.550678	100	99.1519082	

Figure 6b: Image and quantification of Western blot of pSTAT1Y701 in control and TYK2 KD ReN
neuronal cells 24 hourh after treatment with 10 µM deucravacitinib and transfec-tion with
normalized to the housekeeping protein beta actin (ACTB).

anti-pSTAT1

shown in figure:

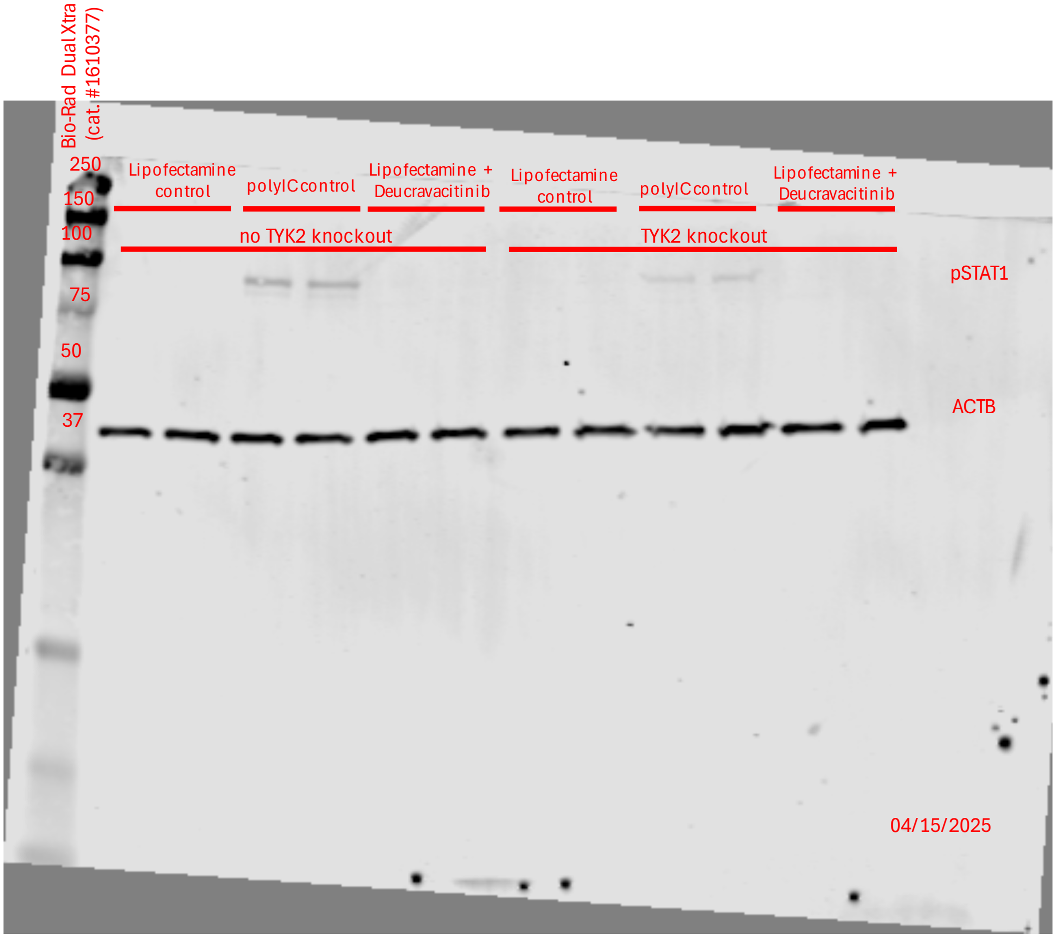

additional blots for quantification (all blots have the same layout and protein ladders, hence the

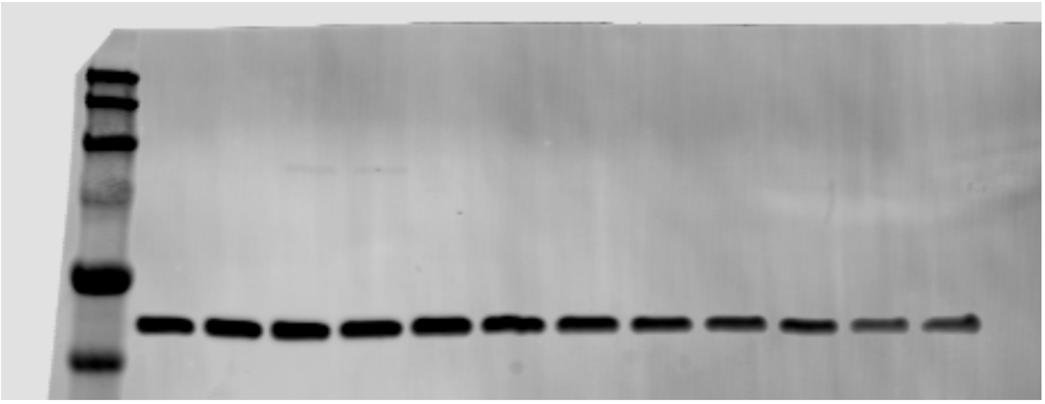

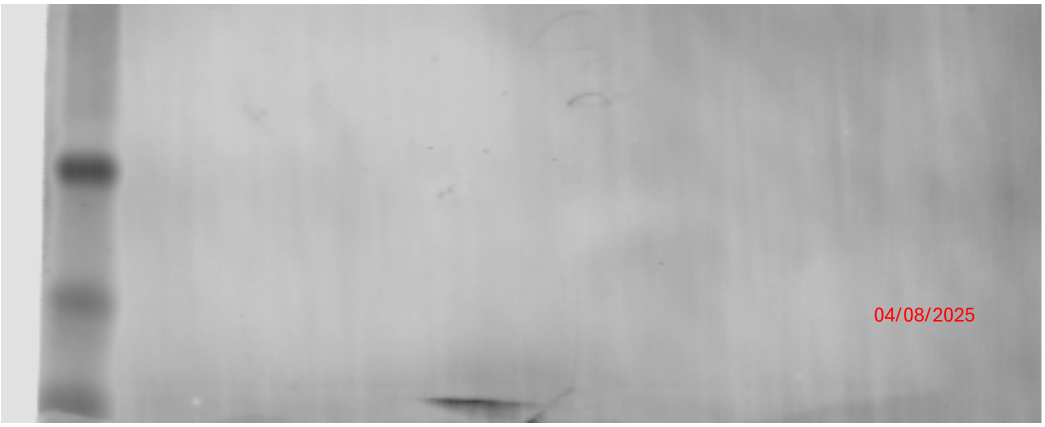
04/08/2025

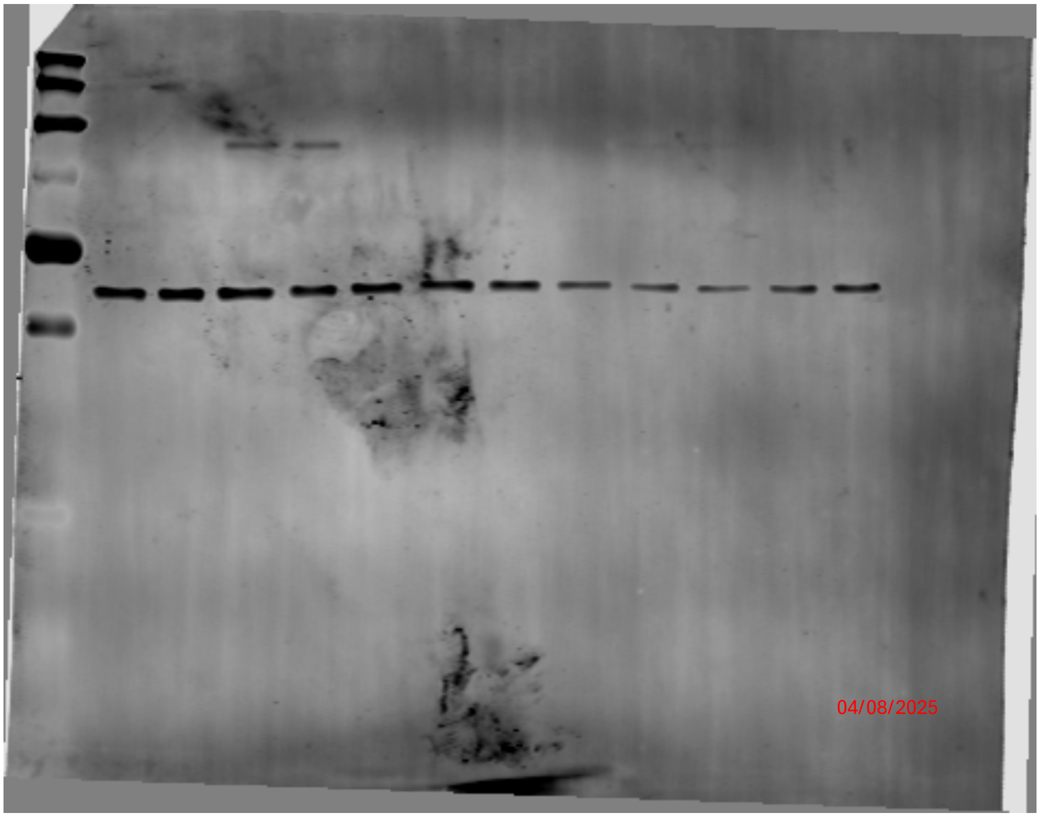
04/08/2025

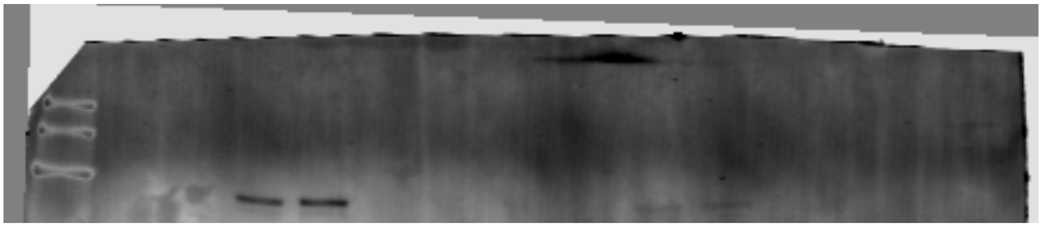

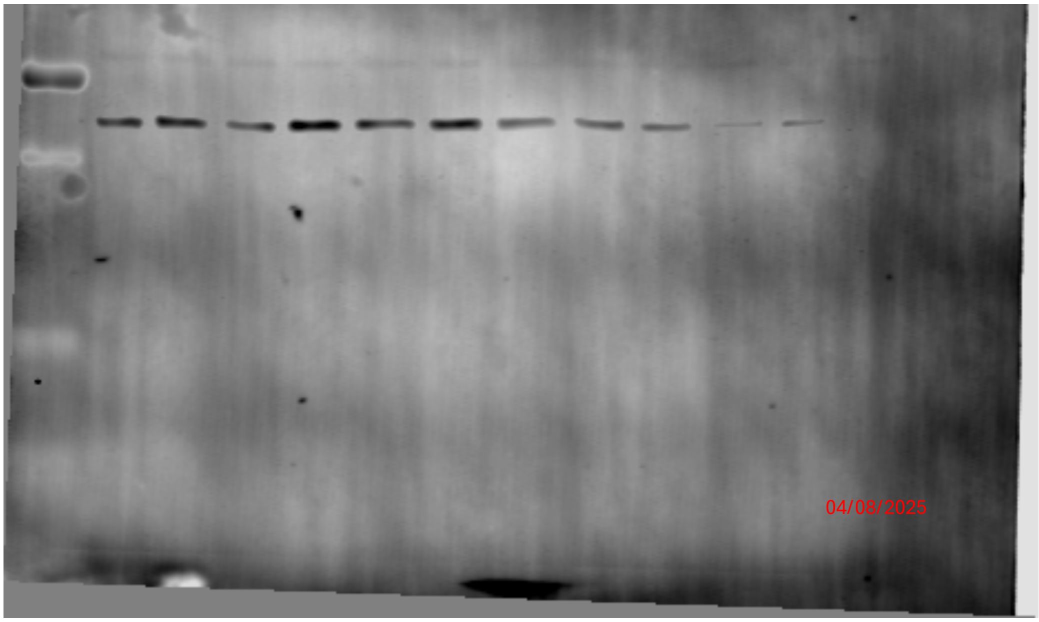
04/08/2025

VM-derived
poly(I:C)

plotted values in red, excluded outliers in gray

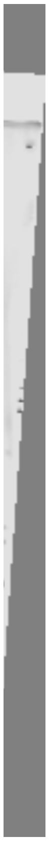

Quantification of bands with ImageStudioLite

	ReN VM			
	lipofectamine	polyIC	polyIC + drug	lipofectamine
pSTAT1	2.387695313	93.9995117	4.068847656	1.207519531
	2.264160156	83.7285156	2.379394531	2.451171875
actin	3704.489258	4573.19238	4786.487305	3940.726563
	4594.296875	4072.41992	4395.228516	4008.250977
normalized t(	0.000644541	0.02055446	0.00085007	0.000306421
	0.00049282	0.02055989	0.000541359	0.000611532
average	0.00056868	0.02055718	0.000695714	0.000458976
normalized t(	3.135357599	99.9867932	4.135147753	1.490576866
	2.397312237	100.013207	2.63342839	2.974783552
average	2.766334918	100	3.384288071	2.232680209

: annotations stay the same):

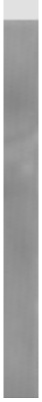

Quantification of bands with ImageStudioLite

	ReN VM			
	lipofectamine	polyIC	polyIC + drug	lipofectamine
pSTAT1	0.0001	98.921875	0.0001	0.0001
	0.0001	20.796875	0.0001	0.0001
actin	5410.712891	6841.91797	6143.923828	4068.681641
	6627.308594	6559.89844	5246.408203	3449.882813
normalized t(	1.84819E-08	0.01445821	1.62762E-08	2.4578E-08

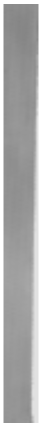

	1.50891E-08	0.0031703	1.90607E-08	2.89865E-08
average	1.67855E-08	0.00881426	1.76685E-08	2.67822E-08
normalized t(	0.000209681	164.032083	0.000184658	0.000278844
	0.00017119	35.9679171	0.000216248	0.000328859
average	0.000190435	100	0.000200453	0.000303851

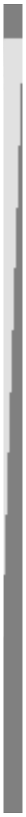

Quantification of bands with ImageStudioLite

	ReN VM			
	lipofectamine	polyIC	polyIC + drug	lipofectamine
pSTAT1	0.0001	172.589844	0.0001	15.92578125
	0.0001	111.238281	0.60546875	19.9
actin	3842.492188	3861.91602	3373.837891	2746
	3763.314453	3098.93555	3363.753906	2265.199219
normalized t(	2.60248E-08	0.04469021	2.96398E-08	0.005799629
	2.65723E-08	0.03589564	0.000179998	0.008785099
average	2.62985E-08	0.04029293	9.00138E-05	0.007292364
normalized t(	6.45889E-05	110.913292	7.35609E-05	14.39366525
	6.59479E-05	89.0867081	0.446723401	21.80308049
average	6.52684E-05	100	0.223398481	18.09837287

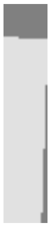

Quantification of bands with ImageStudioLite

	ReN VM			
	lipofectamine	polyIC	polyIC + drug	lipofectamine

pSTAT1	0.4921875	100.501953	0.0001	0.0001
	0.0001	106.369141	0.0001	0.0001
actin	4548.787109	3702.42578	4457.037109	3188.626953
	5113.740234	5730.40625	4970.167969	3188.148438
normalized t	0.000108202	0.02714489	2.24364E-08	3.13615E-08
	1.95552E-08	0.01856223	2.012E-08	3.13662E-08
average	5.41107E-05	0.02285356	2.12782E-08	3.13638E-08
normalized t	0.473457541	118.777508	9.81748E-05	0.000137228
	8.55672E-05	81.2224917	8.8039E-05	0.000137248
average	0.236771554	100	9.31069E-05	0.000137238

TYK2 KD	
polyIC	polyIC + drug
32.4208984	1.931152344
36.762207	0.602050781
3735.69141	4839.552734
5319.55664	5708.920898
0.00867869	0.000399035
0.00691077	0.000105458
0.00779473	0.000252247
42.2173108	1.941099612
33.6172863	0.51299793
37.9172986	1.227048771

TYK2 KD	
polyIC	polyIC + drug
0.0001	0.0001
0.0001	0.0001
3131.37891	2224.693359
3014.37891	2580.265625
3.1935E-08	4.495E-08

3.3174E-08	3.87557E-08
3.2555E-08	4.18529E-08
0.00036231	0.000509969
0.00037637	0.000439693
0.00036934	0.000474831

TYK2 KD	
polyIC	polyIC + drug
6.14257813	12.28125
16.6894531	7.236328125
1999.21875	2177.195313
1846.76172	2467.712891
0.00307249	0.005640858
0.00903714	0.002932403
0.00605482	0.00428663
7.62538117	13.99962383
22.4286135	7.277711015
15.0269973	10.63866742

TYK2 KD	
polyIC	polyIC + drug

6.69726563	0.0001
7.57421875	0.0001
3040.09375	2411.074219
2318.89258	2198.865234
0.00220298	4.14753E-08
0.00326631	4.5478E-08
0.00273464	4.34766E-08
9.63954683	0.000181483
14.2923382	0.000198997
11.9659425	0.00019024

Figure 7b: CXCL10 concentration in pg/ml in the media of ReN VM-derived neural cells treate
transfected with po

plotted values in red

[pg/ml]	Baricitinib			Deucravacitini	
polyIC	9027.92885	8718.726686	9811.787953	12077.1173	15047.7342
0.01 uM	5128.26974	4875.177858	4930.476063	1127.73462	1320.85001
0.1 uM	2172.97745	2695.7074	2551.903182	361.77613	438.14625
1 uM	239.248231	185.634935	178.2803213	130.524166	140.139684
10 uM	85.6389645	69.19428285	64.33703669	41.0535181	61.478782
lipo only	7.62757992	11.82985744	9.023394467	0.65961084	0.57880096

ed with different concentrations of baricitinib, ruxolitinib, or deucravacitinib and
oly(I:C)

b	Ruxolitinib		
12242.9239	8939.98755	10328.3598	9973.83468
1307.30855	3768.245487	4532.21456	4055.83834
603.94975	2500.114851	2337.88405	1934.12713
140.162295	125.4080566	215.099718	198.563121
52.8483153	42.79401654	41.0009682	44.2244003
0.61976396	3.763804562	8.69456595	6.46125012

Figure 7c: CXCL10 concentration in the media of ReN VM-derived neural cells treated with 1 µ
any additional drug (ctrl), or with additional 10 µM baricit

plotted values in red

	no MG-132				
biological replicate	Ctrl	Baricitinib	Deucravacitinib	Ruxolitinib	Ctrl
1	0.2763223	0.13688036	0.1866614	0.2005524	0.26945633
2	0.28037566	0.17480617	0.1524709	0.22863543	0.21758712
3	0.17777164	0.18362189	0.16824945	0.18661678	0.31378218
4	0.15391376	0.11401183	0.09378061	0.11551395	1.32253255
5	0.12122628	0.1095167	0.13029322	0.07679445	1.60007807
6	0.11650448	0.14447801	0.06702064	0.11785023	1.51274823
7	0.22186642	0.14028268	0.11267423	0.14986631	0.23421602
8	0.15112454	0.17755614	0.13429097	0.14991934	0.20279148
9	0.13185677	0.09999455	0.06714849	0.14016377	0.27933943
Average	0.18121798	0.14234981	0.123621101	0.15176807	0.66139238

M MG-132 for the translocation of TDP-43 from the nucleus to the cytoplasm without
:inib, ruxolitinib, or deucravacitinib treatment.

1 µM MG-132		
Baricitinib	Deucravacitinib	Ruxolitinib
0.32999309	0.1804644	0.23782578
0.31986607	0.16206793	0.13737287
0.21707892	0.10460311	0.19853236
0.78154123	0.28013124	0.5239646
0.61102875	0.24841182	0.7782966
0.46285123	0.314764	0.80308265
0.16806318	0.18451078	0.16268644
0.28029826	0.12434007	0.20660407
0.17724487	0.11951086	0.12873985
0.37199618	0.190978246	0.35301169

Supplementary Figure 1c: Proportion of cells that stained for cdsRNA, p

Number of Cells	Control	Alzheimer's Disease
dsRNA/pTDP-43-negative	57	12
dsRNA-positive	24	30
pTDP-43-positive	2	7
dsRNA/pTDP-43-positive	40	84
total	123	133

TDP-43, or both - underlying absolute numbers

Supplementary Figure 2b: Proportion of pPKR-positive cells that co-stained for cdsRNA,

	pPKR-positive	pPKR/dsRNA-positive	pPKR/TDP-43-positive	pPKR/dsRNA/TDP-43-positive	Total
Number of cells	15	11	8	33	67

pTDP-43, or both in a randomly selected site - underlying absolute numbers

Supplementary Figure 8a: Detected CellTiter-Glo luminescence signal - Cell survival of ReN VM-deri
at different concentrations and afterwards transfected with poly(I:C) or

plotted values in red

experiment 1

biological replicate	poly(I:C) 10 µM	1 µM	0.1 µM	0.01 µM
1	24073	13993	10363	9498
2	22889	13048	10463	9518
3	22928	12808	11882	10458
4				
5				
6				
average	23296.6667	13283	10902.6667	9824.66667

normalizing signal to average ctrl *100:

Percentage cell survival

biological replicate	poly(I:C) 10 µM	1 µM	0.1 µM	0.01 µM
1	137.998968	80.2151606	59.4061109	54.4474806
2	131.211664	74.7979286	59.9793629	54.562131
3	131.435232	73.4221237	68.1138096	59.9507003
4				
5				
6				
average	133.548621	76.145071	62.4997611	56.3201039

experiment 2

biological replicate	poly(I:C) 10 µM	1 µM	0.1 µM	0.01 µM
7	18664	15835	11740	9057
8	18630	15680	11698	9761
9	18787	15327	9726	8652
10				
11				
12				
average	18693.6667	15614	11054.6667	9156.66667

normalizing signal to average ctrl *100:

Percentage cell survival

biological replicate	poly(I:C) 10 µM	1 µM	0.1 µM	0.01 µM
7	124.563687	105.682918	78.3528548	60.4464911

8	124.33677	104.648447	78.0725465	65.1449928
9	125.38459	102.292522	64.9114025	57.7435179
10				
11				
12				
average	124.761682	104.207962	73.7789346	61.1116673

experiment 3

	poly(I:C)			
biological replicate	10 µM	1 µM	0.1 µM	0.01 µM
13	4409	3604	3074	2100
14	3756	3561	2338	2060
15	3894	2916	2206	2129
16				
17				
18				
average	4019.66667	3360.33333	2539.33333	2096.33333

normalizing signal to average ctrl *100:

Percentage cell survival

	poly(I:C)			
biological replicate	10 µM	1 µM	0.1 µM	0.01 µM
13	125.001181	102.17833	87.1521051	59.5378727
14	106.487738	100.959221	66.2854983	58.403818
15	110.400227	82.672589	62.5431177	60.3600624
16				
17				
18				
average	113.963049	95.2700468	71.9935737	59.4339177

ved neural cells pre-treated with baricitinib
lipofectamine

lipofectamine	0 µM
18129	5187
17098	6113
18162	4581
17944	4881
16530	4197
16803	5260
17444.33333	5036.5

lipofectamine	0 µM
103.9248658	29.7345843
98.01463704	35.0428984
104.1140389	26.2606768
102.8643495	27.980433
94.75856534	24.0593889
96.32354346	30.1530583
100	28.8718399

lipofectamine	0 µM
14974	5333
15459	4259
15531	2914
13740	3725
16415	3686
13782	5756
14983.5	4278.83333

lipofectamine	0 µM
99.93659692	35.5924851

103.1734908	28.4246004
103.6540194	19.4480595
91.70087096	24.8606801
109.5538426	24.6003938
91.9811793	38.4155905
100	28.5569682

lipofectamine	0 μM
2915	1180
3320	1150
3446	1237
4039	1253
3583	1297
3860	1343
3527.166667	1243.33333

lipofectamine	0 μM
82.64423758	33.4546142
94.12654161	32.6040731
97.69881397	35.0706422
114.5111752	35.524264
101.5829514	36.7717242
109.4362803	38.0758872
100	35.2502008

Supplementary Figure 8b: Image and quantification of Western blot of pSTAT1Y701 in ReN VM-

anti-pSTAT1
plotted values in red

shown in figure:

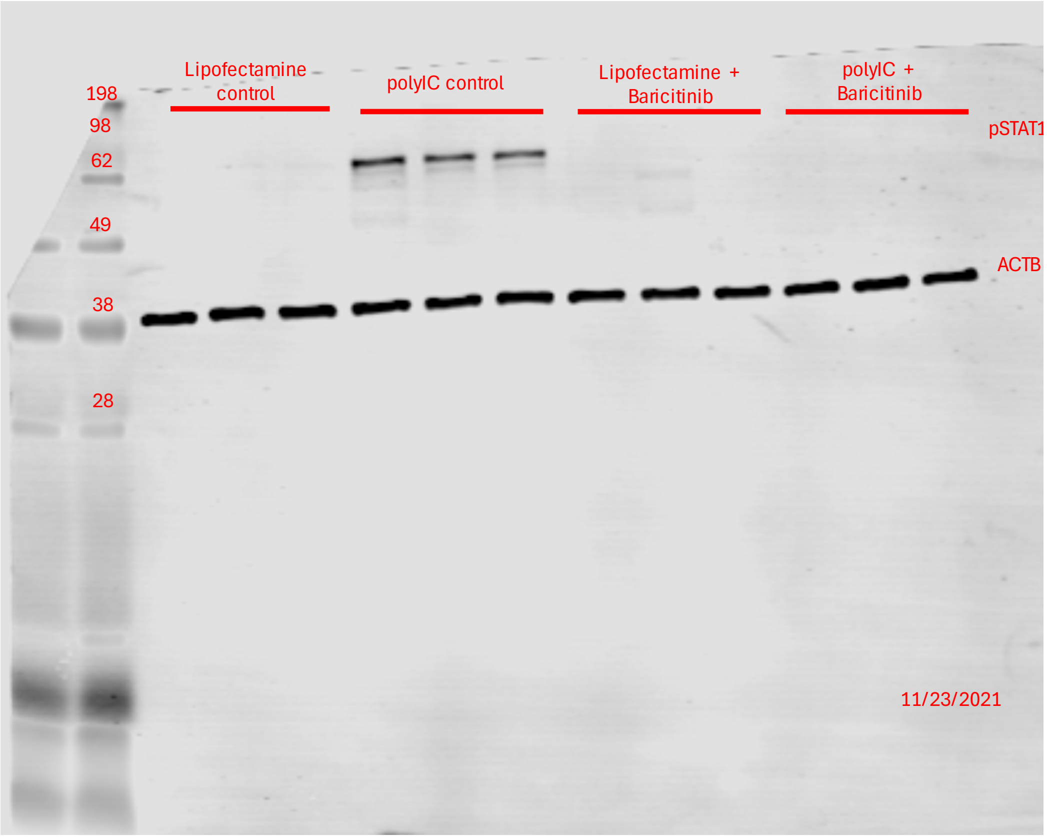

additional blots for quantification (all blots have the same layout and protein ladders, hence the

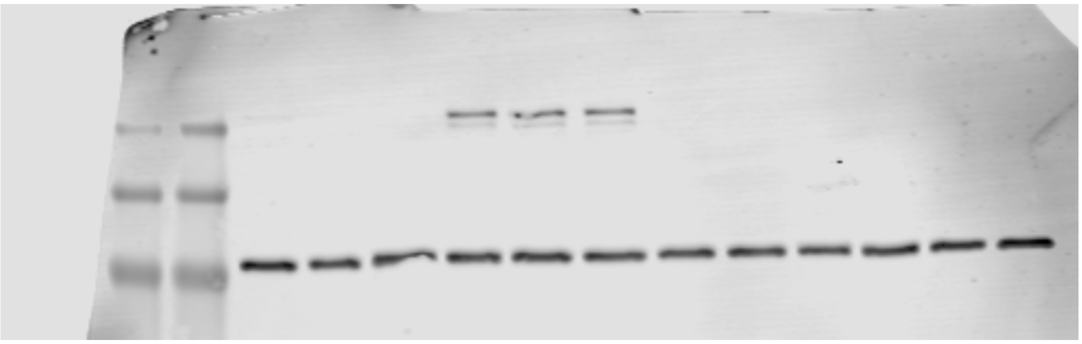

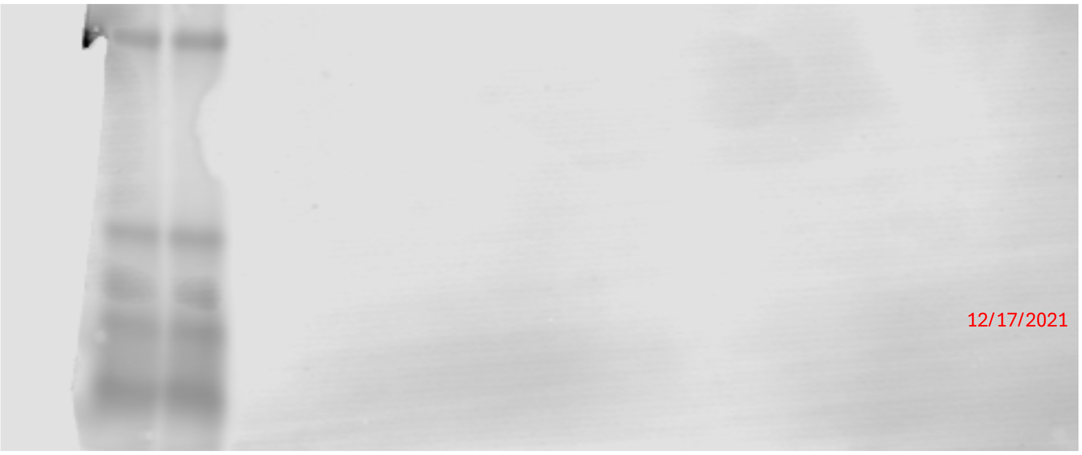
12/17/2021

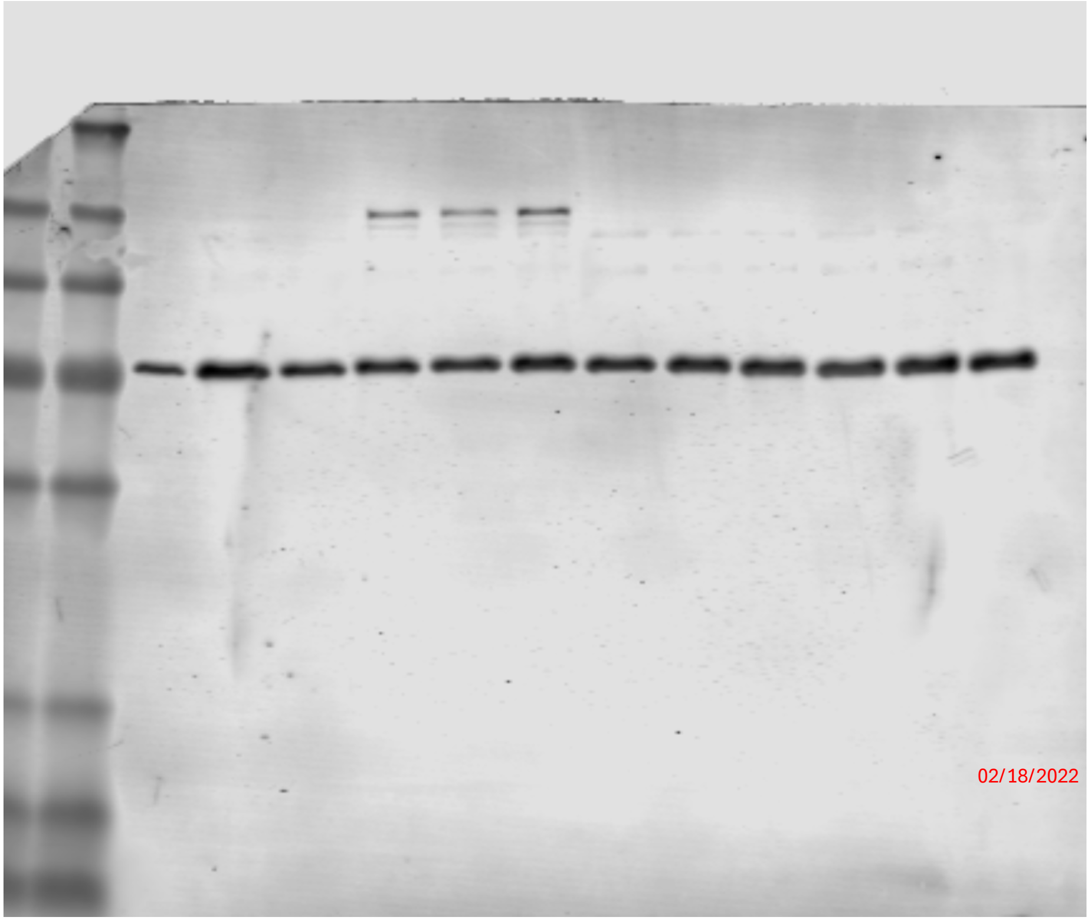
02/18/2022

derived neural cells 24 hours after treatment with 10 µM baricitinib and transfection with poly(I:C
(ACTB).

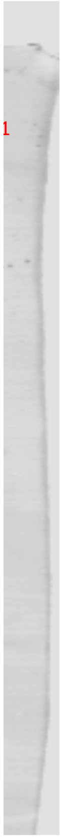

Quantification of bands with ImageStudioLite

	Lipofectamine ctrl	poly(I:C) control
pSTAT1	3.340820313	204.9628906
	1.75	205.4238281
	1.734375	210.2167969
Actin	15215.99219	11323.00195
	10203.64844	12762.21289
	10346.0293	11185.24023
Normalized t	0.00021956	0.018101462
	0.000171507	0.016096255
	0.000167637	0.018794124
Average	0.000186235	0.017663947
Normalized t	1.242982708	102.4768815
*100	0.970945398	91.124903
	0.949033508	106.3982155
Avergae	1.054320538	100

: annotations stay the same):

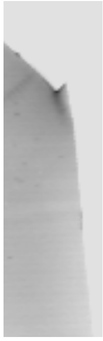

Quantification of bands with ImageStudioLite

	Lipofectamine ctrl	poly(I:C) control
pSTAT1	6.098632813	248.8603516
	5.540039063	202.7441406
	7.873046875	199.7011719
Actin	22694.18945	20576.0625
	22251.22852	17900.82031

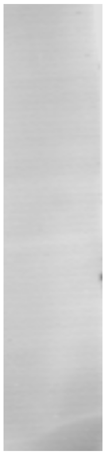

	22208.71289	19872.91016
Normalized t	0.000268731	0.012094654
	0.000248977	0.011325969
	0.000354503	0.010048914
Average	0.000290737	0.011156512
Normalized t *100	2.408736843	108.4089119
	2.231672072	101.5189049
	3.177539756	90.07218326
Avergae	2.60598289	100

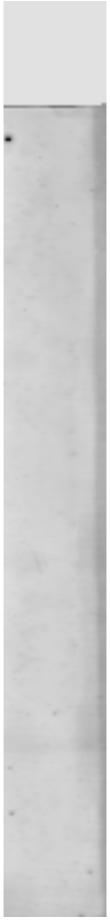

Quantification of bands with ImageStudioLite

	Lipofectamine ctrl	poly(I:C) control
pSTAT1	2.521484375	106.8339844
	5.390625	81.3359375
	2.31640625	134.0878906
Actin	6006.568359	12329.31641
	24188.15234	11393.16211
	11411.72852	16768.33594
Normalized t	0.000419788	0.008665037
	0.000222862	0.007139013
	0.000202985	0.007996494
Average	0.000281878	0.007933515
Normalized t *100	5.291322421	109.2206599
	2.80912325	89.98550708
	2.558572131	100.793833
Avergae	3.553005934	100

C) normalized to the housekeeping protein beta actin

drug + lipo	drug + poly(I:C)
2.671875	10.90039063
8.13671875	4.640625
16.88769531	10.05859375
9869.855469	12117.44922
10598.07422	13180.83984
8538.125	18902.05469
0.000270711	0.000899561
0.000767754	0.000352074
0.001977916	0.000532143
0.00100546	0.000594593
1.532560363	5.092641432
4.346449058	1.993175949
11.19747553	3.012593249
5.69216165	3.366136877

drug + lipo	drug + poly(I:C)
1.23828125	3.564453125
1.331054688	2.361328125
7.283203125	0.584960938
17589.0625	18674.34375
16903.36523	20521.72266

16873.95117	18049.48828
7.04006E-05	0.000190874
7.8745E-05	0.000115065
0.000431624	3.24087E-05
0.00019359	0.000112783
0.631027303	1.710878247
0.705820502	1.031368995
3.868808023	0.290491559
1.735218609	1.010912934

drug + lipo	drug + poly(I:C)
0.815429688	9.56640625
9.818359375	18.48535156
1.911132813	7.31640625
14022.20898	11255.65625
14660.73633	18512.2832
13765.44141	19564.09375
5.81527E-05	0.00084992
0.000669704	0.000998545
0.000138836	0.000373971
0.000288898	0.000740812
0.733000805	10.71303113
8.441458885	12.58641645
1.749988099	4.713814035
3.641482596	9.337753873

Supplementary Figure 8c: Detected CellTiter-Glo luminescence signal - Cell survival of ReN VM-d
ruxolitinib at different concentrations and afterwards transfected with poly(I:C)

plotted values in red

experiment 1

	poly(I:C)			
biological replicate	10 µM	1 µM	0.1 µM	0.01 µM
1	20979	12771	10025	9001
2	19320	12471	10296	9806
3	19426	12167	11042	9133
4				
5				
6				
average	19908.3333	12469.6667	10454.3333	9313.33333

normalizing signal to average ctrl *100:

Percentage cell survival

	poly(I:C)			
biological replicate	10 µM	1 µM	0.1 µM	0.01 µM
1	121.341881	73.8670651	57.9842869	52.0615029
2	111.746277	72.1318745	59.5517424	56.7175977
3	112.359377	70.373548	63.8665831	52.8249867
4				
5				
6				
average	115.149178	72.1241625	60.4675375	53.8680291

experiment 2

	poly(I:C)			
biological replicate	10 µM	1 µM	0.1 µM	0.01 µM
7	19196	14578	8392	9277
8	18300	14218	11470	10204
9	18256	14421	10662	7948
10				
11				
12				
average	18584	14405.6667	10174.6667	9143

normalizing signal to average ctrl *100:

Percentage cell survival

	poly(I:C)			
biological replicate	10 µM	1 µM	0.1 µM	0.01 µM
7	135.694341	103.050224	59.3220938	65.5780582

8	129.360619	100.505425	81.0801258	72.1309158
9	129.049588	101.940409	75.3684657	56.1835083
10				
11				
12				
average	131.368183	101.83202	71.9235618	64.6308274

experiment 3

	poly(I:C)			
biological replicate	10 µM	1 µM	0.1 µM	0.01 µM
13	4394	3430	2754	2343
14	4198	3894	3116	2688
15	5424	3383	2750	2588
16				
17				
18				
average	4672	3569	2873.33333	2539.66667

normalizing signal to average ctrl *100:

Percentage cell survival

	poly(I:C)			
biological replicate	10 µM	1 µM	0.1 µM	0.01 µM
13	101.151013	78.9594843	63.3977901	53.9364641
14	96.6390424	89.640884	71.7311234	61.878453
15	124.861878	77.8775322	63.305709	59.5764273
16				
17				
18				
average	107.550645	82.1593002	66.1448742	58.4637815

erived neural cells pre-treated with
or lipofectamine

lipofectamine	0 μM
18822	6312
17113	6535
16755	5941
15388	5761
18663	5662
16994	7615
17289.1667	6304.33333

lipofectamine	0 μM
108.86586	36.5084109
98.9810575	37.7982359
96.9103967	34.3625584
89.0037114	33.3214441
107.946209	32.7488312
98.2927652	44.0449222
100	36.4640671

lipofectamine	0 μM
13490	3817
15197	5107
14209	4005
13849	4951
14444	3786
13690	4164
14146.5	4305

lipofectamine	0 μM
95.3592761	26.981939

107.425865	36.1008023
100.441805	28.3108896
97.8970063	34.9980561
102.102994	26.7628035
96.7730534	29.4348425
100	30.4315555

lipofectamine	0 μM
4052	1063
4913	1404
3493	1104
5206	1056
4719	1577
3681	1418
4344	1270.33333

lipofectamine	0 μM
93.2780847	24.4705341
113.098527	32.320442
80.4097606	25.4143646
119.843462	24.3093923
108.632597	36.3029466
84.7375691	32.6427256
100	29.2434009

Supplementary Figure 8d: Image and quantification of Western blot of pSTAT1Y701 in ReN VM-c

anti-pSTAT1
plotted values in red

shown in figure:

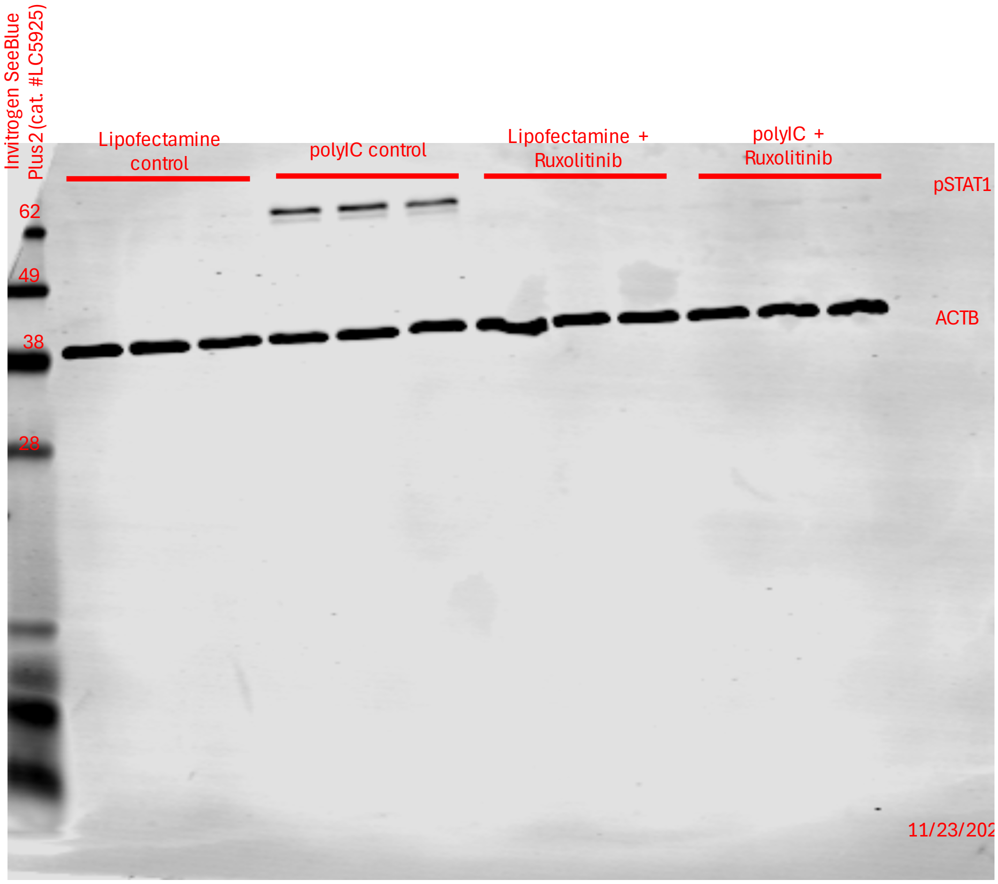

additional blots for quantification (all blots have the same layout and protein ladd

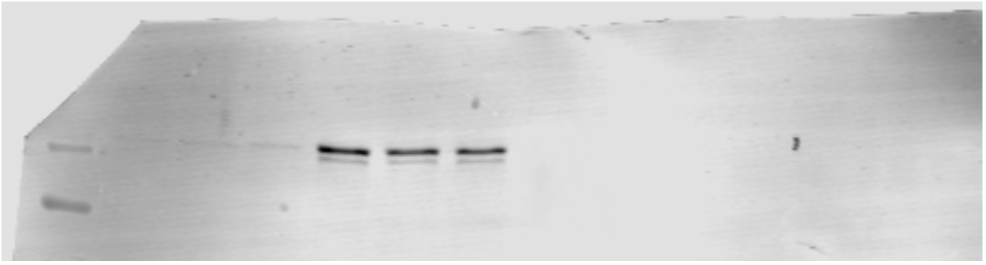

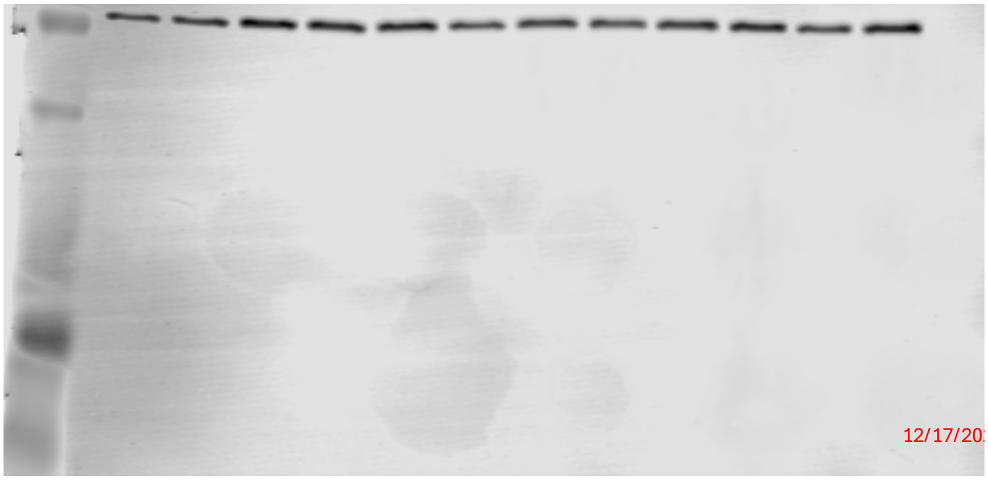
12/17/20

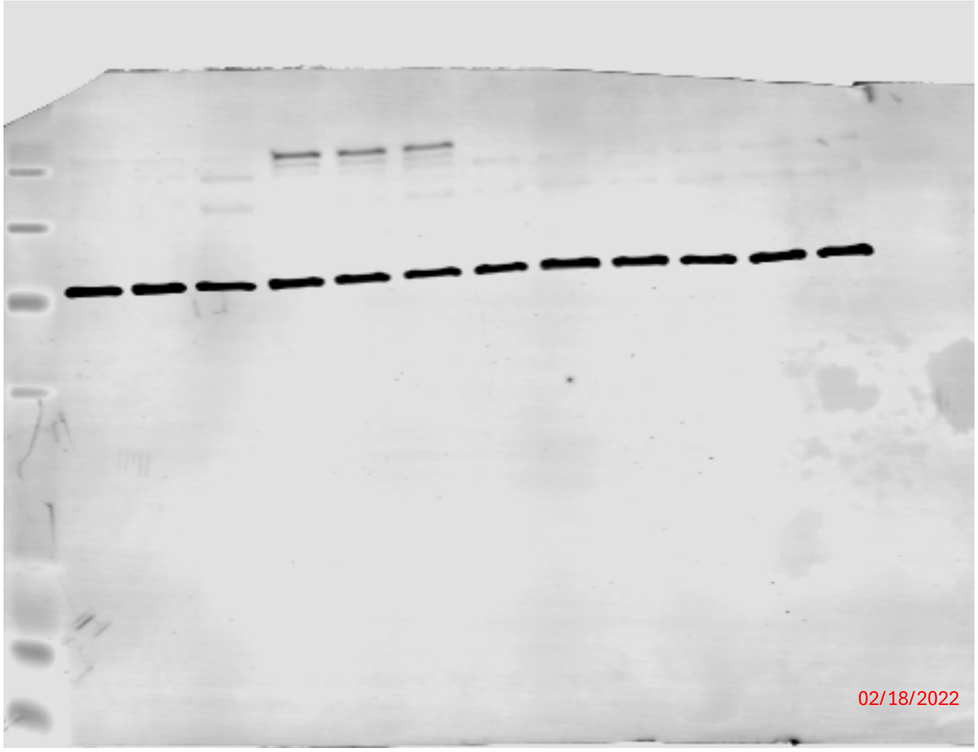
02/18/2022

derived neural cells 24 hours after treatment with 10 µM ruxolitinib and transfection with poly(I:C) normalize actin (ACTB).

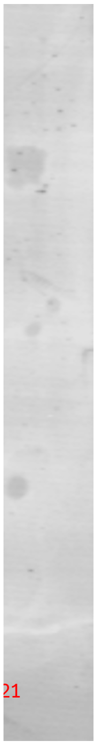

Quantification of bands with ImageStudioLite

	Lipofectamine ctrl	poly(I:C) control
pSTAT1	6.1328125	262.9667969
	6.744140625	179.5566406
	15.23242188	179.5292969
Actin	9390.099609	13469.04297
	10654.5332	13603.57031
	15668.875	7484.753906
Normalized t	0.000653115	0.019523792
	0.000632983	0.013199229
	0.000972145	0.023985999
Average	0.000752748	0.018903007
Normalized t *100	3.455083923	103.2840575
	3.348584812	69.82608274
	5.142807355	126.8898597
Avergae	3.982158697	100

ers, hence the annotations stay the same):

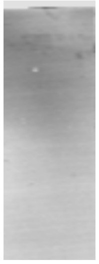

Quantification of bands with ImageStudioLite

	Lipofectamine ctrl	poly(I:C) control
pSTAT1	2.56640625	239.4501953
	0.680664063	243.0283203
	1.6953125	199.7138672
Actin	25454.72754	8857.62793

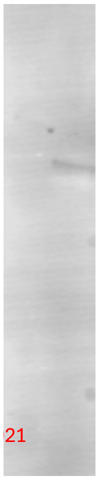

	17250.44727	11346.90723
	9529.71875	16883.09961
Normalized t	0.000100822	0.027033219
	3.94578E-05	0.021418023
	0.000177897	0.011829218
Average	0.000106059	0.020093487
Normalized t *100	0.501766483	134.5372228
	0.196370912	106.5918691
	0.885348747	58.87090809
Avergae	0.527828714	100

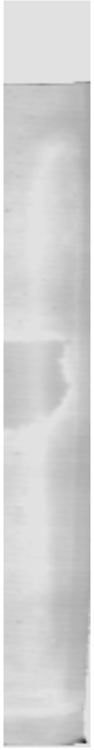

Quantification of bands with ImageStudioLite

	Lipofectamine ctrl	poly(I:C) control
pSTAT1	7.181640625	196.9160156
	6.55078125	173.7011719
	30.45703125	142.1132813
Actin	26627.86523	13560.33203
	25374.20313	12181.45313
	16799.17969	12124.375
Normalized t	0.000269704	0.014521474
	0.000258167	0.014259479
	0.001813007	0.011721287
Average	0.000780293	0.013500747
Normalized t *100	1.99769658	107.5605276
	1.912242176	105.6199257
	13.428939	86.81954673
Avergae	5.779625918	100

ed to the housekeeping protein beta

drug + lipo	drug + poly(I:C)
1.9765625	0.2265625
1.673828125	2.244140625
0.134765625	0.357421875
10209.23438	12912.8457
8176.966797	8120.746094
11657.74219	14685.14063
0.000193605	1.75455E-05
0.0002047	0.000276347
1.15602E-05	2.4339E-05
0.000136622	0.000106077
1.024204054	0.092818635
1.082898469	1.461918792
0.061155257	0.12875738
0.722752593	0.561164936

drug + lipo	drug + poly(I:C)
2.305664063	5.061523438
0.012695313	6.453125
2.8671875	8.490234375
22811.45605	17699.94043

19985.01074	19958.99609
21051.3457	23597.87305
0.000101075	0.000285963
6.35242E-07	0.000323319
0.0001362	0.000359788
7.93033E-05	0.000323023
0.503022857	1.42316136
0.003161431	1.609074235
0.677830221	1.790570872
0.394671503	1.607602156

drug + lipo	drug + poly(I:C)
2.478515625	3.875
1.71875	14.69140625
8.7890625	6.87890625
11865.32227	16219.23242
15985.62695	22085
16034.23047	22224.05469
0.000208887	0.000238914
0.000107518	0.000665221
0.000548144	0.000309525
0.000288183	0.000404553
1.547228051	1.769634665
0.796388985	4.927290548
4.060099143	2.292652859
2.13457206	2.996526024

Supplementary Figure 8e+f: Image and quantification of Western blot of pSTAT1Y701 in ReN
no

anti-pSTAT1
plotted values in red

shown in figure:

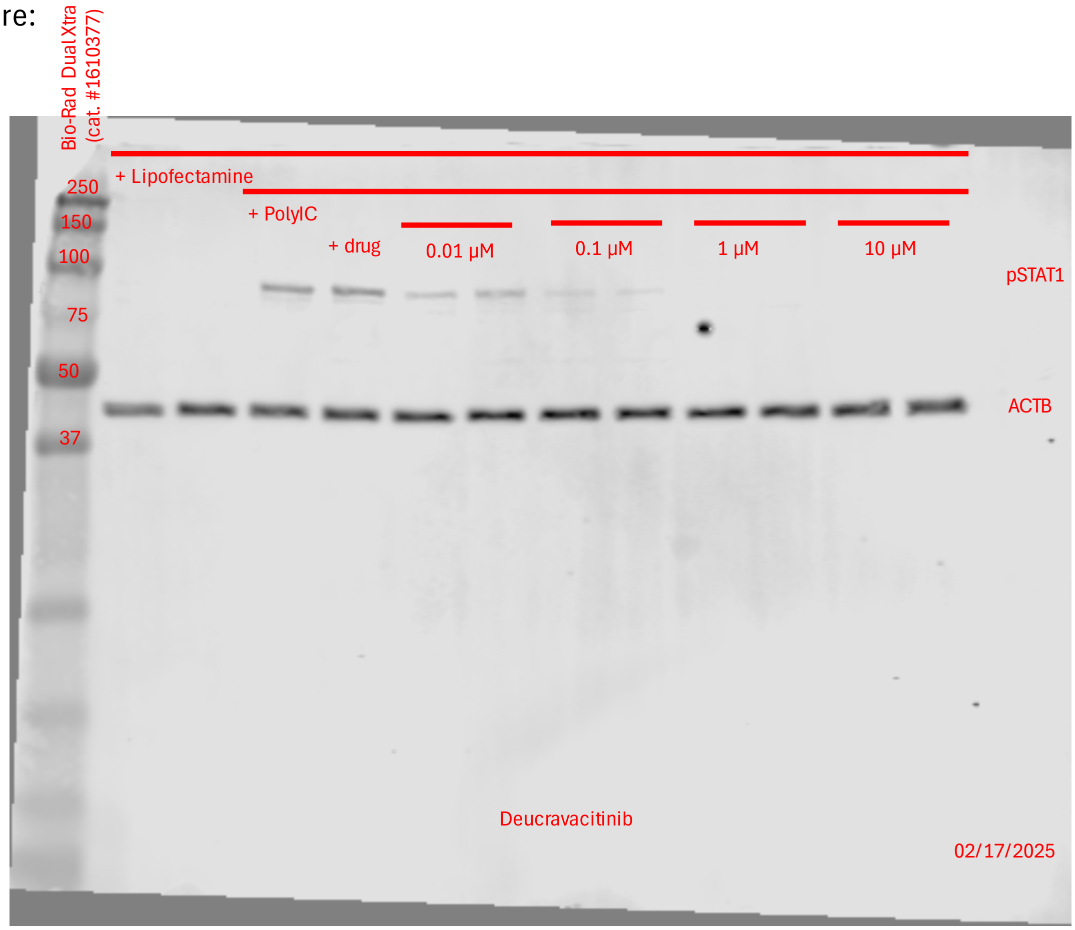

additional blots for quantification (all blots have the same layout and protein ladders, hence the a

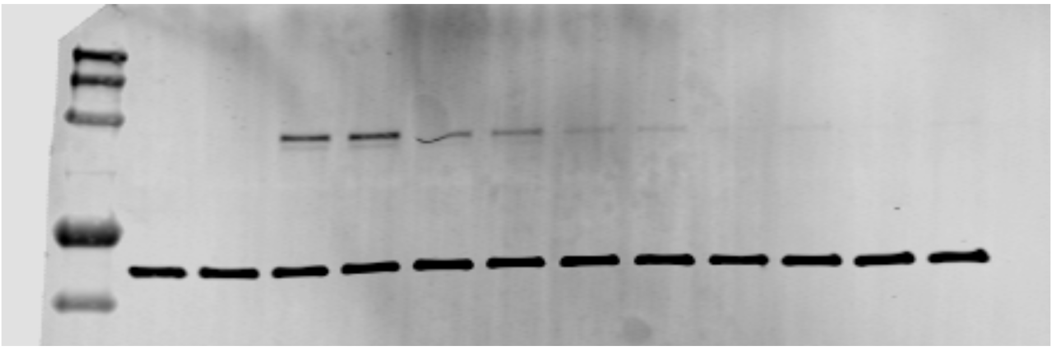

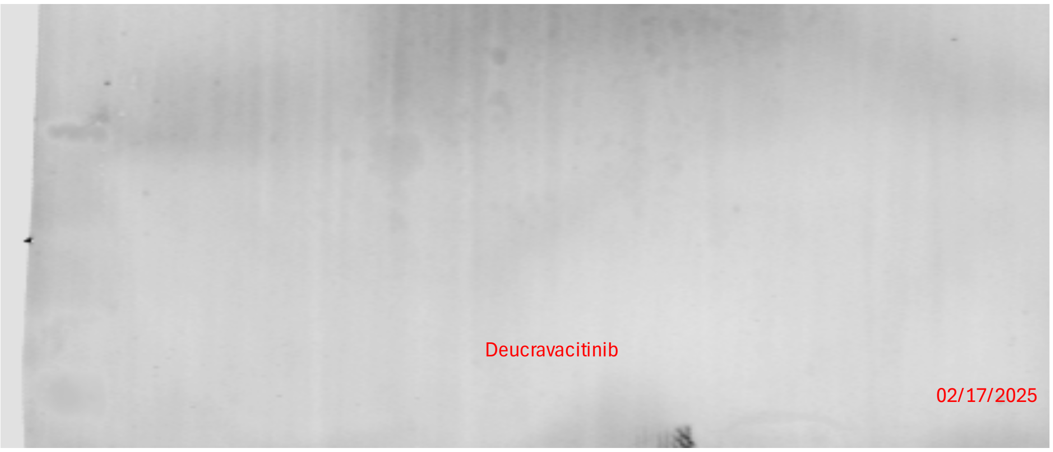
Deucravacitinib
02/17/2025

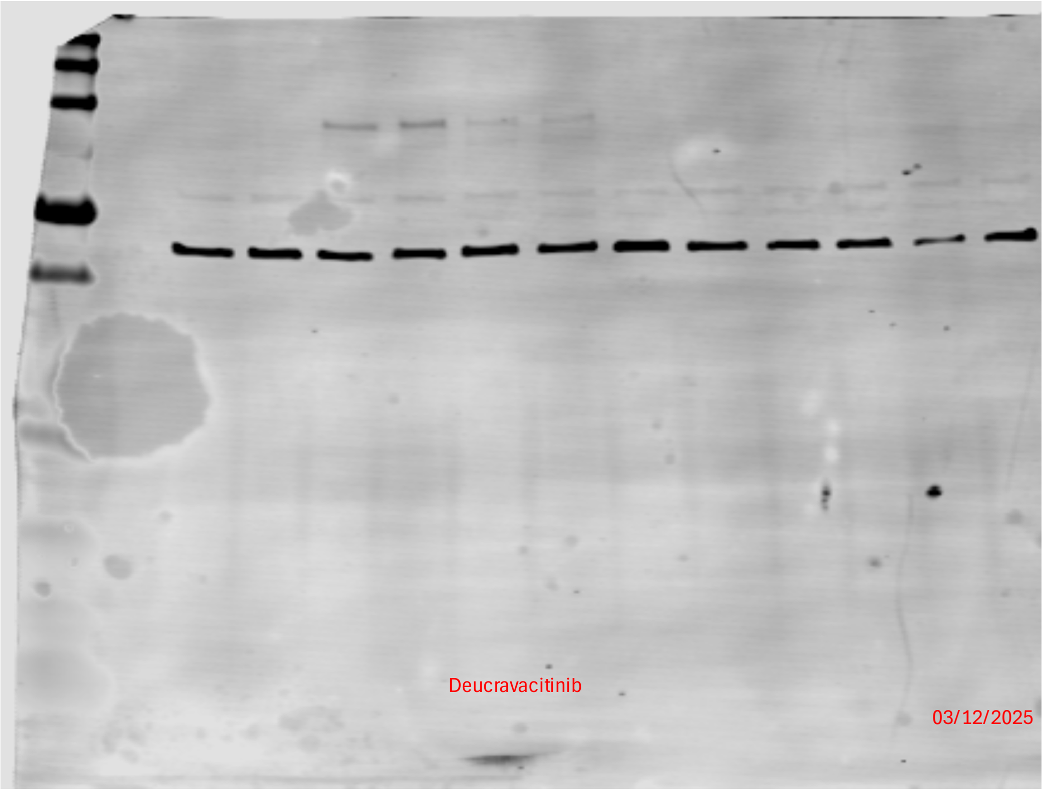
Deucravacitinib
03/12/2025

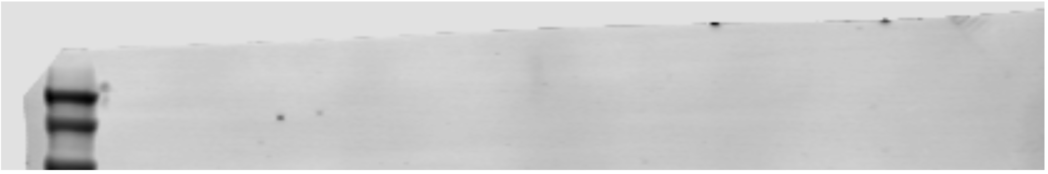

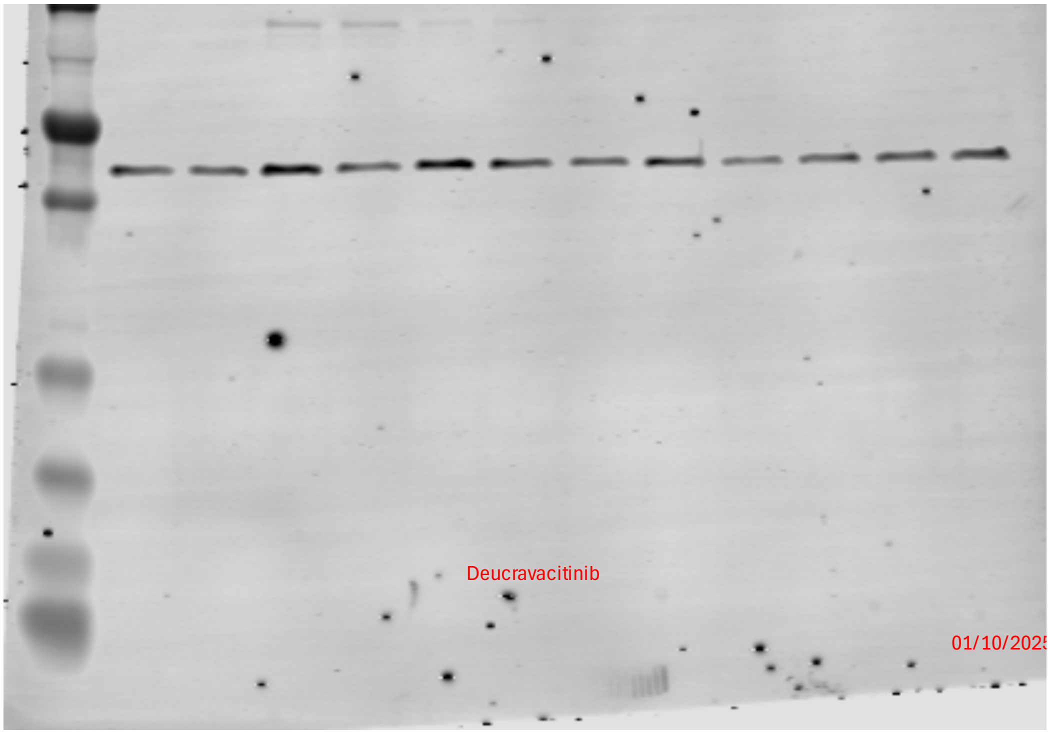
Deucravacitinib
01/10/2025

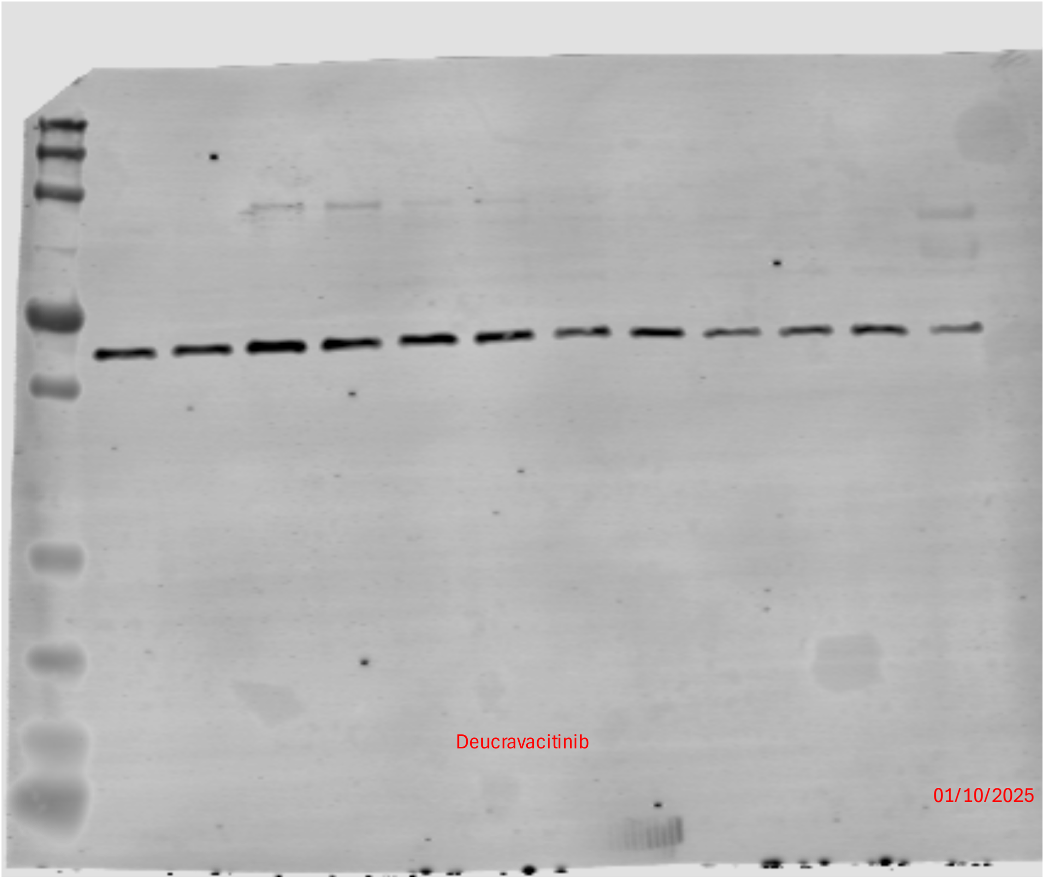
Deucravacitinib
01/10/2025

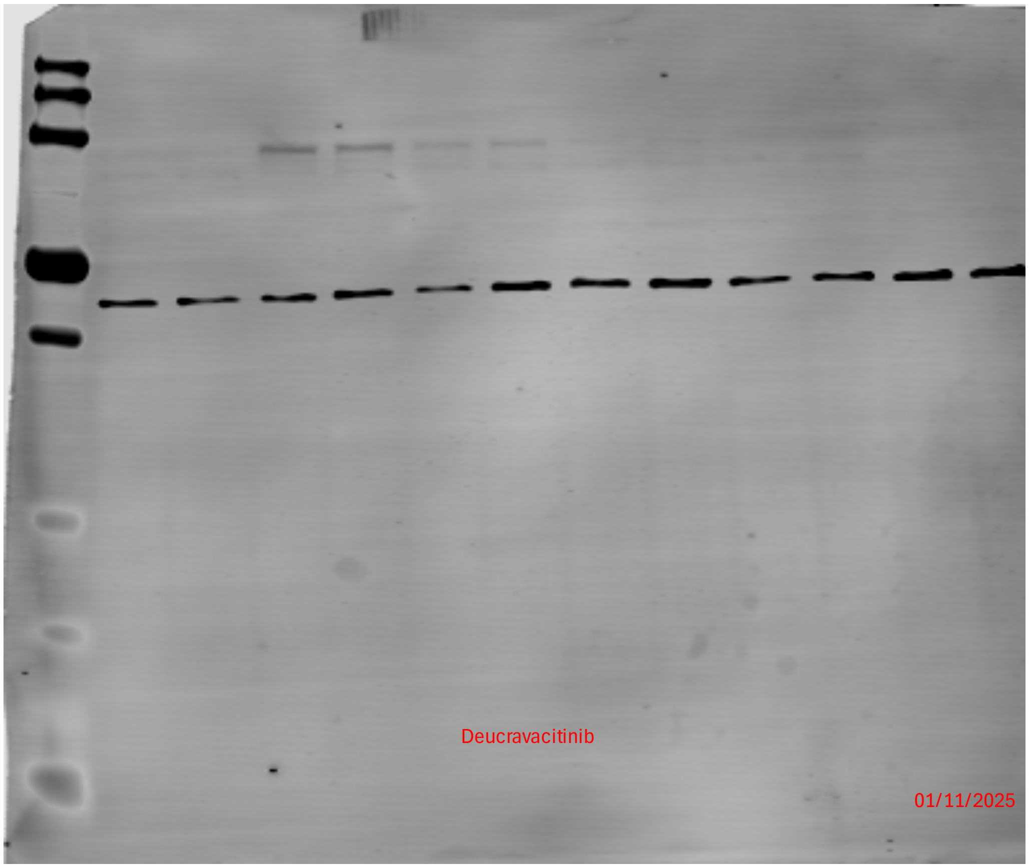

Deucravacitinib

Quantification of bands with ImageStudioLite

Blot 1

	ctrl	polyIC	0.01 µM	0.1 µM	1 µM
pSTAT1	2.95800781	77.6040039	20.4677734	4.62353516	1.66113281
	2.10351563	66.9672852	20.9477539	8.6796875	9.33105469
actin	2954.55566	2758.41016	2040.95215	3030.27441	2839.71484
	2649.55957	3285.35449	4070.62305	4334.90234	3399.75781
average	2802.05762	3021.88232	3055.7876	3682.58838	3119.73633
normalized to actin	0.00100117	0.0281336	0.01002854	0.00152578	0.00058496
	0.00079391	0.02038358	0.00514608	0.00200228	0.00274462
average	0.00089754	0.02425859	0.00758731	0.00176403	0.00166479
normalized to polyIC *100	4.12706778	115.973762	41.3401683	6.28965272	2.41137129
	3.2727024	84.0262375	21.2134369	8.25390056	11.3140271
average	3.69988509	100	31.2768026	7.27177664	6.86269921

Blot 2

	ctrl	polyIC	0.01 µM	0.1 µM	1 µM
pSTAT1	3.17382813	38.7089844	4.50195313	2.61328125	1.06201172
	0.84716797	29.3012695	9.60400391	1.8671875	2.13134766
actin	6678.90234	11170.8818	7760.5166	4254.33691	3252.60742
	6005.76563	7699.96875	6877.42383	5953.98047	3702.66797
average	6342.33398	9435.42529	7318.97021	5104.15869	3477.6377
normalized to actin	0.0004752	0.00346517	0.00058011	0.00061426	0.00032651
	0.00014106	0.00380538	0.00139645	0.0003136	0.00057562
average	0.00030813	0.00363527	0.00098828	0.00046393	0.00045107
normalized to polyIC *100	13.071982	95.3207495	15.9578165	16.8973034	8.98174643
	3.88029082	104.679251	38.4140121	8.62667934	15.8344381
average	8.4761364	100	27.1859143	12.7619914	12.4080923

Blot 3

	ctrl	polyIC	0.01 µM	0.1 µM	1 µM
pSTAT1	1.63248698	37.9446615	2.73307292	1.109375	0.07324219
	0.74283854	31.0289714	4.36946615	2.15527344	0.55696615
actin	2407.39583	4136.77279	4071.17708	1855.5612	1436.9681
	2026.13542	2083.03385	2663.43815	2609.60872	2061.95378
average	2216.76563	3109.90332	3367.30762	2232.58496	1749.46094
normalized to actin	0.00067811	0.00917253	0.00067132	0.00059786	5.097E-05
	0.00036663	0.01489605	0.00164054	0.0008259	0.00027012
average	0.00052237	0.01203429	0.00115593	0.00071188	0.00016054
normalized to polyIC *100	5.63484327	76.2199455	5.57841532	4.96801286	0.42353938
	3.04653085	123.780054	13.6321822	6.86288308	2.24455108
average	4.34068706	100	9.60529876	5.91544797	1.33404523

Blot 4

	ctrl	polyIC	0.01 µM	0.1 µM	1 µM
pSTAT1	1.703125	122.484375	43.8203125	24.2304688	16.4609375
	20.9296875	115.789063	51.9414063	29.0664063	19.453125
actin	4133.63477	3669.07031	4951.08984	7296.09766	4031.26172
	4849.80078	3921.01563	4266.99219	4467.70703	4132.04688
average	4491.71777	3795.04297	4609.04102	5881.90234	4081.6543
normalized to actin	0.00041202	0.03338295	0.00885064	0.00332102	0.00408332
	0.00431558	0.02953038	0.01217284	0.00650589	0.00470787
average	0.0023638	0.03145666	0.01051174	0.00491345	0.00439559
normalized to polyIC *100	1.30979042	106.123617	28.1359801	10.5574386	12.980785
	13.7191192	93.8763835	38.6971732	20.6820716	14.9661993

average	7.51445482	100	33.4165767	15.6197551	13.9734921

Blot 5

	ctrl	polyIC	0.01 µM	0.1 µM	1 µM
pSTAT1	0.26708984	103.47168	45.4907227	16.4663086	5.16015625
	0.66601563	113.537109	43.972168	16.6894531	7.92675781
actin	7500.88574	9410.4248	12309.3535	11089.498	8562.47266
	7831.83887	10587.9355	10654.9141	10995.8828	10276.1465
average	7666.3623	9999.18018	11482.1338	11042.6904	9419.30957
normalized to actin	3.5608E-05	0.01099543	0.00369562	0.00148486	0.00060265
	8.5039E-05	0.01072325	0.00412694	0.00151779	0.00077137
average	6.0324E-05	0.01085934	0.00391128	0.00150132	0.00068701
normalized to polyIC *100	0.32789992	101.2532	34.031734	13.6735363	5.54958032
	0.78309989	98.7468001	38.0035716	13.9768238	7.10332671
average	0.5554999	100	36.0176528	13.8251801	6.32645351

Blot 6

	ctrl	polyIC	0.01 µM	0.1 µM	1 µM
pSTAT1	1.29508464	101.558594	37.7519531	16.3186849	1.1023763
	1.96272786	132.707031	51.8479818	11.4664714	0.23730469
actin	6119.48275	8890.80632	10739.6051	13683.8854	12349.1038
	10348.1628	10186.0664	12481.2669	11939.0433	12211.8359
average	8233.82275	9538.43636	11610.436	12811.4644	12280.4699
normalized to actin	0.00021163	0.01142288	0.00351521	0.00119255	8.9268E-05
	0.00018967	0.01302829	0.00415406	0.00096042	1.9432E-05
average	0.00020065	0.01222558	0.00383464	0.00107648	5.435E-05
normalized to polyIC *100	1.73106677	93.4342088	28.7528894	9.75452465	0.73017138
	1.55141226	106.565791	33.9784503	7.85580413	0.15894825
average	1.64123952	100	31.3656699	8.80516439	0.44455982

VM-derived neural cells 24 hours after treatment with different doses of deucravacitinib, bariciti
rmalized to the housekeeping protein beta actin (ACTB).

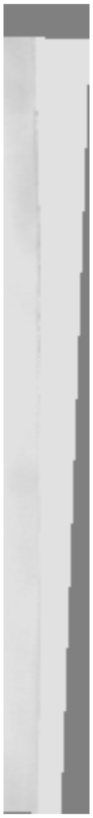

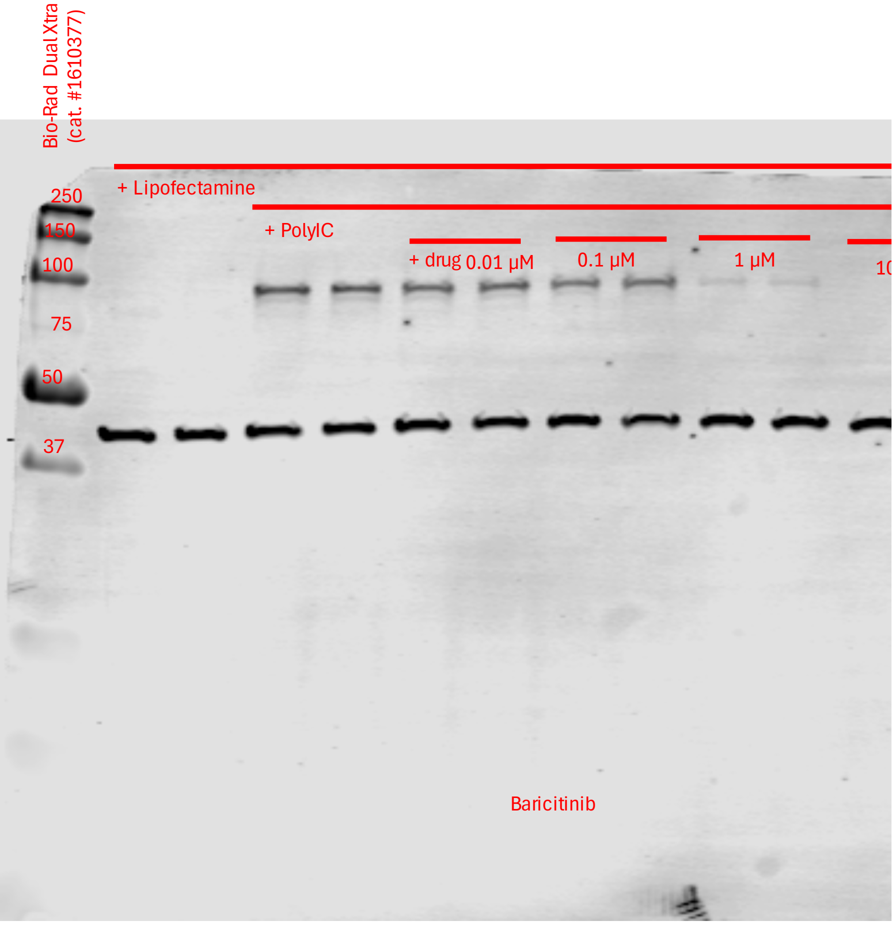

nnotations stay the same):

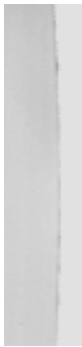

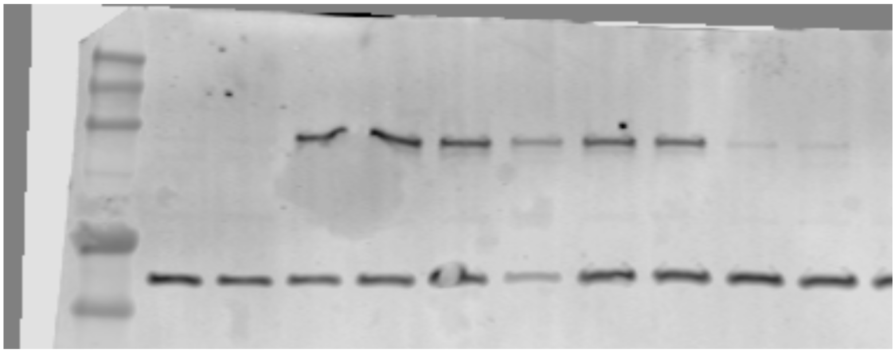

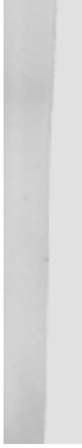

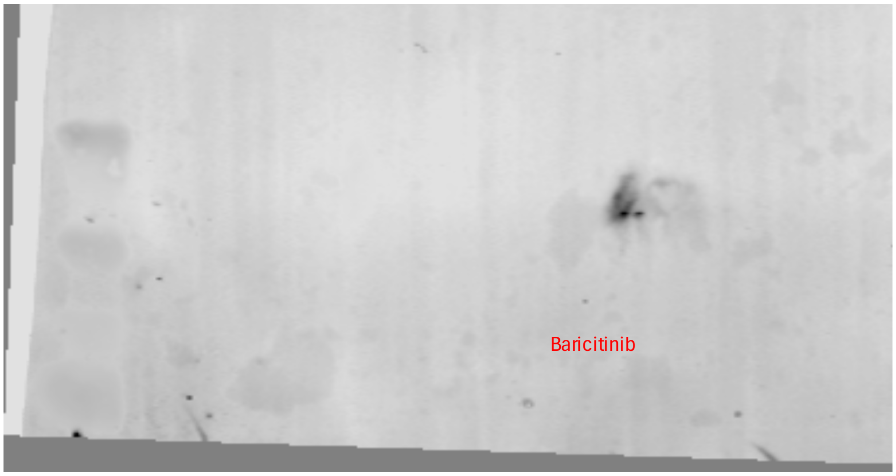
Baricitinib

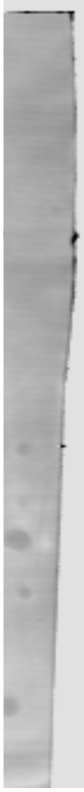

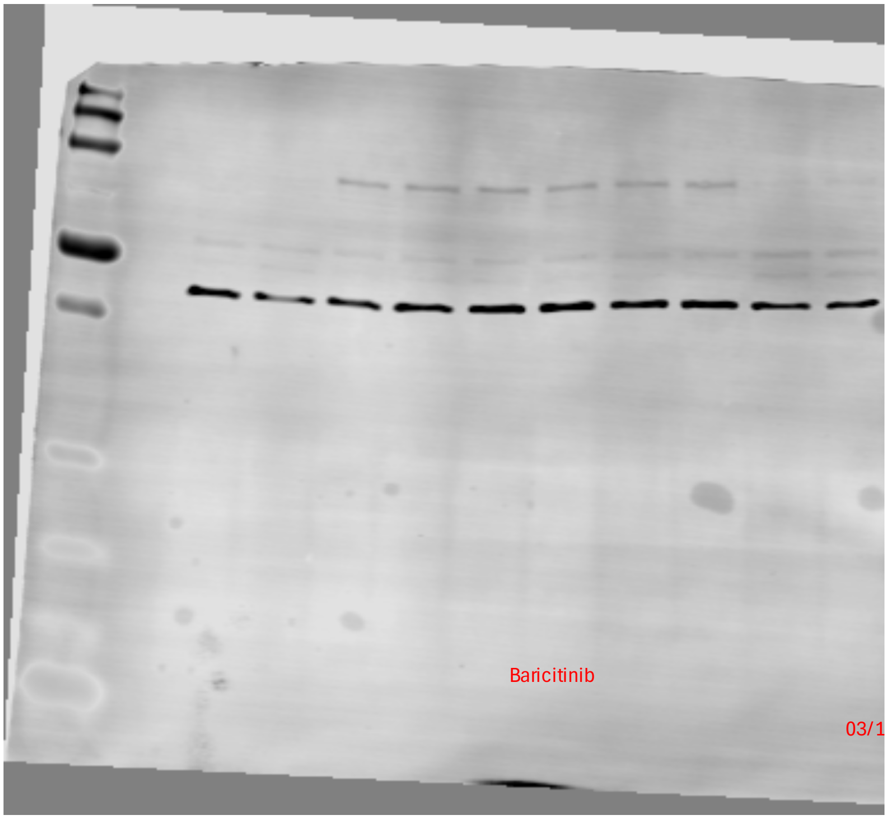
Baricitinib
03/1

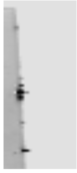

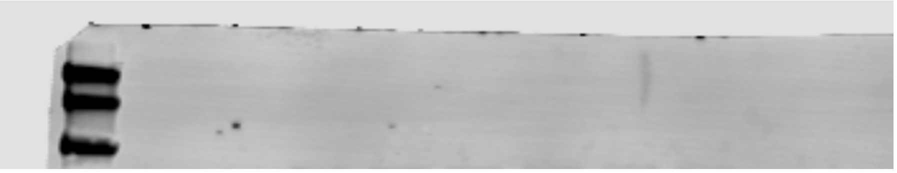

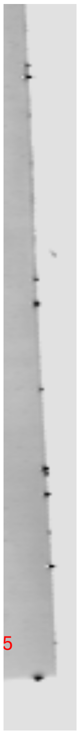
5

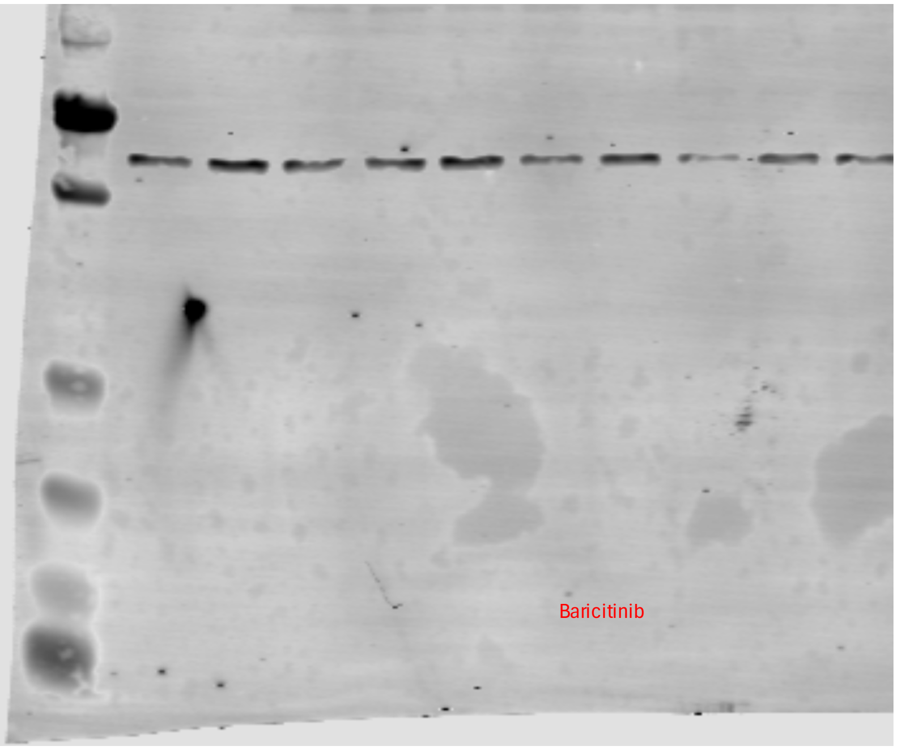
Baricitinib

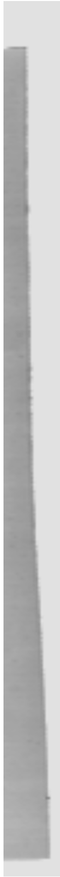

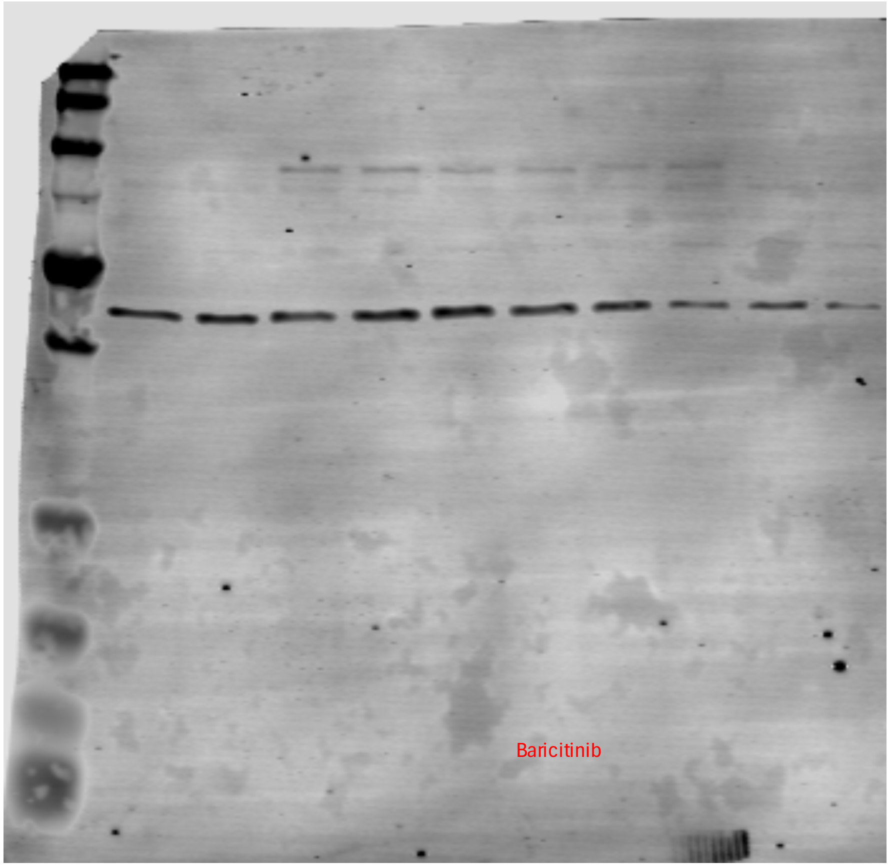
Baricitinib

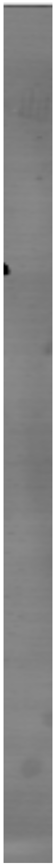

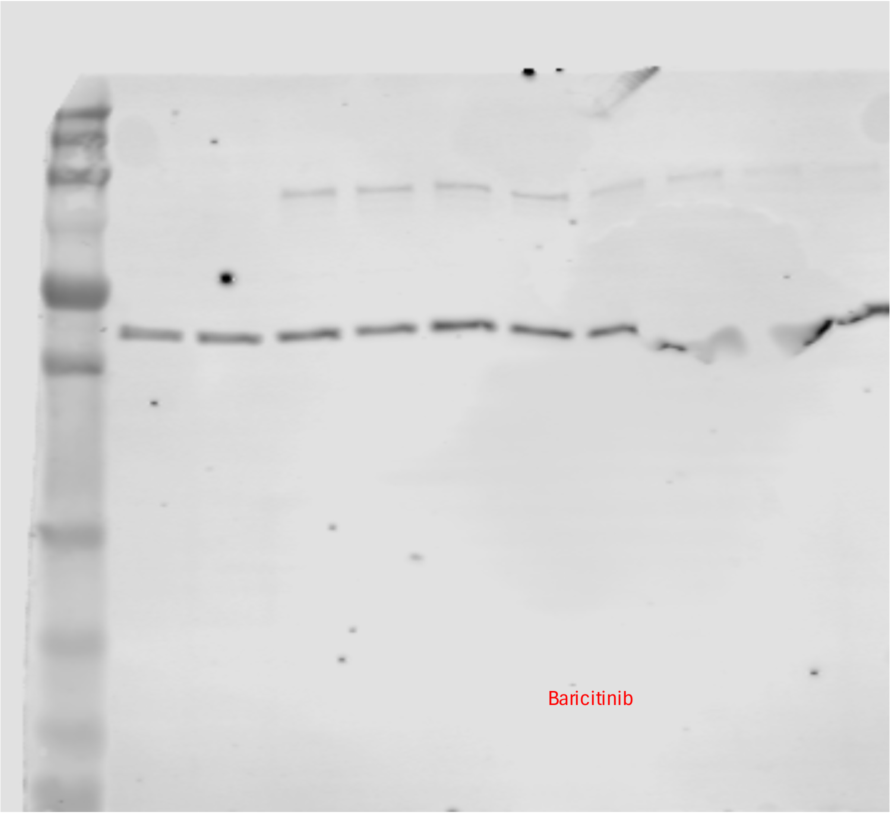

Baricitinib

Quantification of bands with ImageStudioLite

Blot 1

	ctrl	polyIC	0.01 µM	10 µM
pSTAT1	2.17724609	54.7744954	56.0672201	3.14746094
	1.81510417	46.5219727	41.2346191	4.18359375
actin	2156.74642	2964.46484	3928.96615	4583.82617
	2450.22103	2186.52832	3102.10807	4842.87402
average	2303.48372	2575.49658	3515.53711	4713.3501
normalized to actin	0.0010095	0.01847703	0.01427022	0.00068664
	0.00074079	0.02127664	0.01329245	0.00086387
average	0.00087515	0.01987683	0.01378134	0.00077526
normalized to polyIC *100	5.0788017	92.9576002	71.7932409	2.83052288
	3.72691178	107.0424	66.8740901	3.56107233
average	4.40285674	100	69.3336655	3.19579761

10 µM
2.77832031
1.05712891
4731.22168
2621.8877
3676.55469
0.00058723
0.00040319
0.00049521
16.153705
11.0911606
13.6224328

Blot 2

	ctrl	polyIC	0.01 µM
pSTAT1	5.04296875	30.5429688	20.3828125
	5.05859375	22.6171875	16.90625
actin	2190.91992	1759.41406	2232.68359
	1975.80469	2351.06445	1776.73633
average	2083.3623	2055.23926	2004.70996
normalized to actin	0.00230176	0.01735974	0.00912929
	0.00256027	0.00961998	0.00951534
average	0.00243101	0.01348986	0.00932231
normalized to polyIC *100	17.0628819	128.687333	67.675199
	18.9792219	71.3126666	70.5369891
average	18.0210519	100	69.106094

10 µM
0.05989583
0.50520833
2262.26432
2808.70052
2535.48242
2.6476E-05
0.00017987
0.00010317
0.22000517
1.49466784
0.8573365

Blot 3

	ctrl	polyIC	0.01 µM
pSTAT1	3.77864583	24.8352865	14.3710938
	1.19986979	23.4876302	23.2753906
actin	2089.36979	1611.93359	2561.09701
	2602.31315	1959.20117	1608.44336
average	2345.84147	1785.56738	2084.77018
normalized to actin	0.00180851	0.01540714	0.0056113
	0.00046108	0.01198837	0.01447076
average	0.00113479	0.01369776	0.01004103
normalized to polyIC *100	13.2029649	112.479304	40.9651347
	3.3660856	87.5206959	105.643261
average	8.28452527	100	73.304198

10 µM
6.98828125
5.68359375
1974.82422
4596.55469
3285.68945
0.00353869
0.00123649
0.00238759
11.2493989
3.93077316

Blot 4

	ctrl	polyIC	0.01 µM
pSTAT1	2.99283854	85.828776	95.0182292
	6.72786458	89.2760417	60.2115885
actin	5749.68685	4088.9694	7659.31185
	3434.21387	5833.36589	7669.01042
average	4591.95036	4961.16764	7664.16113
normalized to actin	0.00052052	0.02099032	0.01240558
	0.00195907	0.01530438	0.00785129
average	0.0012398	0.01814735	0.01012843
normalized to polyIC *100	2.86830873	115.666037	68.3603029
	10.7953494	84.3339626	43.2640883

average	6.83182906	100	55.8121956		7.59008602

Blot 5

	ctrl	polyIC	0.01 µM	10 µM
pSTAT1	0.06787109	102.792969	99.0014648	2.93798828
	1.52148438	116.720703	42.3984375	4.27636719
actin	8431.38281	4841.84668	5500.36621	11636.959
	6355.40527	5857.15234	1618.64648	10746.2217
average	7393.39404	5349.49951	3559.50635	11191.5903
normalized to actin	8.0498E-06	0.02123012	0.01799907	0.00025247
	0.0002394	0.01992789	0.02619376	0.00039794
average	0.00012372	0.020579	0.02209641	0.00032521
normalized to polyIC *100	0.03911665	103.163962	87.4632583	2.3249148
	1.16332185	96.836038	127.283902	3.66450805
average	0.60121925	100	107.37358	2.99471143

Blot 6

	ctrl	polyIC	0.01 µM	10 µM
pSTAT1	0.76204427	110.336263	88.1650391	1.23144531
	0.10546875	96.7242839	85.5143229	1.39005534
actin	10391.1641	7224.43424	7827.41667	11402.8711
	8411.2474	7059.16016	7024.39974	11552.8522
average	9401.20573	7141.7972	7425.9082	11477.8617
normalized to actin	7.3336E-05	0.01527265	0.01126362	0.00010799
	1.2539E-05	0.01370195	0.0121739	0.00012032
average	4.2937E-05	0.0144873	0.01171876	0.00011416
normalized to polyIC *100	0.50620739	105.420946	77.7482157	0.88334692
	0.08655175	94.5790543	84.0315013	0.9841771
average	0.29637957	100	80.8898585	0.93376201

nib or ruxolitinib, and transfection with poly(I:C)

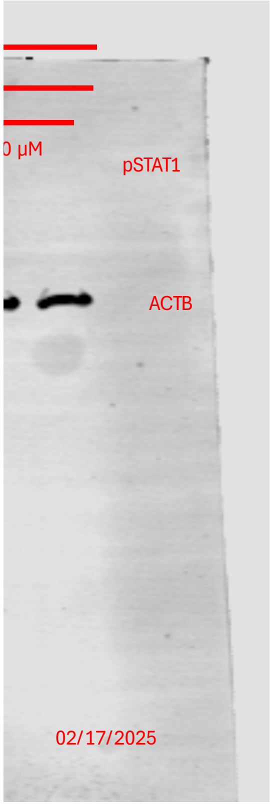

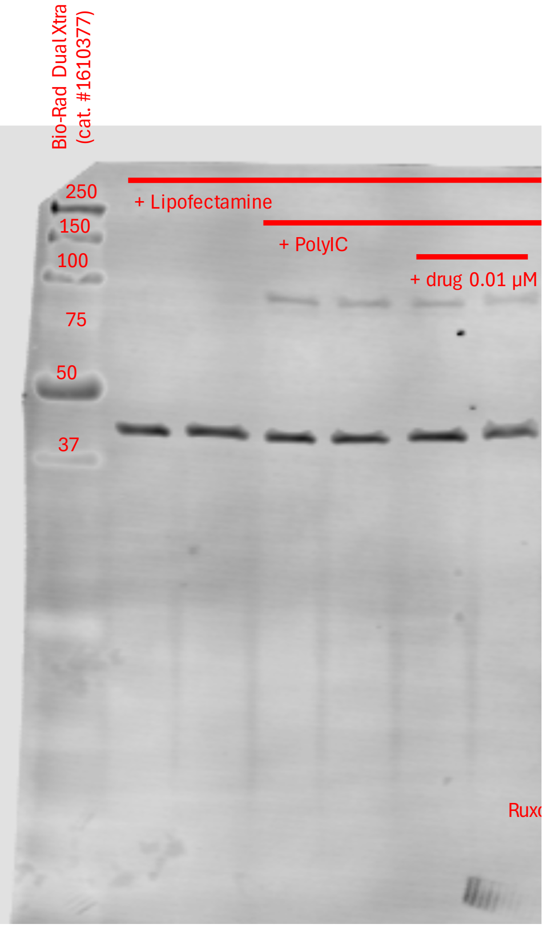

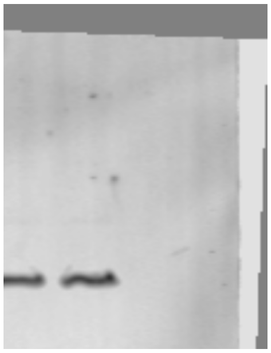

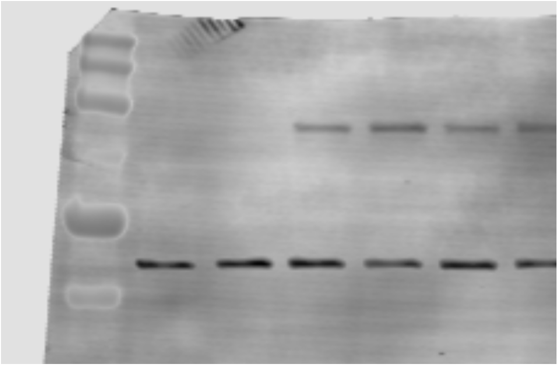

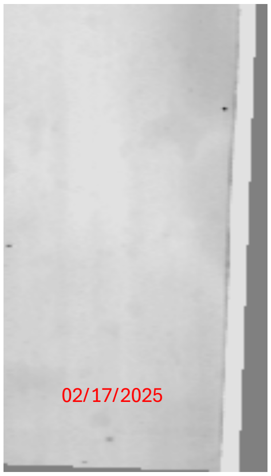
02/17/2025

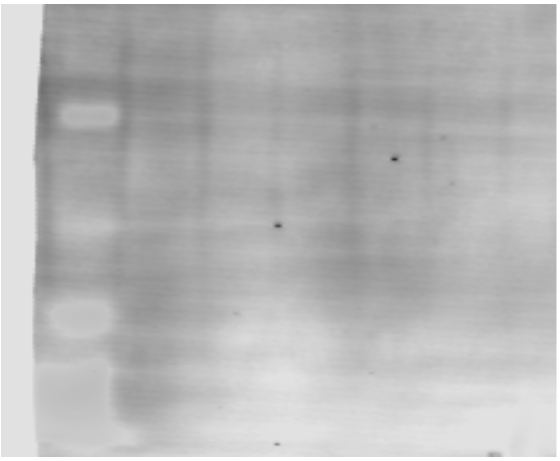

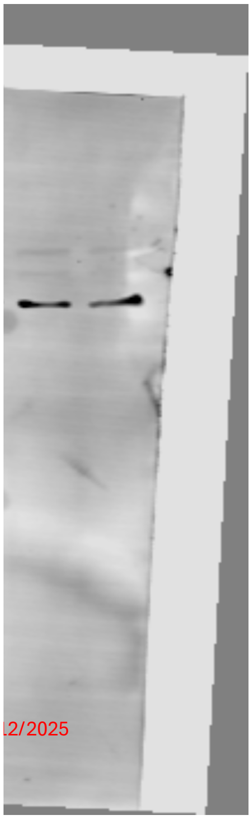
12/2025

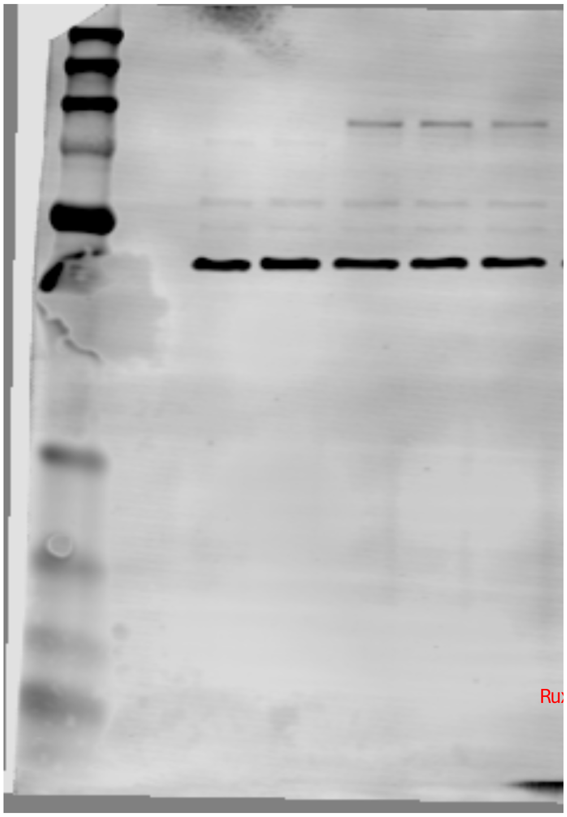

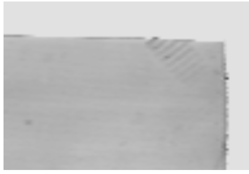

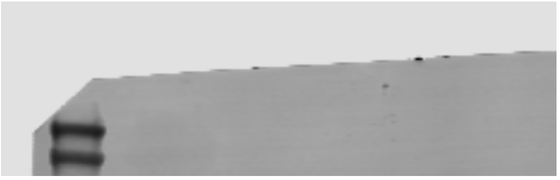

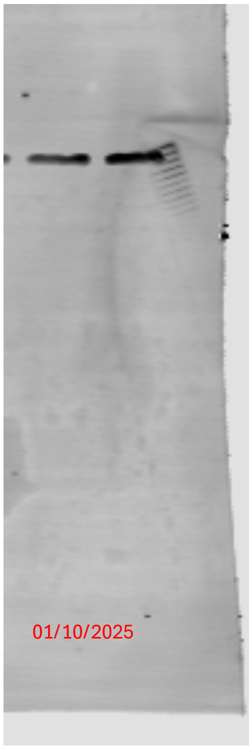
01/10/2025

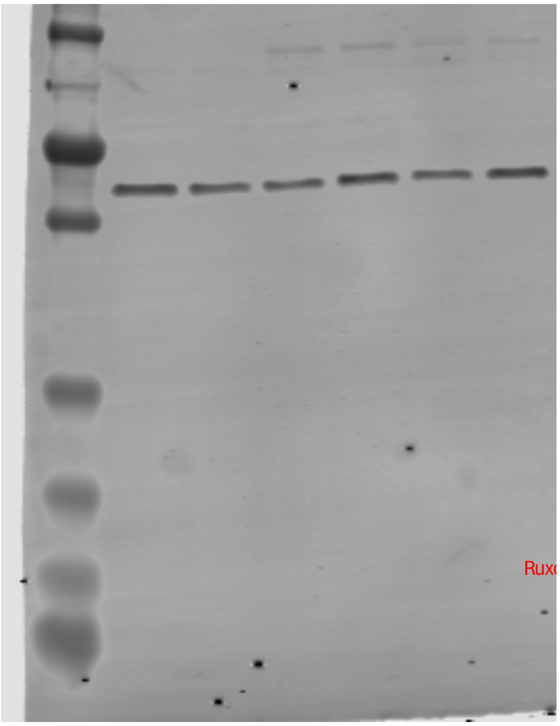
Rux

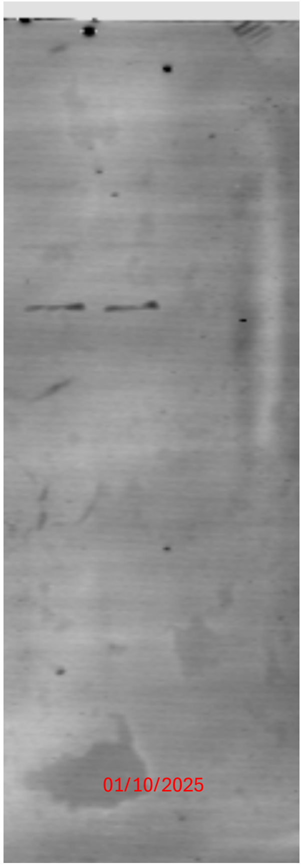
01/10/2025

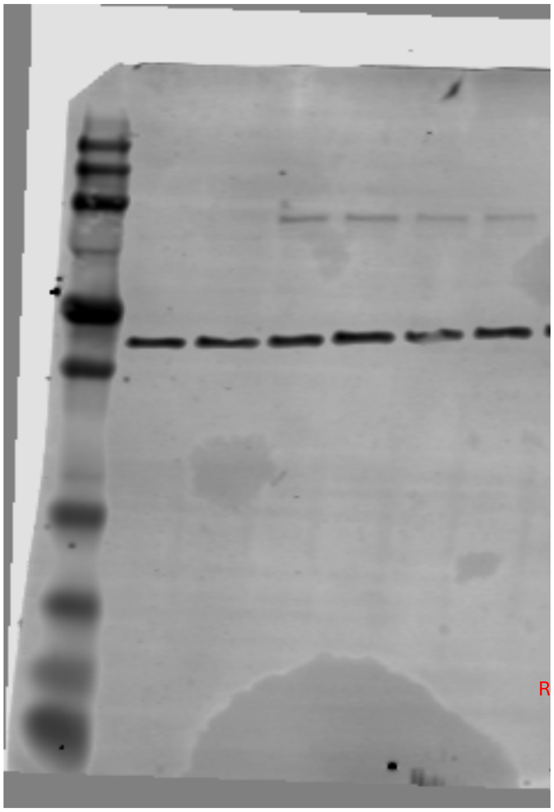

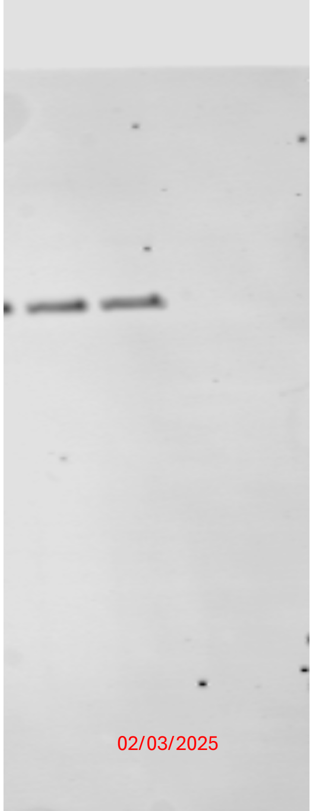

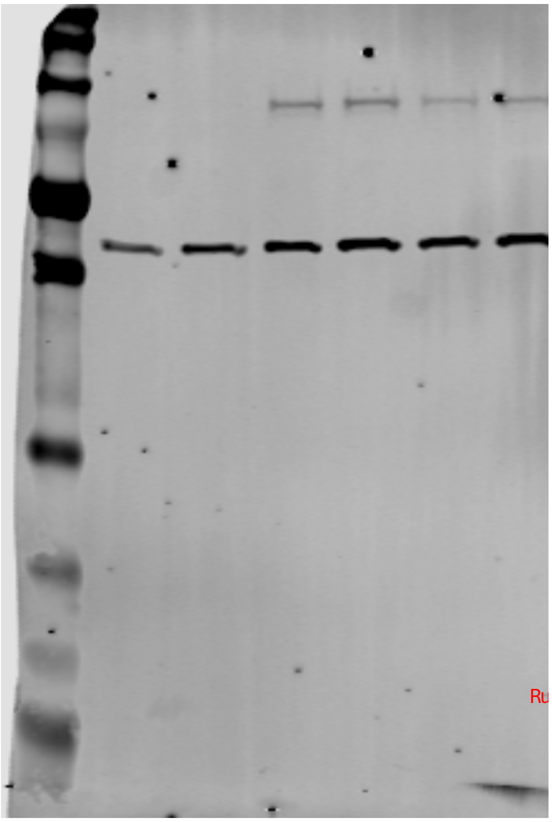

0.1 µM	1 µM	10 µM
29.0976563	12.3000488	2.06689453
37.7014974	10.1157227	2.36653646
2394.23796	2512.51855	3625.90007
2546.07292	4204.96387	3935.86947
2470.15544	3358.74121	3780.88477
0.0121532	0.00489551	0.00057004
0.01480771	0.00240566	0.00060127
0.01348045	0.00365058	0.00058566
61.1425461	24.6292042	2.86784258
74.4973088	12.1028449	3.02499969
67.8199275	18.3660246	2.94642114

Quantification of bands with ImageStudic

Blot 1

	ctrl
pSTAT1	0.04703776
	0.30224609
actin	1260.9401
	1641.05534
average	1450.99772
normalized to actin	3.7304E-05
	0.00018418
average	0.00011074
normalized to polyIC *100	0.17898551
	0.88369654
average	0.53134102

Blot 2

0.1 µM	1 µM	10 µM		ctrl
15.4042969	7.69726563	0.375	pSTAT1	4.515625
14.828125	8.46484375	3.38671875		10.6206055
1598.26172	1304.41602	980.677734	actin	2766.03516
1229.90625	913.080078	944.664063		2596.97363
1414.08398	1108.74805	962.670898	average	2681.50439
0.00963816	0.00590093	0.00038239	normalized to actin	0.00163253
0.01205631	0.00927065	0.0035851		0.00408961
0.01084723	0.00758579	0.00198375	average	0.00286107
71.4474273	43.7434431	2.83463774	normalized to polyIC *100	13.0825906
89.3731043	68.7230921	26.5762898		32.7729334
80.4102658	56.2332676	14.7054638	average	22.927762

Blot 3

0.1 µM	1 µM	10 µM		ctrl
23.1484375	8.39648438	1.94726563	pSTAT1	1.66210938
18.1777344	7.8671875	2.98372396		0.50260417
1989.69336	1666.23047	2405.94076	actin	3153.17578
1129.17253	1549.52018	3339.18099		2072.37435
1559.43294	1607.87533	2872.56087	average	2612.77507
0.01163417	0.00503921	0.00080936	normalized to actin	0.00052712
0.01609828	0.00507718	0.00089355		0.00024253
0.01386623	0.00505819	0.00085145	average	0.00038482
84.9348892	36.7885784	5.90868533	normalized to polyIC *100	4.01887697
117.524941	37.0657555	6.5233289		1.84906071
101.229915	36.927167	6.21600711	average	2.93396884

Blot 4

0.1 µM	1 µM	10 µM		ctrl
70.6673177	9.85481771	22.4388021	pSTAT1	7.40852865
93.5748698	22.5742188	22.5016276		9.60221354
6006.94759	4945.94694	4242.69857	actin	3244.93229
6787.5752	4904.1888	4328.87858		3736.97168
6397.26139	4925.06787	4285.78857	average	3490.95199
0.01176426	0.0019925	0.0052888	normalized to actin	0.00228311
0.0137862	0.00460305	0.00519803		0.00256952
0.01277523	0.00329778	0.00524342	average	0.00242631
64.826349	10.9795854	29.1436732	normalized to polyIC *100	6.20023366
75.9681195	25.3648528	28.6434501		6.97803727

70.3972342	18.1722191	28.8935617

average	6.58913547

0.1 µM	1 µM	10 µM
83.5268555	8.1640625	1.32861328
84.3725586	8.67285156	0.16601563
7430.68652	8480.90234	7044.47656
7392.75879	7511.69531	7901.68652
7411.72266	7996.29883	7473.08154
0.0112408	0.00096264	0.0001886
0.01141287	0.00115458	2.101E-05
0.01132683	0.00105861	0.00010481
54.6226579	4.67778111	0.91648529
55.4587811	5.61047442	0.10209508
55.0407195	5.14412776	0.50929018

Blot 5

	ctrl
pSTAT1	2.14550781
	0.37109375
actin	17434.9668
	17658.0234
average	17546.4951
normalized to actin	0.00012306
	2.1016E-05
average	7.2037E-05
normalized to polyIC *100	1.50985914
	0.25785111
average	0.88385513

0.1 µM	1 µM	10 µM
66.6835938	8.41341146	0.74023438
70.9322917	8.44173177	0.1328125
7974.4401	8418.30404	8863.07292
5971.22005	8269.83854	9087.08073
6972.83008	8344.07129	8975.07682
0.00836217	0.00099942	8.3519E-05
0.01187903	0.00102079	1.4616E-05
0.0101206	0.0010101	4.9067E-05
57.7206584	6.89858471	0.57649747
81.9961374	7.04607072	0.1008851
69.8583979	6.97232771	0.33869129

Blot 6

	ctrl
pSTAT1	3.54947917
	0.90169271
actin	11701.278
	13163.21
average	12432.244
normalized to actin	0.00030334
	6.8501E-05
average	0.00018592
normalized to polyIC *100	3.51037115
	0.79271751
average	2.15154433

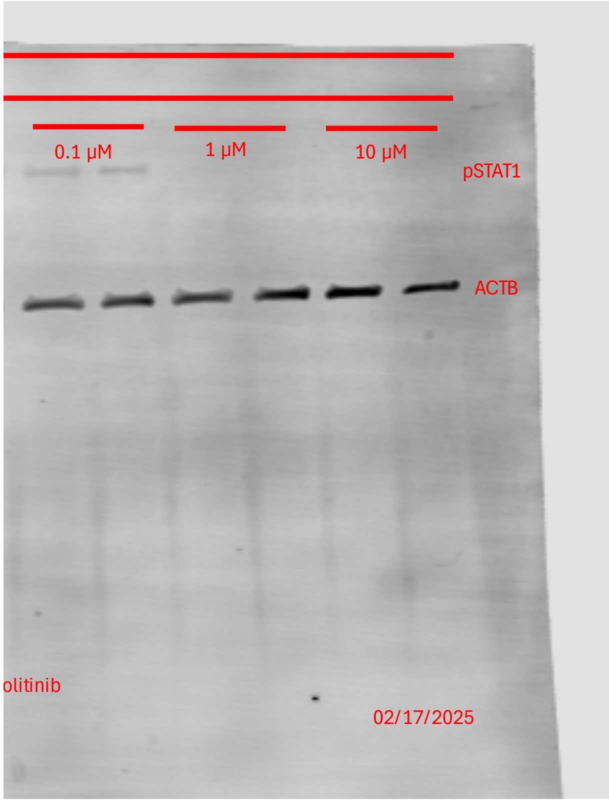
0.1 µM
1 µM
10 µM
pSTAT1
ACTB
olitinib
02/17/2025

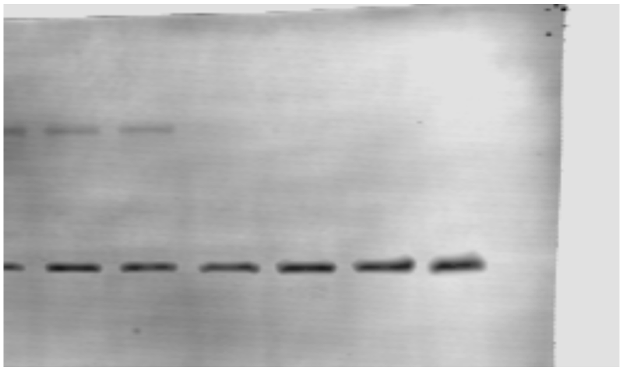

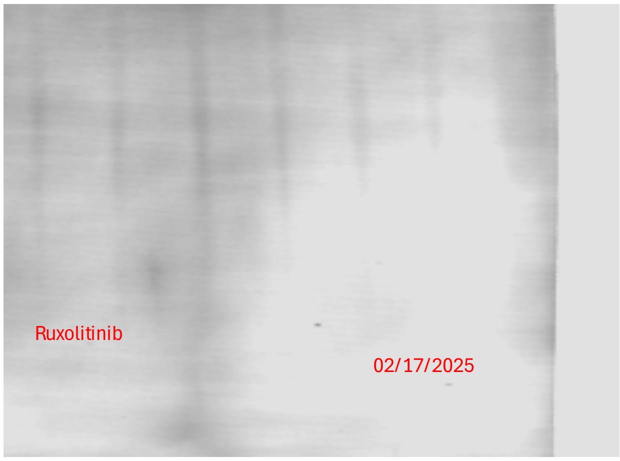
Ruxolitinib
02/17/2025

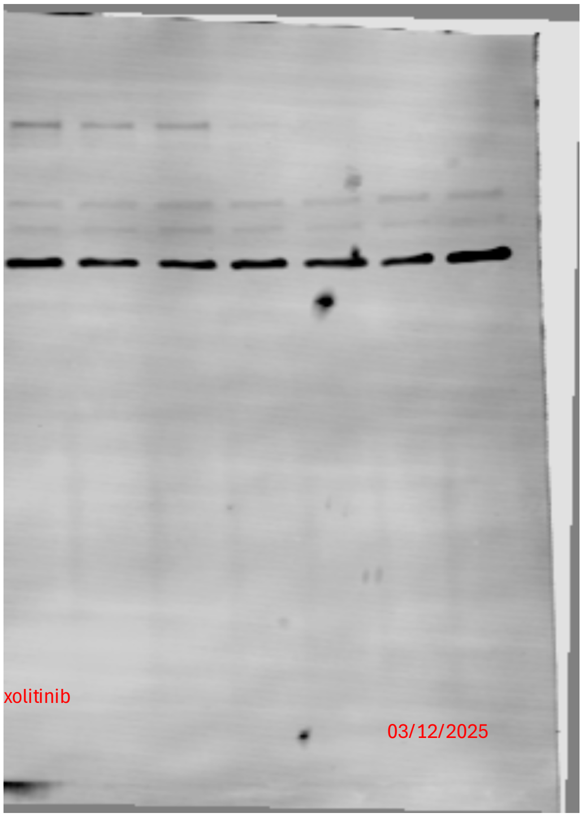
xolitinib
03/12/2025

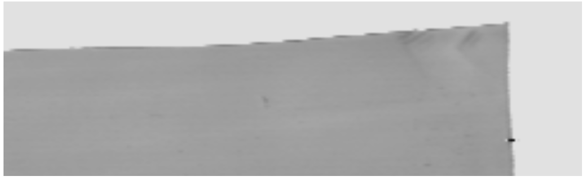

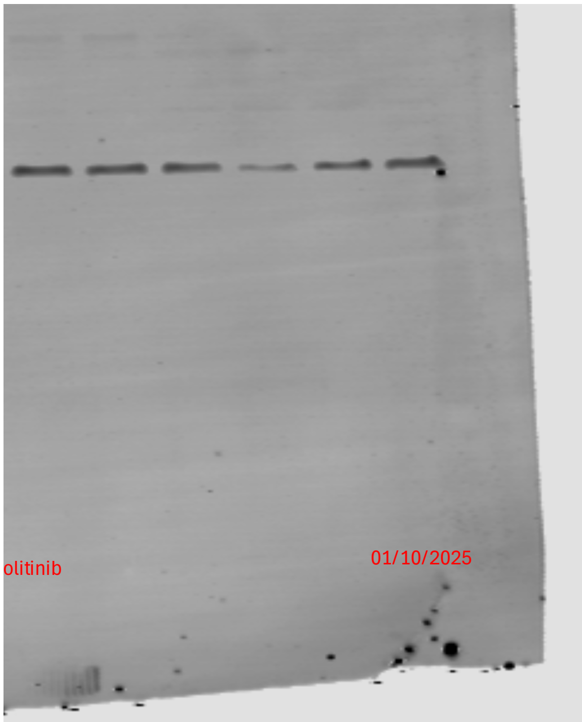
olitinib
01/10/2025

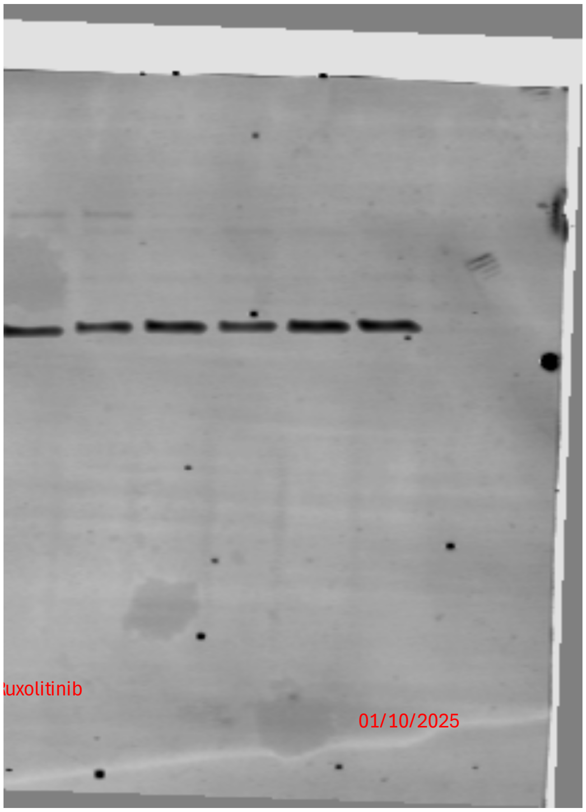
uxolitinib
01/10/2025

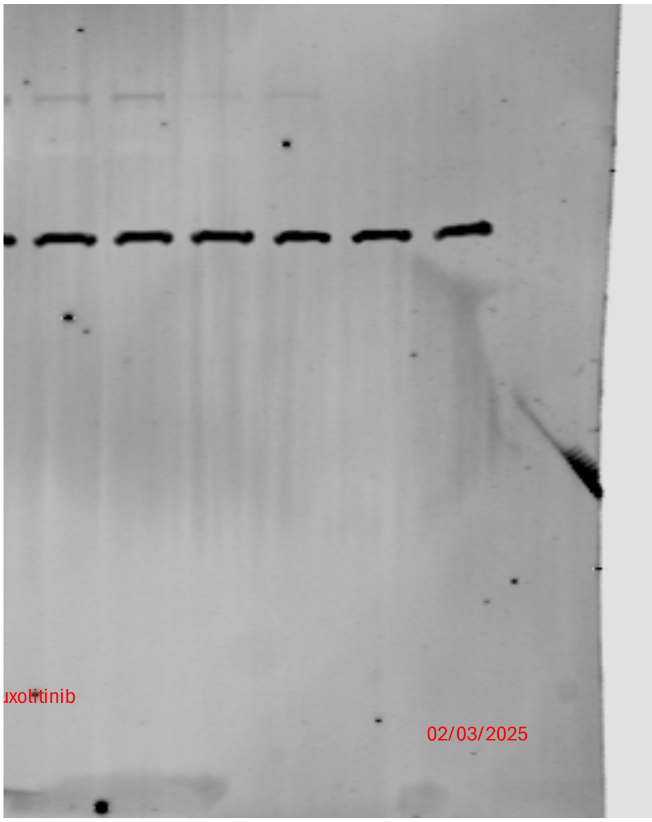

Ruxolitinib

Lite

polyIC	0.01 µM	0.1 µM	1 µM	10 µM
47.1936849	25.0592448	15.3880208	4.68977865	0.03222656
51.8603516	25.2361654	16.8541667	5.30224609	1.67626953
2110.88151	1958.81445	2081.66927	2206.61784	2518.05078
2683.42513	2243.94466	2049.16211	2610.72656	2380.17643
2397.15332	2101.37956	2065.41569	2408.6722	2449.11361
0.02235733	0.01279307	0.00739215	0.00212532	1.2798E-05
0.01932618	0.01124634	0.00822491	0.00203095	0.00070426
0.02084176	0.0120197	0.00780853	0.00207814	0.00035853
107.271837	61.381905	35.4680015	10.197434	0.06140662
92.7281633	53.9606169	39.4635955	9.74460359	3.37909486
100	57.6712609	37.4657985	9.97101881	1.72025074

polyIC	0.01 µM	0.1 µM	1 µM	10 µM
34.434082	21.2504883	9.90527344	2.9140625	2.84082031
37.6655273	20.3144531	9.54345703	1.22949219	0.47998047
2660.03418	1800.03223	2400.33496	3032.44629	3553.06934
3135.5918	2647.80078	1984.3252	2011.79883	3390.41113
2897.81299	2223.9165	2192.33008	2522.12256	3471.74023
0.01294498	0.01180562	0.00412662	0.00096096	0.00079954
0.01201225	0.0076722	0.00480942	0.00061114	0.00014157
0.01247862	0.00973891	0.00446802	0.00078605	0.00047055
103.737284	94.6067694	33.0695433	7.70086171	6.40727939
96.2627163	61.4827676	38.5413085	4.89750403	1.13450073
100	78.0447685	35.8054259	6.29918287	3.77089006

polyIC	0.01 µM	0.1 µM	1 µM	10 µM
29.9101563	8.52539063	9.17773438	8.75260417	2.109375
29.9290365	11.0716146	15.7851563	7.25260417	1.28450521
1896.99935	1881.83138	2710.88932	2058.77018	1911.45573
2859.85417	2975.18424	2459.43229	1023.79102	2600.50521
2378.42676	2428.50781	2585.16081	1541.2806	2255.98047
0.01576709	0.00453037	0.00338551	0.00425138	0.00110354
0.01046523	0.00372132	0.00641821	0.00708407	0.00049394
0.01311616	0.00412584	0.00490186	0.00566772	0.00079874
120.211162	34.5403612	25.8117227	32.41326	8.41361906
79.7888383	28.3720285	48.9336159	54.0102211	3.765923
100	31.4561949	37.3726693	43.2117406	6.08977103

polyIC	0.01 µM	0.1 µM	1 µM	10 µM
96.7978516	77.6634115	36.6009115	14.1699219	3.49902344
99.6914063	86.8007813	38.4407552	10.4108073	8.48339844
2623.41211	2435.24902	1675.48405	2087.75293	1742.38249
2712.82747	2286.19206	1873.78646	1960.55859	4340.71517
2668.11979	2360.72054	1774.63525	2024.15576	3041.54883
0.03689769	0.03189136	0.02184498	0.00678716	0.00200818
0.03674816	0.03796741	0.02051501	0.00531012	0.00195438
0.03682292	0.03492938	0.02118	0.00604864	0.00198128
100.203048	86.6073633	59.3243972	18.4318991	5.45362266
99.7969523	103.108069	55.7126154	14.420699	5.30750372

100	94.8577162	57.5185063	16.4262991	5.38056319

polyIC	0.01 µM	0.1 µM	1 µM	10 µM
103.607422	94.8720703	69.0458984	20.6660156	3.43164063
133.47168	81.0732422	62.8867188	10.4003906	4.82128906
17366.4453	17929.6875	17213.8887	13778.9863	16305.2207
12915.0234	14555.1309	14873.9199	17192.874	14834.9375
15140.7344	16242.4092	16043.9043	15485.9302	15570.0791
0.00596595	0.00529134	0.00401106	0.00149982	0.00021046
0.01033461	0.00557008	0.00422799	0.00060492	0.000325
0.00815028	0.00543071	0.00411952	0.00105237	0.00026773
73.199378	64.922183	49.2137379	18.4020811	2.5822755
126.800622	68.3421887	51.8753396	7.4221312	3.98753866
100	66.6321859	50.5445387	12.9121062	3.28490708

polyIC	0.01 µM	0.1 µM	1 µM	10 µM
99.7623698	73.6422526	60.398763	3.67643229	2.49707031
88.3544922	78.4101563	63.9433594	0.26888021	8.75683594
10468.2813	12732.2819	13001.6367	13258.7301	17105.8057
11396.7529	10994.6221	12421.4255	15158.4082	13335.9945
10932.5171	11863.452	12711.5311	14208.5692	15220.9001
0.00952997	0.0057839	0.00464547	0.00027728	0.00014598
0.0077526	0.00713168	0.00514783	1.7738E-05	0.00065663
0.00864128	0.00645779	0.00489665	0.00014751	0.0004013
110.284144	66.9333438	53.7590666	3.20882729	1.68930839
89.7158558	82.5303556	59.5724847	0.20527069	7.59877367
100	74.7318497	56.6657756	1.70704899	4.64404103

Supplementary Figure 8h: Image and quantification of Western blot of STING in SH-

anti-pSTAT1

plotted values in red, black dotted rectangules correspond to shown images in manuscript, red c

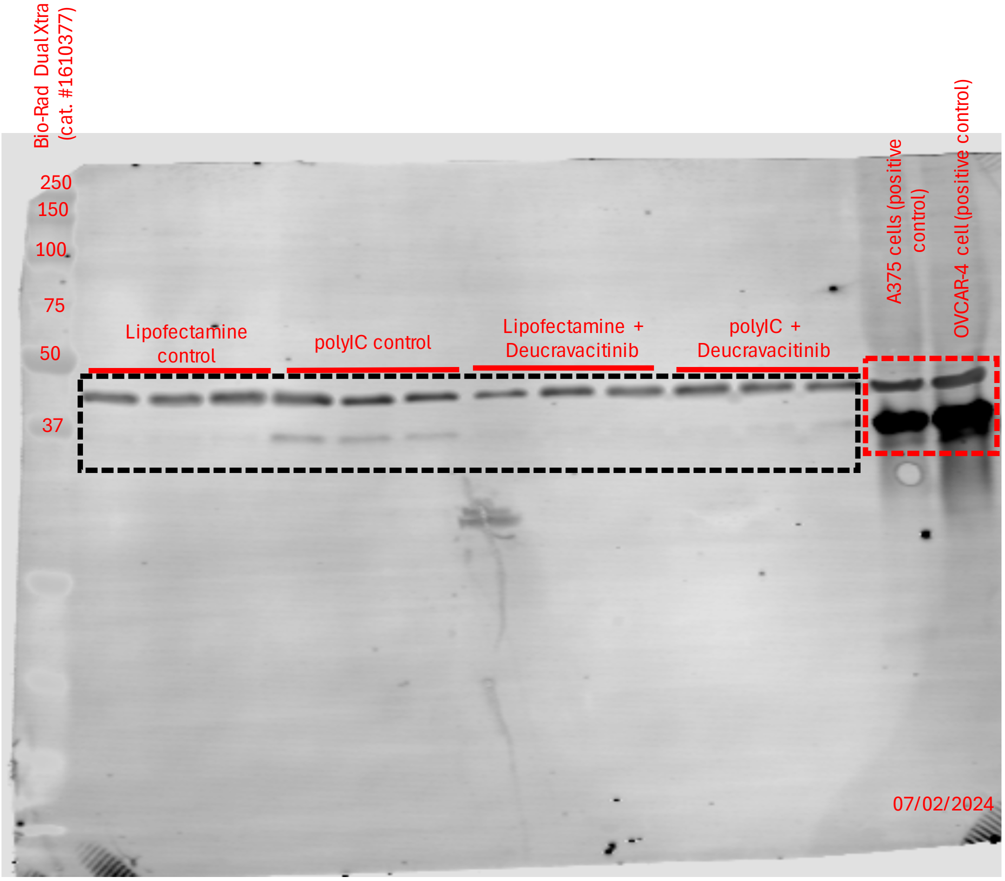

Supplement: Supplementary file 6 — Source Data [file 41467_2026_70243_MOESM6_ESM.pdf]
